# Engineering DNA Origami Captors for TGFβ1 Sequestration to Enhance Tumor Immune Modulation and Therapy

**DOI:** 10.1002/advs.202506827

**Published:** 2025-07-11

**Authors:** Xiao Chen, Dunfang Liu, Jiahui Jin, Han Yao, Yao Sheng, Yarong Liu, Jingwei Sun, Yang Yang

**Affiliations:** ^1^ Institute of Molecular Medicine and Shanghai Key Laboratory for Nucleic Acid Chemistry and Nanomedicine State Key Laboratory of Oncogenes and Related Genes Renji Hospital School of Medicine Shanghai Jiao Tong University Shanghai 200127 China; ^2^ Grit Biotechnology Co., Ltd Shanghai 200003 China; ^3^ Department of Nuclear Medicine Renji Hospital School of Medicine Shanghai Jiao Tong University Shanghai 200127 China; ^4^ Shanghai Key Laboratory of Gut Microecology and Associated Diseases Department of Gastroenterology Shanghai Ninth People's Hospital School of Medicine Shanghai Jiao Tong University Shanghai 200023 China

**Keywords:** DNA origami framework, rapid cytokine clearance, systemic immune regulation, TGFβ sequestration, tumor immunotherapy

## Abstract

Efficient modulation of pivotal immune‐regulatory molecules to leverage the tumor microenvironment (TME) and enhance therapeutic effects remains an ideal yet challenging goal. TGFβ1 represents a critical therapeutic target as a key cytokine involved in immune suppression and tumor progression. Here, a DNA origami‐based framework functionalized with anti‐TGFβ1 aptamers is developed to act as a captor for efficient TGFβ1 sequestration and fast clearance, thereby improving anti‐tumor immunity. By engineering the geometric shapes and pore sizes of three DNA framework captors (DFCs), the superior efficacy of a barrel‐shaped captor (DBC) in regulating TGFβ1 levels is demonstrated. In cell culture, DBCs significantly enhance T cell‐mediated tumor cytotoxicity, while systemic administration effectively inhibits tumor growth in the mouse model. Moreover, DBCs demonstrate a synergistic effect with PD‐L1 antibody to enhance anti‐tumor efficacy. Immunohistochemistry (IHC) further confirmes the DBC‐mediated reduction of TGFβ in tumor tissue and its biodistribution fate. These findings underscore the importance of cytokine regulation in cancer immunotherapy and provide valuable insights for the rational design and application of structural DNA nano‐devices as a transformative tool for precise immunomodulation.

## Graphical abstract

Programmable DNA framework captors effectively deplete circulatingtransforming growth factor‐β1 (TGFβ1) through fast systemic clearance and reshape the tumor immune microenvironment, alleviating immunosuppression to inhibit tumor progression.

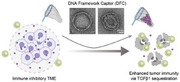

## 1 Introduction

A critical mechanism of tumor progression is the ability of cancer cells to produce factors that induce self‐propagating growth and create an immunosuppressive tumor microenvironment (TME).^[^ ^1^ ^]^ Among these factors, transforming growth factor‐β1 (TGFβ1) plays a pivotal role in the functional regulation of both tumor cells and immune cells.^[^ ^2^ ^]^ In the tumor microenvironment (TME), several types of cells are known to release TGFβ1, such as tumor cells, cancer‐associated fibroblasts as well as immune subsets including regulatory T cells (Tregs), myeloid‐derived suppressor cells (MDSCs), and tumor‐associated macrophages (TAMs). Over expressed TGFβ1 not only promotes epithelial‐to‐mesenchymal transition, invasion, and metastasis of tumor cells,^[^ ^1a^ ^]^ but also regulates the adaptive immune system by promoting the differentiation of Tregs,^[^ ^3^ ^]^ suppressing TH1 differentiation, inhibiting CD8+ T cell activation, and dampening the cytotoxicity of conventional T cells, thus facilitating immune evasion and tumor progression.^[^ ^3^, ^4^ ^]^ The low molecular concentration of TGFβ1 (100–150 pM in circulation, up to 0.5–1 nm in the tumor microenvironment.^[^ ^5^ ^]^) further underscores its substantial biological impact. The essential role of TGFβ1 in tumor progression and immune modulation makes it a critical target for therapeutic interventions.

Building on this premise, various strategies have been explored to block TGFβ1 signaling, using small‐molecule inhibitors,^[^ ^6^ ^]^ mono‐ or bispecific antibodies,^[^ ^5^, ^7^ ^]^ and fusion proteins.^[^ ^8^ ^]^ Since these approaches primarily rely on equimolar molecular recognition and interaction to adequately inhibit TGFβ1 or its receptor, they often require high drug doses and prolonged circulation to achieve efficacy, which can lead to liver burden and dose‐accumulative toxicity.^[^ ^1^, ^9^ ^]^ (summarized in Table S1, Supporting Information). In contrast to inhibition, a capture‐based approach employing nano “trashcans” to efficiently sequester dozens to hundreds of TGFβ1 molecules within each confined structural compartment from the bloodstream, followed by their rapid clearance, represents an ideal strategy for eliminating TGFβ1‐mediated biological effects. This high‐capacity capture mechanism enables a dramatic reduction in the required dosage of the nano‐device, addressing challenges associated with conventional therapies. While this paradigm‐shifting method of cytokine regulation remains largely unexplored in tumor immunotherapy research and clinical applications, recent advancements in DNA nanotechnology pave the way for its implementation.

**Figure 1:**
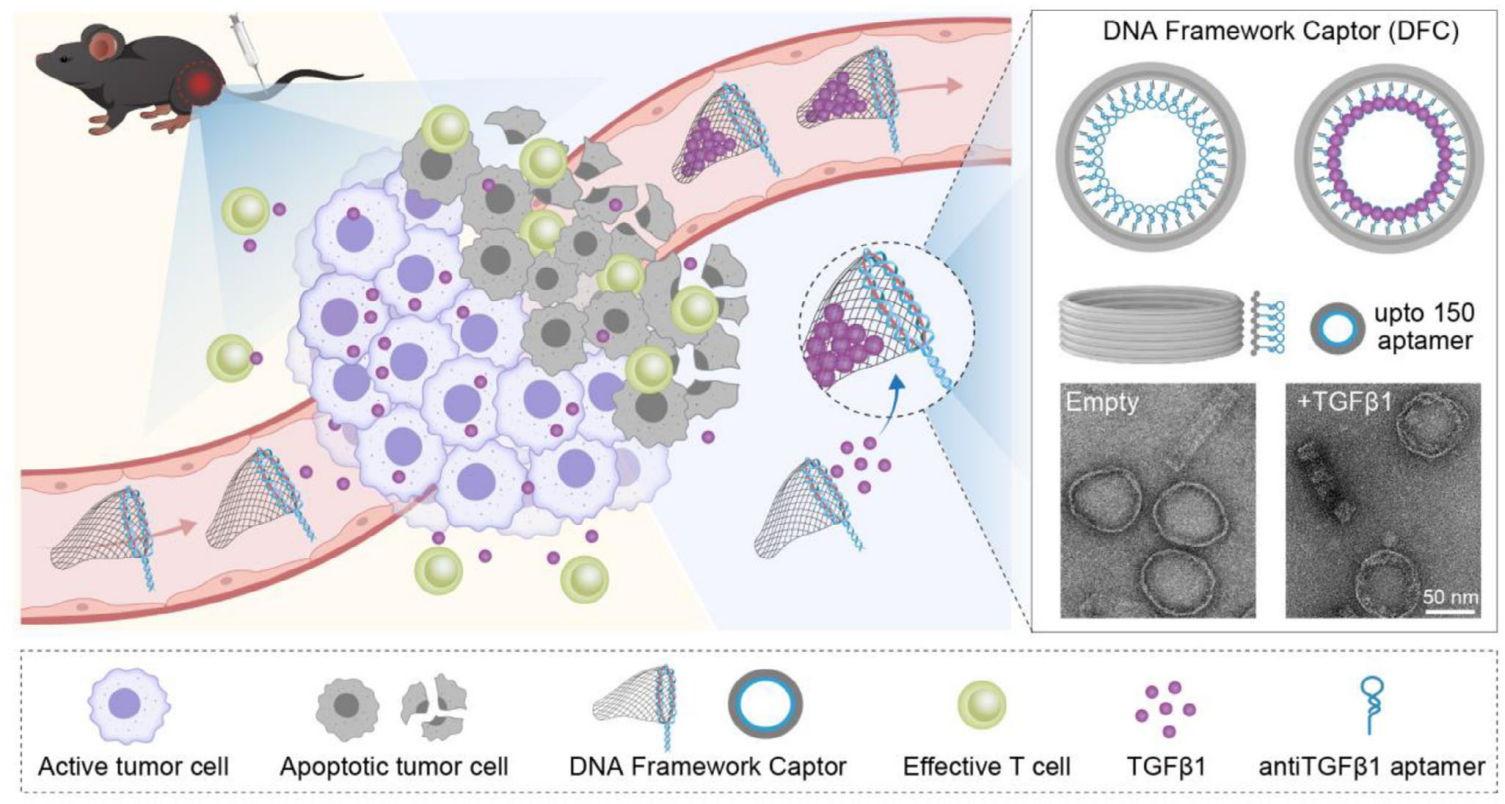
Schematic illustration of the rapid TGFβ1 removal process for tumor immune modulation using DNA framework captor. The barrel‐shaped DFC, identified as the most efficient candidate, is depicted in the right panel. Transmission electron microscopy (TEM) images show the structural morphology of the DFC before and after TGFβ1 capture.

With customizable geometry and programmable modification capabilities,^[^ ^10^ ^]^ functionalized DNA nanostructures offer versatile tools for addressing biomedical challenges. DNA origami structures can be tailored to specific applications, demonstrating exceptional in vivo properties such as minimal toxicity,^[^ ^11^ ^]^ low immunogenicity,^[^ ^11^, ^12^ ^]^ and tolerable bio‐stability.^[^ ^13^ ^]^ Notably, DNA nanostructures exhibit rapid clearance from circulation during metabolic processes,^[^ ^14^ ^]^ a characteristic that we hypothesize could aid in swiftly removing immunosuppressive cytokines like TGFβ1 from the bloodstream (**Figure** **1**). This rapid elimination feature could have profound implications for rebalancing cancer immunity.

In the emerging family of DNA nanostructures, framework nucleic acids (FNAs),^[^ ^15^ ^]^ especially 3D DNA origami frameworks that possess confined internal spaces and ready‐to‐decorate inner/outer surfaces, have been previously explored for diverse biomedical applications, including thrombin sensing and auto‐anticoagulation,^[^ ^16^ ^]^ phage genome packaging,^[^ ^17^ ^]^ virus‐mimetic vaccination,^[^ ^18^ ^]^ and siRNA delivery.^[^ ^19^ ^]^ In this study, we equipped three DNA framework captors (DFCs) with distinct geometries to carry a TGFβ1‐specific capturing agent—a well‐established 75‐nt DNA aptamer featuring 35 phosphorothioate‐modified sites to enhance nuclease resistance and binding affinity.^[^ ^20^ ^]^ Leveraging the programmability of DNA nanostructures, the aptamer was assembled onto the frameworks with editable valency and spatial patterning to investigate how geometric confinement affects cytokine capture. These DFCs were designed to sequester and rapidly clear TGFβ1, thereby triggering anti‐tumor immune responses. At both cellular and animal levels, our results demonstrate that a rationally engineered DFC can effectively reverse TGFβ1‐mediated functional impact on immune responses and tumor growth.

## 2 Results

### 2.1 In Vitro Assessment of DFC Capturing Capability and Efficiency

**Figure 2:**
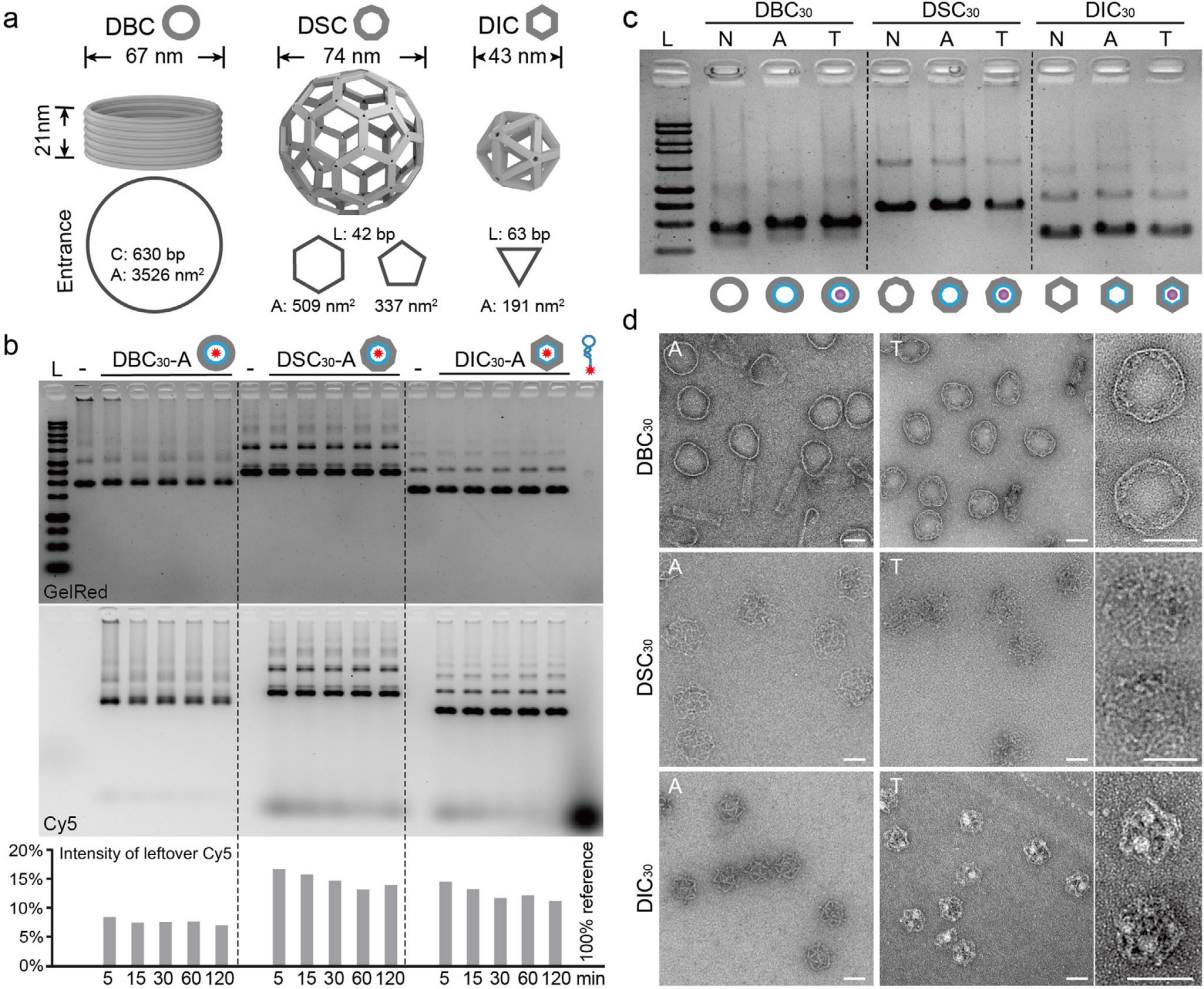
In vitro Assessment of DFC Capturing Capability and Efficiency. a) Schematic illustration of the geometric properties of the three DFCs. The shapes of their entrances are shown to scale, with edge lengths and areas listed. b) Agarose gel electrophoresis (AGE) results demonstrating the capture efficiency and kinetics of the three DFCs for Cy5‐a’‐Apt over a time range from 5 min to 2 h. c) AGE image showing the capture of recombinant human TGFβ1 by the three DFCs after 15 min of incubation. (N: non‐modified, A: aptamer‐modified, and T: TGFβ1‐incubated DFCs) d). TEM images of the three DFCs, either modified with aptamers (A) or capturing TGFβ1 (T) for 15 min. Two representative DFC‐T particles are enlarged and displayed on the right side. Scale bar = 50 nm.

Among the various geometric designs for constructing “captor” devices, capture efficiency is primarily influenced by binding affinity and the interaction probability and frequency based on molecular diffusion. In DNA origami frameworks, the number and local density of capturing agents determine the overall binding capacity, while the size and shape of entrances can influence the dynamics of target molecules accessing the entrapped agents within the nanoscale structure. To systematically evaluate these parameters and identify the most effective design, we developed three distinct DNA origami captors, as shown in **Figure** **2**a and Supplementary Figure S1 (Supporting Information), the DNA Barrel Captor (DBC), the DNA Soccer Captor (DSC), and the DNA Icosahedral Captor (DIC). All three structures were assembled using the same circular ssDNA scaffold (p7560), resulting in equivalent molecular weights, yet they exhibit distinct geometries, stiffness, entrance properties, and available modification sites (see staples information in Table S2, Supporting Information). Specifically, the DBC features a flat barrel shape formed by 12 interwoven helices in a honeycomb arrangement, measuring 21 nm in height and 67 nm in diameter. Five of the helices are tilted inward, enabling the extension of up to 30 handles per helix, providing a maximum of 150 potential modification sites. The DSC, designed as a truncated icosahedron with 90 edges (≈74 nm in diameter and 14 nm edge length), is larger and more flexible, with up to 90 handles available for internal modification. In contrast, the DIC forms a rigid icosahedron consisting of 30 edges (each 21 nm in length) built from four helices per edge, offering 30 inner handles for decoration via complementary oligonucleotide binding. A 21‐nt oligo (sequence a, as listed in Table S3, Supporting Information) was selected as the inner handle and could be extended from the available modification sites on each DFC.

Notably, these structures differ not only in geometry but also in the size and shape of their windows, which regulate molecular access. The DBC has the largest entrance area, with circular openings measuring ≈3526 nm^2^ on both the top and bottom surfaces. The DSC includes 20 hexagonal windows (509 nm^2^ each) and 12 pentagonal windows (337 nm^2^ each), while the DIC, the smallest structure, features 20 triangular windows with an area of 191 nm^2^ each (Figure 2a). These varying apertures allow molecules and proteins to enter and exit the nanoscale captors, influencing their capture capabilities.

To functionalize the interior of DFCs for molecular trapping, a well‐characterized anti‐TGFβ1 aptamer was employed.^[^ ^21^ ^]^ as a high‐affinity capturing agent. This 96‐nt engineered aptamer was extended at its 3′ end with the a’ sequence (a’‐Apt, see Table S3, Supporting Information) for DFC modification. The affinity and specificity of the a’‐Apt were validated prior to deployment. As shown in the native‐PAGE gel image in Figure S2 (Supporting Information), the appearance of slower‐mobility bands indicated the formation of tightly bound aptamer‐TGFβ1 complexes, while the disappearance of the free a’‐Apt band at a 1:1 molar ratio suggested equimolar recognition between the aptamer and TGFβ1. To further quantify binding affinity, we performed Microscale Thermophoresis (MST) using a Cy5‐labeled a′‐Apt to monitor fluorescence shifts associated with changes in thermophoretic mobility upon protein binding. The dissociation constant (*Kd*) for active TGFβ1 was determined to be 28 nm (Figure S3, Supporting Information). While this differs slightly from a previously reported value of 1.07 nm obtained by SPR,^[^ ^20a^ ^]^ the variation is likely due to differences in assay format, buffer conditions, and the presence of the 3′‐terminal extension in our construct. We also examined the aptamer's interaction with latent TGFβ1 and observed a much weaker binding affinity (*Kd* = 152 nm), suggesting limited but detectable recognition under in vitro conditions. In terms of specificity, MST results showed that the a′‐Apt exhibited no detectable binding to unrelated proteins such as IFN‐γ and BSA (Figure S3, Supporting Information). To evaluate cross‐reactivity with closely related cytokines, we referred to previous aptamer sensor studies, which demonstrated negligible binding to other TGFβ superfamily members, including TGFβ2 and TGFβ3.^[^ ^20a^ ^]^ Together, these data confirm the high affinity and excellent isoform‐level specificity of the a′‐Apt toward TGFβ1.

To confirm aptamer deployment efficiency and to evaluate the impact of structural properties on molecular transport within the framework structures, each captor was assembled to extend 30 inner handles to capture an equivalent number of Cy5‐a’‐Apt under identical conditions, with the DFCs at a final concentration of 0.5 nm. For the DBC_30_, the 30 inner handles were localized on the middle helix circle, whereas for the DSC_30_, the handles were evenly distributed across all hexagon‐hexagon edges (Figure S1, Supporting Information). After incubation periods of 5, 15, 30, 60, and 120 min, the samples were analyzed using agarose gel electrophoresis (AGE), the unbound Cy5‐a′‐Apt at each time point was quantified and normalized against a 100% input control. As shown in Figure 2b, DBC_30_—characterized by its largest window size—exhibited the fastest capture kinetics. ≈91% of free Cy5‐a’‐Apt was bound within the first 5 min, reaching a plateau of 93% thereafter. This rapid performance is likely attributed to the barrel's large axial openings, which enable unhindered molecular access and efficient interaction with internal capture sites. In contrast, the DSC_30_, with a greater number of windows but 7–10 times smaller aperture areas, showed the slowest capture rate, consuming only 83% of free targets within the first 5 min and gradually increasing to 87% over 2 h. Interestingly, despite having the smallest windows, the DIC_30_ exhibited slightly faster capture kinetics (86%∼90%) than DSC_30_. This suggests that window size alone does not fully dictate performance; other factors such as smaller particle size, higher translational diffusion rate, more stable aperture geometry, and denser packing of internal handles likely contribute to improved target accessibility in the DIC. Given the complexity of structure‐dependent molecular transport within these nanoscale geometries, future efforts involving computational modeling or molecular dynamics simulations may help elucidate the underlying mechanisms. Nevertheless, our findings underscore that rational geometry and internal design remain pivotal in regulating molecular entry into DFCs, with the DBC demonstrating exceptional efficiency in rapidly capturing target molecules. Remarkably, DBC accomplished the target molecule removal within 15 min, a timeframe comparable to the DFCs’ half‐life in mouse vasculature.^[^ ^14b^ ^]^ To further assess the aptamer loading efficiency of DFCs under varying internal modification densities, we conducted a parallel comparison of multiple structures bearing 0, 30, 90, or 150 inner handles and incubated them with a 1:1 molar ratio of Cy5‐a′‐Apt to handle for 2 h. AGE analysis (Figure S4, Supporting Information) revealed that DBC structures consistently achieved the most efficient aptamer loading, as evidenced by the lowest levels of unbound aptamer in the supernatant. The slight rise in unbound aptamer with increased loading density may reflect steric hindrance or crowding effects within the inner cavity, but overall, DBC structures offered superior labeling efficiency and molecular carrying capacity across all tested configurations.

Next, to verify the TGFβ1‐capturing function of aptamer‐equipped DFCs, we incubated DBC_30_‐A, DSC_30_‐A, and DIC_30_‐A with TGFβ1 for 15 min and analyzed the samples by AGE (Figure 2c). However, no clear differences in band mobility were discernible across the samples. Therefore, we performed MST using Cy5‐a’‐Apt functionalized DFC_30_ structures before and after TGFβ1 incubation to achieve a more sensitive and quantitative assessment. All three DFC‐A samples showed distinct thermophoretic mobility changes upon TGFβ1 binding, confirming successful protein capture. The magnitude of mobility shift followed the trend DBC_30_‐T > DSC_30_‐T > DIC_30_‐T (Figure S5, Supporting Information), correlating well with differences in structural accessibility and volume. To further validate these findings, negatively stained Transmission Electron Microscopy (ns‐TEM) was employed for structural characterization (Figure 2d). In the TEM images of the DBC_30_‐T sample, a distinctive layer of “fluffy” material was clearly visible along the inner walls of the barrels, indicating TGFβ1 attachment. In contrast, the control DBC30‐A sample without TGFβ1 retained sharp and smooth edges. For DSC_30_, minimal morphological changes were observed with or without TGFβ1, aligning with its weaker oligonucleotide capture. Meanwhile, the DIC_30_ sample revealed dense electron‐rich regions confined within the interior. Together, these analyses confirmed the effective and structure‐dependent protein‐capturing capability of the aptamer‐functionalized DFCs under short‐term incubation conditions.

To explore the full capturing capacity of DFCs, DBC, and DSC structures carrying increased aptamer densities were incubated with TGFβ1 at a 1:1 aptamer‐to‐protein ratio and analyzed across various timepoints (15, 30, 45, and 60 min). As shown in the gel images in Figure S6 (Supporting Information), DBC_30_‐T maintained a single, sharp band with consistent brightness and mobility throughout all time points. However, DBC_90_‐T and DBC_150_‐T bands, while intact at 15 min, showed significant smearing at 30 min and eventually shifted to the loading wells after 45 and 60 min of incubation. Corresponding nsTEM images for DBC_150_‐T revealed effective TGFβ1 sequestration within 15 min, evidenced by a denser protein layer along the barrel's inner wall compared to DBC_30_‐T. At later time points, pronounced aggregation of barrels was observed, correlating with the band retardation in the gel. Alongside aggregation, partial deformation of the barrels was evident, with some cylindrical structures appearing squashed, likely resulting from localized clustering of TGFβ1 molecules within the barrels. This deformation and barrel stacking were further attributed to the intrinsic propensity of TGFβ1 to aggregate,^[^ ^1^, ^22^ ^]^ as well as the exposure of trapped TGFβ1 on outer layers, which mediated inter‐barrel collisions. Similarly, DSC_90_‐T, with its maximum aptamer capacity, showed significant aggregation and structural collapse attributed to structural softness, as evidenced by AGE and nsTEM (Figure S7, Supporting Information). Yet, DIC_30_‐T maintained rigidity and monodispersity with proteins clearly confined within its geometry throughout the experiment.

The in vitro TGFβ1 sequestration performance of the DFCs demonstrates their capacity to effectively perform molecular capturing within a limited time frame, laying a solid foundation for subsequent evaluations at cellular and animal levels.

### 2.2 Cellular Modulation upon DFC‐Mediated TGFβ1 Capturing

**Figure 3:**
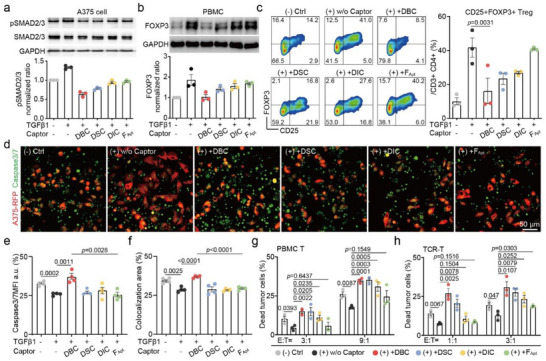
Cellular Modulation upon DFC‐mediated TGFβ1 Capturing a,b). Western blotting analysis of (a) the phosphorylated SMAD2/3 and total SMAD2/3 protein expression in A375 tumor cells that were incubated with three DFCs or F_apt_ plus supplemented TGFβ1 for 4 h; and (b) FOXP3 expression in PBMC that were treated for 24 h c). Representative flow cytometry plots and statistical analysis of the Treg cell populations in PBMC d). Representative confocal microscopy images of PBMC killing assay toward A375‐RFP tumor cells at the effect to target ratio of 9:1 in different treatments. The live A375‐RFP cells show red fluorescence, and the apoptotic cells stained with FITC‐caspase 3/7 antibody show green color. Scale bar = 50 µm. e,f). Statistical results of (e) caspase 3/7 mean fluorescent intensity (MFI), and (f) A375‐RFP and caspase3/7 colocalization area percentage of each sample, quantified from four distinctive images g,h). Flow cytometry statistical analysis of the CFSE+PI+ cell percentage in g) PBMC tumor cell killing assay, and h) TCR‐T specific tumor cell killing assay. In sample labels, (+) or (‐) reflects if additional TGFβ1 was supplemented. Data are presented as mean ± s.d. from n independent experiments (a–c and g‐h, n =  3; d‐f, n =  4). Statistical significance was analyzed by one‐way ANOVA with Tukey's multiple comparisons test. To ensure figure clarity, the specific *p*‐values indicating the significance of group differences in panels a–c, and e–h are summarized in Table S5 (Supporting Information).

To comprehensively investigate the effects of DFC‐mediated TGFβ1 sequestration on tumor immunity, we examined its impact on tumor cell growth, immune cell differentiation, and immune cytotoxicity in co‐culture, step by step. In order to employ a suitable cell model, we first compared the TGFβ1 secretion levels in the culture supernatants of six commonly used tumor cell lines. Among them, A375 human melanoma cells exhibited the highest secretion, reaching ≈10 pM (1 million cells in a 400 uL system, 24‐h culture), as shown in Figure S8 (Supporting Information). This significant TGFβ1 expression and secretion made A375 an ideal model cell line for downstream evaluations. To better evaluate the effects of DFC‐induced TGFβ1 neutralization, the culture medium was supplemented with TGFβ1 to a final concentration of 50 pM. After a 4 h culturing, western blot analysis of SMAD2/3 phosphorylation (pSMAD2/3) levels—a downstream marker of TGFβ1 signaling—revealed that exogenous TGFβ1 addition increased pSMAD2/3 by ≈36.7% compared to untreated cells (**Figure** **3**a). Introduction of DFCs loaded with the full capacity of anti‐TGFβ1 aptamers—DBC_150_, DSC_90_, and DIC_30_— was evaluated for their ability to suppress pSMAD2/3 levels. The samples were adjusted to ensure an efficient final aptamer concentration of 1 nm. Free aptamers (F_Apt_) and DIC effectively reduced pSMAD2/3 levels back to the baseline observed in the negative control group, neutralizing the effects of exogenous TGFβ1. In contrast, DSC and DBC further suppressed pSMAD2/3 levels to 77.2% and 63.3% of the control group, respectively. These results demonstrate that the structural organization of aptamer molecules within different DFCs led to varying TGFβ1‐blocking efficiencies. While all DFCs achieved superior inhibition by physically sequestering TGFβ1, DBC exhibited the most potent suppression due to its rapid and efficient molecular capturing capability. Over a time course, treatment of A375 cells with DBC for 14 h revealed a rapid decrease in pSMAD2/3 levels, reaching a minimum at 4 h, sustained at a plateau until 8 h, and gradually returning to normal (Figure S9, Supporting Information).

To investigate the impact of TGFβ1 removal on immune cells, human peripheral blood mononuclear cells (PBMCs) were treated with additional TGFβ1 (reaching 50 pM), as the baseline concentration in normal PBMC culture is extremely low, necessitating supplementation to evaluate the subsequent capturing effect. Following this, capturing agents were introduced similarly as in the A375 tumor cell system (see Experimental Section). The expression of FOXP3, a key biomarker for regulatory T cells (Tregs), was analyzed via Western blot (Figure 3b). Compared to untreated PBMCs, the addition of TGFβ1 resulted in a 1.9‐fold increase in FOXP3 expression, confirming its key role in driving Treg differentiation. Remarkably, the DBC‐treated group successfully maintained FOXP3 expression at levels comparable to the control group, indicating effective inhibition of TGFβ1‐induced Treg differentiation. While DSC and DIC also reduced FOXP3 expression, their effects were less pronounced. In contrast, treatment with free aptamer failed to downregulate FOXP3 expression.

We further analyzed the proportions of various T‐cell subsets in treated PBMCs using flow cytometry. Specifically, Tregs were identified as CD3+CD4+ T cells co‐expressing CD25 and FOXP3.^[^ ^23^ ^]^ As shown in Figure 3c, the addition of TGFβ1 significantly increased the proportion of Tregs, aligning with its role in promoting Th0 differentiation into Tregs.^[^ ^24^ ^]^ Upon the removal of TGFβ1 by DFCs, the Treg proportion decreased across all treated groups. Notably, cells treated with DBC exhibited the lowest Treg percentage, comparable to the control group without exogenous TGFβ1. Consistent with the Western blot results, free aptamer treatment, lacking the structural framework of DFCs, failed to reduce Treg levels. In addition to modulating Treg populations, flow cytometric analysis revealed a significant increase in effector T cell populations (CD3+CD45RA+CD62L‐).^[^ ^25^ ^]^ in PBMCs treated with DBC (Figure S10, Supporting Information). These findings highlight the dual regulatory effects of DBC on immune cells, reducing Treg differentiation while enhancing effector T cell populations. Such effects are attributed to the molecular capturing mechanism of DFCs, where aptamer‐functionalized structures act as robust physical barriers, effectively sequestering TGFβ1 molecules and reducing their interaction with cell surface receptors. To further validate this, we constructed a DNA soccer‐ball outer captor (DSOC), designed to present 90 aptamers on its external surface. Compared to DSC, DSOC exhibited a pronounced tendency to aggregate following the extension of surface handles and subsequent aptamer modifications. Interestingly, upon capturing TGFβ1, DSOC_90_‐T showed rescued monodispersity, evidenced by sharper and faster migration in AGE analysis and a shrunken morphology in TEM images, indicative of successful protein capture that induced localized aggregation. However, flow cytometric analysis of PBMC co‐culture revealed that DSOC's performance in inhibiting Treg differentiation and enhancing effector T cell populations was notably inferior to DSC (Figure S11, Supporting Information), highly supporting the necessity of a confined space for effective TGFβ1 isolation.

Building on the functional impact of TGFβ1 elimination by DFCs on both tumor cells and T cell subset differentiation, tumor cytotoxicity was examined in an allogenic killing assay by mixing PBMCs and A375 tumor cells at a 9:1 ratio. To assess whether TGFβ1 scavenging could enhance general immune‐mediated cytotoxicity, we employed this PBMC–tumor co‐culture model as a physiologically relevant in vitro system comprising multiple nonspecific effector subsets‐ including monocytes, dendritic cells, and NK cells‐ whose functions are also known to be suppressed by TGFβ1 in the tumor microenvironment.^[^ ^1^, ^2^ ^]^ Under exogenous TGFβ1 treatment and subsequent capture by DFCs, the co‐culture was incubated for 10 h, with apoptotic cells stained using a caspase‐3/7 detection kit and visualized via confocal microscopy. The DBC‐treated group exhibited a markedly higher proportion of green fluorescent apoptotic cells with reduced size (Figure 3d), indicating significant tumor cell apoptosis, while other groups showed minimal effects. Quantitative analysis of multiple fields (Figure S12, Supporting Information) revealed that the DBC‐treated group achieved a 28.2%–41.5% increase in tumor cell killing, as assessed by the mean fluorescence intensity (MFI) of caspase‐3/7 signal (Figure 3e) and the area percentage of apoptotic A375 cells (colocalization of A375‐RFP red fluorescence with caspase‐3/7 green fluorescence, Figure 3f). Using flow cytometry to quantify tumor cytotoxicity, tumor cells pre‐labeled with CFSE were analyzed for the proportion of dead cells (CFSE+PI+) (Figure 3g). At effector‐to‐target (E: T) ratios of 3:1 and 9:1 with PBMCs and A375, DFCs significantly restored the killing efficiency suppressed by TGFβ1, whereas the F_Apt_ group showed minimal improvement. Together with the imaging results, these findings underscore the ability of DBC to broadly restore suppressed immune cytotoxicity in a nonspecific co‐culture system upon TGFβ1 sequestration.

The impact of TGFβ1 regulation was further assessed in a tumor antigen‐specific killing system, utilizing PBMC T cells engineered to express a TCR recognizing A375 antigen, NY‐ESO‐1 (TCR‐T cells), to co‐culture with A375. At E: T ratio of 1:1 or 3:1, the addition of TGFβ1 showed significant suppression of TCR‐T killing to A375 tumor cells. In contrast, the DBC group could not only achieve superior immune recovery from the supplemented TGFβ1 but also further enhance tumor killing by neutralizing TGFβ1 released by A375 (Figure 3h).

Taken together, the TGFβ1 scavenger DFCs demonstrated a potent ability in regulating the functional impact of TGFβ1 on tumor cells, immune cells, and the co‐culture killing system. Among the tested constructs, DBC demonstrated the most efficient performance due to its largest window aperture, fastest capture rate, and highest density of aptamer modifications. Remarkably, the leveraging effect—where pM concentrations of input structures yield disproportionately significant regulatory outcomes—underscores its potential in cancer immunotherapy.

### 2.3 In Vivo Validation of DFC Therapeutic Efficacy

Before conducting animal studies, we first assessed the serum stability of F_Apt_ and 30‐aptamer‐conjugated DFCs by incubation in 80% serum at 37 °C. As shown in Figure S13 (Supporting Information), F_Apt_ began to degrade from 0.5 h, with DSC_30_‐A degrading fastest among the DFCs, DBC_30_‐A showing moderate stability, and DIC remaining largely intact up to 4 h. Reviewing our previous pharmacokinetic studies,^[^ ^14b^ ^]^ radiolabeled DFCs and DNA oligos exhibited circulation half‐lives of less than 10 min, followed by predominant hepatic and renal accumulation and clearance—well before significant structural degradation occurred. These observations suggest that the intended therapeutic effect of this strategy must take place within this brief circulation window, rather than relying on long‐term structural stability.

We further investigated the anti‐tumor efficacy of DFCs in an established mouse tumor model. In addition to efficient TGFβ1 neutralization demonstrated in the in vitro system, effective tumor control in vivo also depends on the DFCs’ efficient capturing of the cytokine within the dynamic and complex bloodstream, as well as on whether TGFβ1 clearance in peripheral blood can significantly modulate the TME. In an MC38 subcutaneous mouse model, 0.8 pmol DBC_150_‐A (4nm × 200 µL) equipping 120 pmol of aptamers was administered via tail vein injection on day 8 post‐tumor inoculation, when tumor volumes reached ∼50 mm^3^. Blood samples (150 µL) were collected from the retro‐orbital venous plexus at indicated time points, and TGFβ1 levels in serum were quantified using ELISA (see Figure S14, Supporting Information). Within 4 h of DBC injection, circulating TGFβ1 levels dropped from 4.8 ng mL^−1^ (199.2 pM) to ≈50%, followed by a gradual return to baseline over the next 8 h. As the ELISA antibody used recognizes both active and latent TGFβ1, the assay reflects overall TGFβ1 levels; however, given the aptamer's significantly higher affinity for the active form (*Kd* = 28 nm vs 152 nm, Figure S3, Supporting Information), it is likely that active TGFβ1 was preferentially captured during this clearance process. These results confirmed the ability of DBC to effectively capture and clear TGFβ1 in the periphery. Moreover, the dynamic clearance‐recovery pattern of TGFβ1 highlighted the existence of a cytokine equilibrium between the tumor and bloodstream in tumor‐bearing mice.

**Figure 4:**
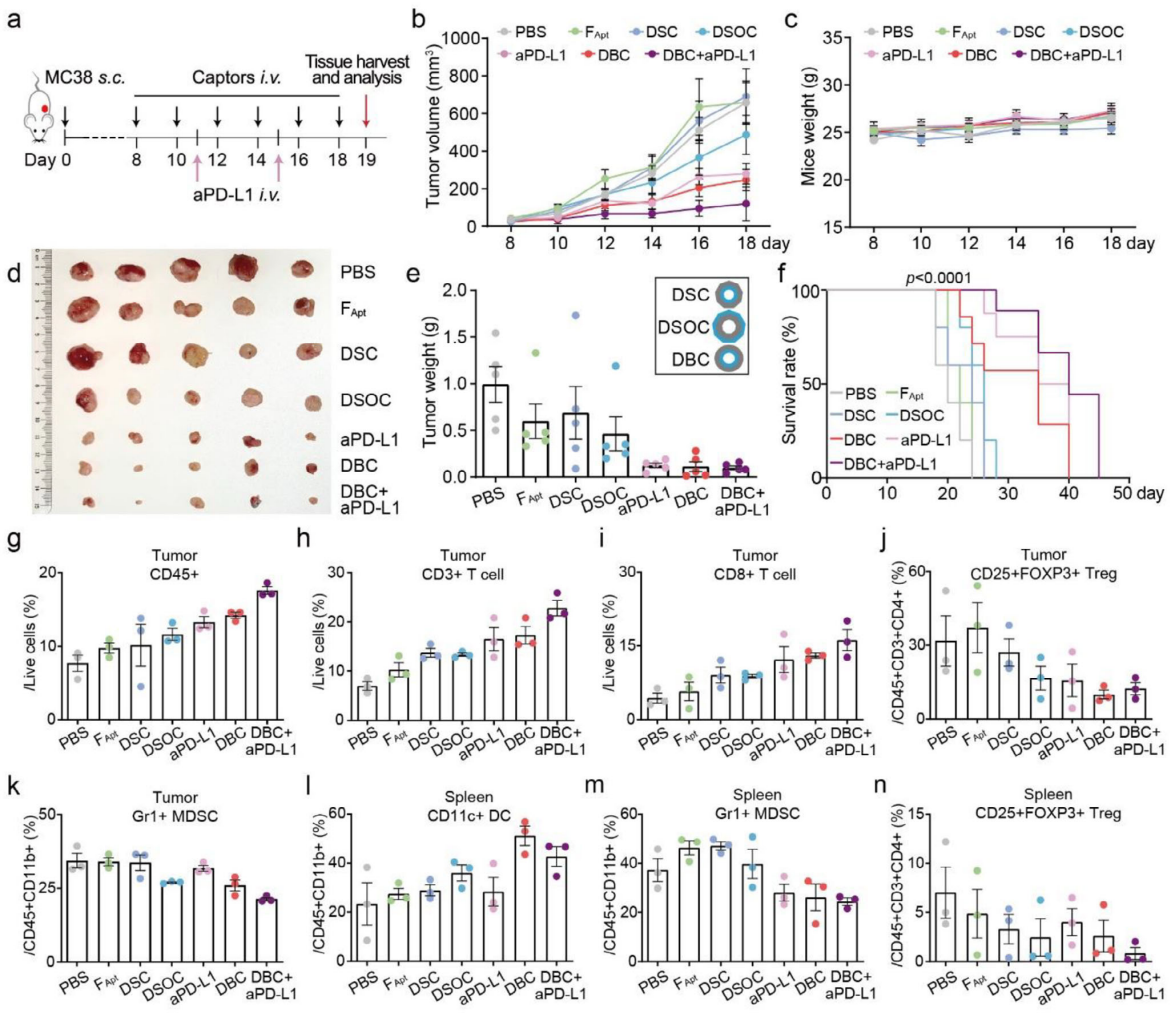
In Vivo Validation of DFC Therapeutic Efficacy a). Schematic illustration of the timeline for MC38 tumor inoculation and drug administrations in a C57BL/6 mouse model b). Average tumor growth kinetics after administration c). Mouse body weight monitoring d). Picture of *Ex vivo* tumors from the mice with different treatments e). Ex vivo tumor weight measurements f). Survival curve of MC38 tumor‐bearing mice in each group g–k). Flow cytometry (FC) analysis of the population of specific cell types in the tumor l–n). FC analysis of specific cell types in the spleen. Data are presented as mean ± s.d. The MC38 tumor‐bearing mouse experiments were repeated three times independently, and the data presented in this figure are from the final experiment. Statistical significance is analyzed by one‐way ANOVA with Tukey's multiple comparisons test for b, e, and g‐n; and log‐rank (Mantel–Cox) test for f. To ensure figure clarity, the specific *p*‐values indicating the significance of group differences in panels b, c, e, and g‐n are summarized in Table S5 (Supporting Information).

Based on the rapid clearance kinetics of DFCs in the bloodstream, we employed a multiple‐dosing strategy to remodel the immune environment. In addition to the PBS group as a negative control, we included groups treated with F_Apt_ (free aptamer), DSC (90 inner aptamers), and DSOC (90 outer aptamers), with aptamer dosage normalized (300 pmol/shot) for comparison with the DBC group. These treatments were administered every 48 h for a total of six doses. Furthermore, based on literature demonstrating the synergistic anti‐tumor effect of dual blockade of TGFβ1 and PD‐L1 signaling,^[^ ^5^, ^7^, ^8^ ^]^ we included a group treated with a combination of DBC and PD‐L1 antibodies (aPD‐L1), as well as aPD‐L1 alone as a control. Compared to F_Apt_ and other DFC structures, DBC induced better tumor suppression, consistent with findings from the in vitro systems (**Figure** **4**b). Moreover, its combination with aPD‐L1 further enhanced the anti‐tumor efficacy of DBC. Interestingly, although showing limited tumor suppression, DSOC demonstrated relatively greater efficacy compared to DSC, suggesting that its external aptamer modifications facilitated faster TGFβ1 capture and that the DNA framework promoted rapid clearance. However, its performance was still inferior to that of DBC, indicating that while surface accessibility is important for efficient cytokine binding, the added spatial confinement and optimized geometry of DBC further enhance sequestration—highlighting the importance of balancing accessibility and entrapment in DNA framework design.

Notably, no significant differences in body weight (Figure 4c) or other physiological and behavioral abnormalities were observed among the groups during the treatment period. Tumors dissected on day 19 after tumor inoculation showed consistent trends in both morphological observations (Figure 4d) and weight analyses (Figure 4e). Survival analysis of the remaining five mice in each group revealed that the combination therapy group achieved an 80% survival rate within 30 days (Figure 4f), highlighting the synergistic benefits of combining DBC with aPD‐L1 in tumor suppression.

To investigate the effects of treatment on both the TME and systemic immunity, we performed flow cytometric analysis on tumor tissues and spleens from mice in each treatment group. In the tumor tissues, the number of total CD45+ immune cells as well as T cell subsets including CD3+ T and CD8+ T cells was significantly increased in the DBC, aPD‐L1, and combination therapy groups, with the combination therapy group showing the most pronounced elevation (Figure 4g–I, and gating details in Figure S15, Supporting Information), whereas the CD4+ T cell subset yielded similar elevation via the single or combo treatment (Figure S16, Supporting Information). Importantly, the proportion of immunosuppressive Treg cells was significantly reduced in the DBC‐included groups compared to the aPD‐L1 only group (Figure 4j), echoing the in vitro results of PBMC treatment. Furthermore, we examined myeloid cell infiltration within the tumor, revealing a modest increase in dendritic cells (DCs) in the combination treatment group (Figure S16, Supporting Information). Notably, the reduction in the myeloid‐derived suppressor cell (Gr1+ MDSC) population in the DBC, DSOC, and combination therapy groups, relative to the aPD‐L1 only group (Figure 4k, and gating details in Figure S17, Supporting Information). Together, it suggests that our TGFβ1 captors contribute to the downregulation of immunosuppressive cells (Treg and MDSC) and enhancement of effector T cells in the TME, thereby promoting the efficacy of tumor immunotherapy.

Moreover, analysis of splenic immune cell populations provides insights into the potential influence on systemic immunity. Flow cytometric analysis of splenic cells after treatment revealed notable shifts in key immune subsets. Among CD11b+ myeloid cells, DBC treatment significantly increased the proportion of dendritic cells (DCs) (Figure 4l). Both the DBC monotherapy and combination therapy groups effectively reduced the proportion of immunosuppressive Gr1+ MDSCs (Figure 4m). Additionally, the combination therapy group markedly decreased the proportion of Treg cells (Figure 4n), with gating details provided in Figure S18 (Supporting Information). Meanwhile, only minimal increases in CD3+ T cells were observed in the DBC group, with no significant changes in CD4+ or CD8+ T cells (Figure S19, Supporting Information). These findings highlight the ability of DBCs to antagonize the immunosuppression mediated by Treg and MDSC populations in the periphery, while promoting antigen‐presenting cells with minimal interference in the T cell compartment. This is consistent with the systemic immune‐modulating effects of TGFβ1 in tumor‐bearing mice.

**Figure 5:**
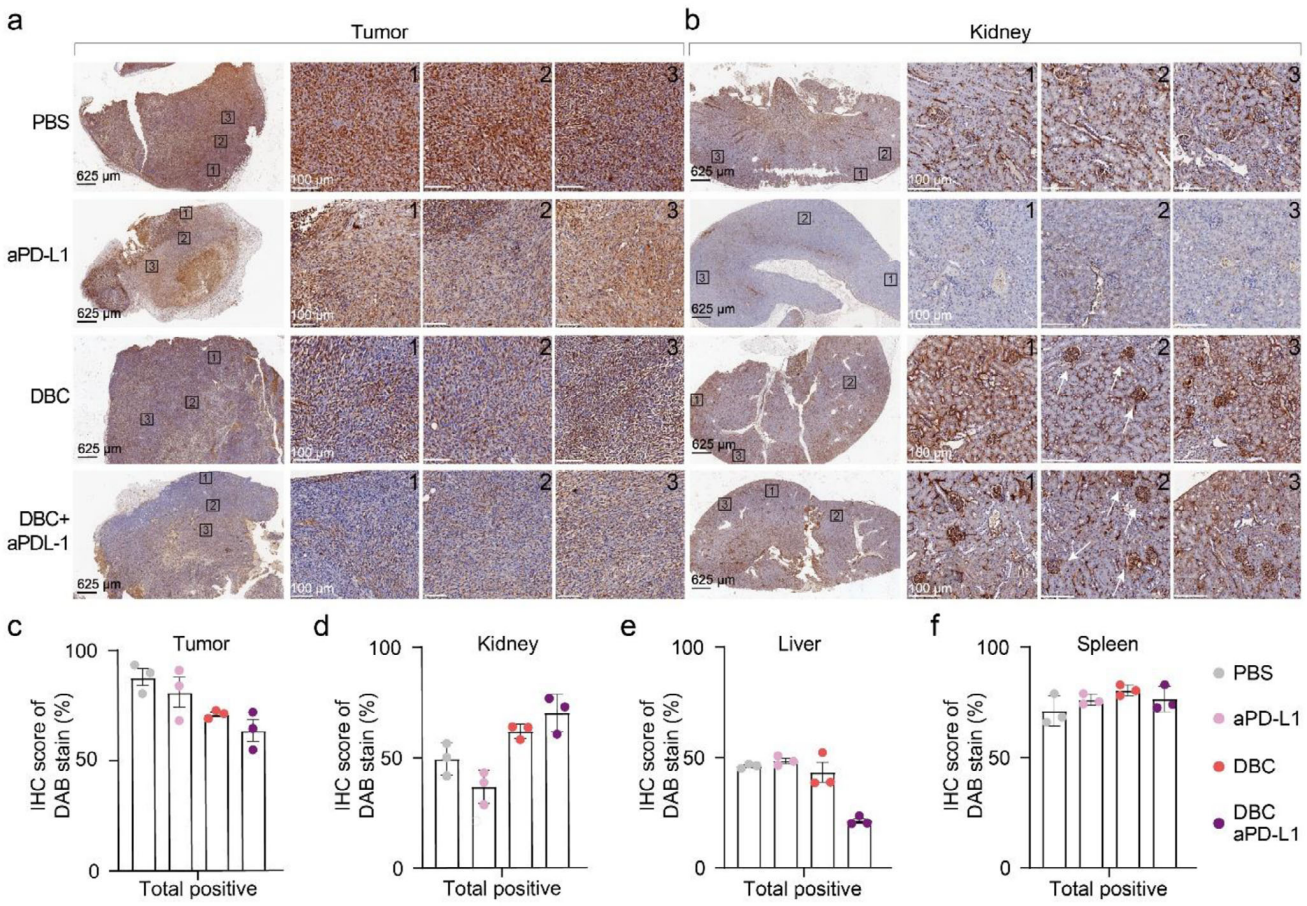
TGFβ1 Immunohistochemical Analysis a,b). Representative TGFβ1 IHC images of tumor (a) and kidney (b) tissues for each group. Black boxes indicate the exact locations of the enlarged image on the right. White arrows in the middle zoomed‐in images in (b) point to the renal glomeruli c–f), Statistical results of the total positive staining percentage of TGFβ1 (IHC score) in tumor(c), kidney(d), liver(e), and spleen(f) tissue IHC images analyzed through the IHC profiler plugin in ImageJ software. Data are presented as mean ± s.d. Each IHC condition was performed with 2–3 biological replicates. For each sample, three distinct regions (boxes 1–3) were selected for quantification to account for intra‐sample variability. The specific *p*‐values are summarized in Table S5 (Supporting Information).

To further confirm that the therapeutic effects of DBC stem from its TGFβ1 capture and to determine its final destination, we performed anti‐TGFβ immunohistochemical (IHC) staining (by DAB staining as shown in brown coloration) on tumors and key organs, including the kidney, liver, and spleen. IHC enables immediate fixation of excised tissues, thereby minimizing post‐excision degradation and better preserving the native distribution of soluble proteins such as TGFβ1. Moreover, direct IHC staining of TGFβ1 provides the most intuitive visualization of its spatial distribution across tissues, offering clearer insight into DFC‐mediated cytokine sequestration in vivo. Since organ fixation was conducted 12 h after the final injection, and given that DFCs were shown to degrade within this time window under physiological conditions (Figure S14, Supporting Information), we interpret the IHC‐detected TGFβ1 signals as primarily representing free or released protein, rather than aptamer‐bound forms. The IHC images revealed significant differences in TGFβ1 distribution across the tumor (**Figure** **5**a) and kidney (Figure 5b) under different treatments. Quantitative analysis is presented in histograms (Figure 5c–f), with processing methods detailed in Figure S20 (Supporting Information).

In tumors, the PBS group exhibited the highest TGFβ‐positive staining since tumor size intrinsically affects TGFβ1 secretion levels. Compared with the PBS or aPD‐L1 groups, the tumors in DBC or combination therapy groups showed a noticeable reduction in TGFβ staining (Figure 5c), even by visual inspection. This provides direct evidence that systemic TGFβ1 sequestration can effectively influence its abundance in the TME. Conversely, the trend in the kidney was the opposite. The aPD‐L1 monotherapy group exhibited significantly lower TGFβ1 levels in the kidney compared to the PBS group (37.0% vs 49.5%), reflecting the correlation between tumor burden and TGFβ1 level in circulation. In contrast, the kidneys in the DBC and combination therapy groups displayed higher TGFβ1 accumulation than the PBS group (Figure 5d). Substantial TGFβ1 was visualized aggregating in vascular endothelial cells and renal glomeruli (Figure 5b). This suggested that TGFβ1 captured by DBC is retained in the kidney, consistent with the known renal tropism of DNA origami frameworks. While this raises concerns about potential kidney toxicity, pathological analysis indicated the absence of notable abnormalities in the kidneys of DBC‐treated mice at the current dosage. Although previous studies have indicated that nucleic acid nanostructures accumulating in the kidney may help mitigate acute kidney injury by scavenging reactive oxygen species,^[^ ^14a^ ^]^ it remains critical to investigate whether renal TGFβ1 accumulation could lead to adverse effects. To address this, we performed histological analysis of kidney tissues collected 8 h after treatment. H&E staining revealed no visible pathological changes in any treatment group, including DBC and DBC+aPDL1 (Figure S21, Supporting Information). In parallel, serum biochemical analysis showed that creatinine and urea levels remained within the normal physiological range (Table S8, Supporting Information). These results indicate that the DFC treatments did not induce apparent renal toxicity within the therapeutic window.

IHC images for the liver and spleen are shown in Supplementary Figure S22 (Supporting Information). Although the liver is another primary organ for DBC retention and metabolism,^[^ ^14b^ ^]^ the groups treated with DBC did not show increased TGFβ1 levels in the liver. Interestingly, the combination therapy group exhibited significantly reduced TGFβ1 levels in the liver compared to other groups (Figure 5e), potentially due to the liver's robust metabolic capacity and a yet‐to‐be‐elucidated interplay between aPD‐L1 and DBC treatment. Supporting this observation, serum biochemical analysis (Table S8, Supporting Information) revealed noticeable elevation in serum ammonia levels in the DBC+aPDL1 group of tumor‐bearing mice, which may reflect increased hepatic nitrogen metabolism following enhanced cytokine clearance. Notably, other hepatic and renal function markers—including uric acid, alanine aminotransferase (ALT), aspartate transaminase (AST), and creatinine—remained within the normal physiological range, suggesting that the combination treatment induced no detectable hepatic or renal toxicity. As for the spleen, a non‐accumulative organ for DFCs,^[^ ^14b^ ^]^TGFβ levels did not vary significantly across all groups (Figure 5f), likely due to its rapid systemic balancing by 12 h post‐DBC administration, consistent with the observed dynamics of circulating TGFβ1 levels (Figure S14, Supporting Information). To further support the findings, an additional animal per group was examined by IHC for the major organs, with representative images and TGFβ1 quantification results shown in Figure S23 (Supporting Information).

To assess potential cardiac effects associated with DFC‐based cytokine clearance, H&E staining was applied on heart tissues from the therapeutic experiment. No signs of necrosis, inflammatory infiltration, hemorrhage, or fibrotic remodeling were observed in myocardial tissue across all treatment groups (Figure S21, Supporting Information). In a parallel safety evaluation, mice were sacrificed 8 h after a single dose of intravenous DBC_150_‐A, intraperitoneal aPD‐L1, and their combination to simulate the treatment regimen. Histological examination revealed intact architecture of both cardiac muscle and major vessels, with no detectable pathological alterations (Figure S24, Supporting Information).

Taken together, these findings highlight the importance of organ‐specific accumulation and clearance dynamics in interpreting local TGFβ1 distribution. In our earlier biodistribution assessments, DNA framework structures demonstrated negligible retention in subcutaneous tumors following intravenous administration, which informed our shift toward systemic cytokine clearance rather than tumor‐targeted delivery. As such, the observed decrease of TGFβ1 levels in tumors likely reflects the redistribution effect of DFC‐mediated systemic scavenging. In contrast, the kidney—a major site for DFC accumulation—exhibited prominent TGFβ1 retention, further supporting the mechanism of peripheral clearance and sequestration via circulatory elimination.

## 3 Discussion

In this study, we harnessed DNA origami technology to develop a series of DNA framework captors (DFCs) designed to organize capturing agents, specifically anti‐TGFβ1 aptamers, for the efficient sequestration and removal of TGFβ1 molecules in cell culture and systemic circulation in mice. By exploring how geometric shapes and pore sizes influence capture efficiency, we identified the barrel‐shaped framework (DBC) as the most effective design, owing to its large entrance and high density of functionalization sites. The structural advantages of DBCs translated into robust TGFβ1 sequestration, achieving potent regulation of tumor cell signaling as well as the differentiation and cytotoxicity of immune cells in vitro. When functionalized with 150 anti‐TGFβ1 aptamers and administered via tail‐vein injection, DBCs demonstrated significant therapeutic efficacy as a single agent, as well as a synergistic effect when used in combination with anti‐PD‐L1 antibodies. Comparative analyses highlighted that, beyond the rapid metabolic clearance inherent to nucleic acid‐based constructs, the superior capture efficiency (DBC vs DSC and DIC) and enhanced spatial sequestration capacity (DBC vs F_Apt_ and DSOC) were critical for DBC's effectiveness in both cell culture and animal models. These findings underscore the potential of DBCs as a versatile platform for precise immunomodulation and inform the rational design of future DNA‐based therapeutic systems.

From a structural perspective, while 2D DNA origami structures provide flat, curvature‐free surfaces ideal for precise control over ligand density and spatial arrangement—facilitating mechanistic studies of receptor engagement^[^ ^11a^ ^]^ and enabling in vivo modulation strategies^[^ ^13d^ ^]^—they primarily operate via surface interactions without enclosed volume and may be more prone to morphological and surface property changes upon protein binding. In contrast, 3D frameworks such as DBC capitalize on internal spatial confinement to shield bound cytokines from receptor access while maintaining surface features conducive to in vivo clearance. This study highlights the complementary advantages of 2D and 3D DNA nanostructures and emphasizes the less explored potential of internal cavity design in 3D frameworks for functional molecular sequestration.

TGFβ1 serves as an ideal model cytokine to prove the potential of DFCs in immunoregulation. It plays a critical role in regulating both tumor cell growth and immune responses, with concentrations at hundreds of pM under physiological conditions, and increasing by less than an order of magnitude in the tumor settings.^[^ ^26^ ^]^ This concentration range aligns well with the functional capabilities of DNA origami‐based molecular tools (with nm devices equipping up to µm agents in sub‐mL scale). From this perspective, a promising application of our system is the control of cytokine storm triggered by pathogen invasion or drug treatment (especially certain immunotherapies). It can cause severe and potentially fatal damage to tissues and organs within 10–12 h, therefore, it demands highly time‐sensitive therapeutic interventions. Notably, the primary cytokines involved in cytokine storms, such as IL‐6, IL‐1β, and TNF‐α are present at concentrations below 100 pM (summarized in Table S6, Supporting Information). Leveraging our clearance strategy to rapidly reduce the levels of these pro‐inflammatory cytokines in the bloodstream could provide a novel approach for preventing or mitigating cytokine storms. Moving forward, we are eager to explore this avenue further and conduct experiments in relevant animal models. Nevertheless, for removing other “trash” molecules with a concentration exceeding 100 nm, the application of DNA origami captor faces challenges, such as increased production costs, preparation complexity, and the toxicity risk at elevated concentrations.

With the continuous evolution of cancer treatment strategies, interventions targeting interactions between immune and tumor cells and normalizing tumor immunity have been proven effective^64^. From a mechanistic standpoint, traditional approaches such as small‐molecule inhibitors and antibody drugs typically rely on stoichiometric inhibition and therefore require equimolar or excess dosing (Table S1, Supporting Information)—with antibodies administered at 100–500 µg per mouse and small molecules often exceeding 1–7.5 mg per mouse. This often imposes metabolic burdens and leads to associated side effects. While the prolonged circulation times of these agents can enhance therapeutic efficacy, they are also a primary contributor to cumulative toxicity. In contrast, our DFC platform offers a distinct approach by using low‐dose (pmol‐level) nano‐containers (Table S4, Supporting Information) to efficiently collect and eliminate substantial amounts of TGFβ molecules along the circulatory route, leading to potent tumor suppression and enhanced therapeutic efficacy. As a distinctive approach for cancer immune modulation, the DFC system opens up exciting new possibilities for the development of innovative therapeutic interventions. Beyond its dosage advantage, this framework‐based system offers spatially defined, modular, and transient engagement with targets, which collectively point to a promising class of programmable cytokine‐neutralizing therapeutics. As a complementary alternative to conventional receptor blockade, DFC‐enabled scavenging may open new directions in precision immunotherapy.

## 4 Experimental section

### Materials and Regents

Single‐stranded scaffold DNA (p7560) was purchased from Tilibit nanosystems. All of the oligonucleotides were ordered from Sangon Biotech or GeneRay Biotechnology. The staple strands were purified using high affinity purification (HAP) method or the oligonucleotide purification cartridge (OPC) method with a stock concentration of 100 µm. The other oligonucleotides were purified by high‐performance liquid chromatography (HPLC).

Recombinant human TGFβ1 (cat.No. 240‐CF) and human IL‐2 (cat.No.BT002) proteins were purchased from R&D. Recombinant human IFN‐γ was bought from Sino biological (catalog No.11725 HNAS). T cell activation antibodies, anti‐CD3 (catalog No.317325), anti‐CD28 (catalog No.302933) were purchased from Biolegend.

Flow cytometry antibodies: anti‐human CD3‐AF488, anti‐human CD25‐PE, anti‐human CD4‐Percp 5.5, anti‐human CD45RA‐PE‐Cy7, anti‐human CD62L‐APC were purchased from BD Biosciences. Anti‐human FOXP3‐APC, antihuman CD8‐PE were purchased from eBioscience. Anti‐mouse CD4‐AF488, anti‐mouse CD3‐PerCP Cy5.5, anti‐mouse CD45‐PE Cy7, anti‐mouse CD25‐APC, anti‐mouse CD11c‐PE, anti‐mouse CD11b‐APC were purchased from BD Biosciences. Anti‐mouse F4/80‐AF488, anti‐mouse Gr1‐Percp Cy5.5 were purchased from Biolegend.

Tumor cell lines A375 and MC38 were purchased from ATCC (Manassas, VA, USA), A375‐RFP cell was purchased from FuHeng biology. PBMC and TCR‐T cells were acquired from Grit Biotechnology Co. All the cells were cultured with 10% (v/v) fetal bovine serum (FBS; Hyclone) and 1% (v/v) penicillin/streptomycin (Gibco, Invitrogen) at 37 °C in a 5% CO_2_ air incubator.

### Instruments

Thermal annealing and incubation processes were carried out using the thermocycler (Bio‐Rad). Purification of the origami structures was conducted using the ultracentrifuge machine (Beckman & Optima XPN‐90). Gel images were captured by Gel Doc EZ Imager (Bio‐Rad). Concentrations of oligos and DNA assembly products were quantified by measuring absorbance at 260 nm using Nanodrop One (Thermo Fisher Scientific). The affinity between TGFβ1 protein and TGFβ1 aptamer was detected using microscale thermophoresis (MST, Nanotemper technologies & Monolith NT.115 Pico). Confocal experiments were performed using Leica & TCS SP8. Flow cytometry experiments were performed using BD & FACSVerse. Transmission electron microscopy imaging was performed using a Hitachi‐HT7700 microscope operated at 100 kV.

### Self‐Assembly and Purification of DNA Framework Captors (DFC)

DNA Barrel Captor (DBC) was designed using caDNano, while DNA Soccer Captor (DSC) and DNA Icosahedron Captor (DIC) were designed using Tiamat software. The scaffold strand p7560 (10 nm) and the staple strands (sequences in Table S2, Supporting Information) were mixed at molar ratio of 1:10 and applied with thermal annealing programs: 75 °C≈5 min, 65 °C≈15 min, 40 °C≈48 h (DBC), 65 °C≈4 °C/‐1 °C per 15 min (DSC), 65 °C≈4 °C/‐1 °C per 36 min (DIC) in 1 ×TE• 10 mm MgCl_2_ buffer. Assembled DNA origami structures were purified using rate‐zonal ultracentrifugation upon a glycerol gradient. Fractions (200 µL per layer) containing the well‐assembled products were identified from AGE analysis, and the collected products were washed (using1 × TE• 10 mm MgCl_2_ buffer) through Amicon ultra‐0.5 mL 10 kD centrifugal filters (Millipore) to remove glycerol and concentrated. The concentrations of purified DFCs were quantified by measuring their A260 absorbance.

### Detecting the Binding Affinity Between TGFβ1 Protein and Anti‐TGFβ1 Aptamer

Lyophilized powder of the TGFβ1 protein (R&D 240‐B) was reconstituted at 100 µg mL^−1^ (4.15 µm) or in sterile 4 mm HCl. TGFβ1 aptamer was dissolved in deionized water with a stock concentration of 100 µm. Aptamer was pretreated using a quick annealing step (95 °C 5 min, 4 °C 30 min) to form the functional secondary structure. The methods were used to verify their binding affinity: native polyacrylamide gel electrophoresis (N‐PAGE) and microscale thermophoresis (MST).

### Native Polyacrylamide Gel Electrophoresis (N‐PAGE)

The a’‐Apt strand (500 nm) was incubated with the TGFβ1 protein for 2 h at 37 °C and molar ratio of 1:1/1:0.5/1:0.2, and the samples were loaded onto 10% native PAGE, running at 80 V for 90 min, the gel was then stained with Sybr Gold (1×) (Invitrogen, S11494).

### Microscale Thermophoresis (MST)

For the MST binding experiment, mixing 25 µL of 10 nm Cy5 labeled TGFβ1 aptamer (Cy5‐a’‐Apt) with 25 µL of 0.5 µm (12 µg mL^−1^) TGFβ1 protein and incubated at 37 °C for 2 h as the complex sample; while mixing it with 25 µL of dilution buffer for the aptamer‐only sample. Dip four Monolith NT.115 Capillary capillaries into each sample assay, place them in the device tray, and start the measurement. In the same way, the Cy5‐a’‐Apt was incubated with the reference proteins: IFN‐γ and BSA, at the same molar ratio, and monitored via MST.

Binding affinity for TGFβ1 aptamer (Cy5‐a’‐Apt) and TGFβ1 protein or latent TGFβ1 protein: mixing 10 µL of 60 nm Cy5 labeled TGFβ1 aptamer (Cy5‐a’‐Apt) with 10 µL of serial diluted TGFβ1 or latent TGFβ1 to reach the final protein concentration of 45 pM to 1.5 µM, incubating at 37 °C for 2 h and monitoring via MST.

Binding check for DFC_30_‐A and TGFβ1 protein: mixing 25 µL of 1 nm Cy5 labeled DFC_30_‐A with 25 µL of 30 nm TGFβ1 protein and incubated at 37 °C for 10mins as the complex sample; while mixing it with 25 µL of dilution buffer for the DFC_30_‐A only sample, and monitored via MST in the same way.

### Validation of the DFCs’ Capture Efficiency and Kinetics Against Cy5‐a’‐Apt

Three DFCs with 30 inner handles (10 nm, 5 µL) were mixed with Cy5‐labeled a’‐Apt (0.3 µm, 5 µL), aiming at 1:1 capture, and incubated for 5/15/30/60/120 min at 37 °C. The samples were then characterized using agarose gel electrophoresis and nsTEM.

### TGFβ1 Capturing Assay in Test Tube

Three DFCs bearing distinct numbers of aptamers (1.5 nm 10 µL) were incubated with recombinant human TGFβ1 with equivalent aptamer‐protein ratio for 15/30/45/60/120 min at 37 °C. The samples were then characterized using agarose gel electrophoresis and nsTEM.

### Agarose Gel Electrophoresis (AGE)

Samples were loaded into the 1.5% agarose gel containing 0.5 × TBE and 10 mm MgCl_2_ and migrated for 2 h at 70 V on ice. Then, gel was post‐stained using nucleic acid dye Gel Red (Tsingke Biotech). Gel images were captured by the CCD equipped with the Gel Doc EZ Imager (Bio‐Rad). Intensities of the interested bands were quantified using an open‐source software ImageJ.

### Negatively Stained Transmission Electron Microscopy (nsTEM)

All carbon‐coated grids were glow‐discharged to increase their hydrophilicity. A 5 µL sample solution was placed on the grid surface and incubated for 1 min. The excess solution was adsorbed with filter paper. The grid was then washed by 5 µL of 2% uranium acetate and stained with a second drop for 1 min. After removing the excess staining solution, the grid was left to air‐dry and examined under Hitachi‐HT7700 TEM, 100 kV.

### Verifying TGFβ1 Secretion Level in Different Tumor Cell Lines

Six tumor cell lines were respectively seeded in a 10 cm cell dish for overnight growth. On the second day, the cell culture medium was replaced with OPTI‐MEM medium, and then cultured for 24 h. Tumor cell culture supernatant (produced by ≈4 × 10^6^ cells/24 h) was collected and filtered through a 0.2 µm filter (Millipore), preparing for ELISA quantification. The TGFβ1 in the samples was acid‐activated and measured according to the ELISA kit instructions (*Human TGF‐β1 DuoSet ELISA Kit*, R&D Systems, Catalog No. DY240).

### Detecting Phosphorylation Levels of SMAD2/3 Protein in A375 Cells

A375 cells were pre‐seeded in the 24‐well plate for overnight growth. On day 2, all groups except the Ctrl samples were supplemented with extra rhTGFβ1 (1 ng mL^−1^, 0.0415 nm). Meanwhile, cells were treated with DBC_150_‐A (1 nm × 400 µL) / DSC_90_‐A (1.67 nm × 400 µL) / DIC_30_‐A (5 nm × 400 µL) / DSOC_90_‐A (1.67 nm × 400 µL) or free aptamers (150 nm × 400 µL) for 2 h/4 h/8 h/14 h. Then cells were collected for western blotting examination.

### Detecting FOXP3 Expression Level and T Cell Differentiation in PBMC

Human peripheral blood mononuclear cells (PBMC; GRIT Biotech) were seeded in a 48‐well plate. For all groups, cells were supplied with 20 ng mL^−1^ IL2, 5 µg mL^−1^ aCD3, and aCD28. All groups except Ctrl were added with extra rhTGFβ1 (10 ng mL^−1^, 0.415 nm). Meanwhile, cells were treated with DBC_150_‐A (2 nm × 200 µL) / DSC_90_‐A (3.33 nm × 200 µL) / DIC_30_‐A (10nm × 200 µL) / DSOC_90_‐A (3.33 nm × 200 µL) or F_Apt_ (300 nm × 200 µL) for 24 h (for WB) or 48 h (for FC).

### Western Blotting Assay

Cells were lysed in SDS sample buffer with 5 mm DTT plus complete protease and phosphatase inhibitors for 10 min on ice. Lysates were heated to 100 °C, loaded on SDS‐PAGE, and transferred to PVDF membrane (Millipore). Membranes were blocked in 5% BSA in TBST for 1 h, then probed with the specific pSMAD 2/3 antibody (CST #8828), SMAD 2/3 antibody (CST #8685), FOXP3 antibody (CST #5298) and GAPDH antibody (Epizyme Biotech #LF211) overnight at 4 °C, followed by incubation with an anti‐rabbit secondary antibody (1:10000; CST, 7074), and reactive bands visualized using Amersham Imager 600 instrument (GE Healthcare).

### Flow Cytometry Assay for T Cell Differentiation

Cells were collected and washed using PBS buffer. Preparation of two antibody mixing solution panels for cell surface staining: panel 1: aCD3‐AF488, aCD4‐PerCP‐Cy5.5, aCD25‐PE; panel 2: aCD3‐AF488, aCD4‐PerCP‐Cy5.5, aCD8‐PE, CD45RA‐PE‐Cy7, CD62L‐APC. Cells of each group were equally divided and stained, respectively, with each antibody panel. Cells for dying with the first panel were fixed, permeabilized, and then stained with aFOXP3‐APC. Samples were finally analyzed using BD FACSVerse. Raw data were analyzed with Flowjo v10.10.

### Non‐Specific T Cell Killing Assay

PBMC were seeded in a 48‐well plate. For all groups, cells were activated by 5 µg mL^−1^ aCD3, 5 µg mL^−1^ aCD28 antibody, and 20 ng mL^−1^ IL2. All groups except Ctrl were supplied with extra rhTGFβ1 (10 ng mL^−1^, 0.415 nm). Meanwhile, cells were treated with DBC_150_‐A (2 nm × 200 µL) / DSC_90_‐A (3.33 nm × 200 µL) / DIC_30_‐A (10 nm × 200 µL) / DSOC_90_‐A (3.33 nm × 200 µL) or F_Apt_ (300 nm × 200 µL) for 72 h. After that, PBMCs were co‐incubated with A375‐RFP tumor cells / A375‐CFSE tumor cell at the effector: target cell ratio of 3:1 and 9:1 for 10 h (A375‐RFP tumor cells were used for confocal microscopy observation, A375‐CFSE tumor cells were used for flow cytometry). For confocal microscopy observation, co‐cultured cells were stained with caspase‐3/7 detection kit (#I35106, Thermo Fisher scientific) according to the manufacturer's instructions. For the flow cytometry experiment, cells were collected, washed with PBS, and then stained with PI. Cells were analyzed using BD FACSVerse. CFSE+PI+ tumor cells were gated and analyzed in Flowjo v10.10.

### Specific TCR‐T Cell Killing Assay

TCR‐T cells (GRIT Biotech) were seeded in a 48‐well plate. For all groups, cells were activated with 10 ng mL^−1^ IL2. All groups except Ctrl were added with extra rhTGFβ1 (10 ng mL^−1^, 0.415 nm). Meanwhile, cells were treated with DBC_150_‐A (2 nm × 200 µL) / DSC_90_‐A (3.33 nm × 200 µL) / DIC_30_‐A (10 nm × 200 µL) or free aptamers (300 nm × 200 µL) for 72 h. Then, TCR‐T cells were respectively co‐incubated with A375‐CFSE tumor cells at the effector: target cell ratio of 1:1 and 3:1 for 4 h, killing. Cells were collected, washed with PBS, and stained with PI. Then cells were analyzed using BD FACSVerse. CFSE+PI+ tumor cells were gated and analyzed in Flowjo v10.10.

### Circulating TGFβ1 Clearance and Dynamic Recovery Examination

MC38 tumor baring C57BL/6 Mice were intravenously injected with DBC_150_‐A (4 nm × 200 µL), blood samples were collected from mouse retro‐orbital sinus, transferred to ethylenediaminetetraacetic acid (EDTA) coated mini vacutainer tubes (BD‐68784, BD Biosciences), and allowed to clot at 37 °C for 30 min and then 4 °C for 2 h. Serum was isolated from blood after centrifugation at 3000 g, 10 min and prepared for ELISA. The total protein of the samples was quantified by BCA assay, and total protein input was normalized across samples. The TGFβ1 in the samples was acid‐activated and measured following the ELISA kit instructions (*Mouse TGF‐β1 DuoSet ELISA Kit*, R&D Systems, Catalog No. DY1679).

### Animal Experiments

C57BL/6 Mice were subcutaneously injected with 5^10^5^ MC38 cells suspended in 50 µL PBS. Mice were randomized into treatment groups when tumors reached ≈50 mm^3^ (n = 10 for each group). The length and width of tumors, weight of mice, were measured every 2 days. The volume of the tumor was calculated using the formula: 1/2 × D × d^2^ (D and d respectively, represent the major and minor axis). F_Apt_ (3 µm × 100 µL) or DFCs (DBC_150_‐A: 20 nm × 100 µL; DSC_90_‐A: 33.3 nm × 100 µL; DSOC_90_‐A: 33.3 nm × 100 µL) were intravenously injected into tumor‐bearing mice every other day. PD‐L1 antibody (1 mg kg^−1^, clone 10F.9G2, Bio‐XCell) was intraperitoneally injected on days 11, 15. ≈12 h after the last treatment on day 18, mice from each group were sacrificed, tumors and major organs (heart, liver, lung, kidney and spleen) were dissected, pictured, weighed and mechanically disaggregated, followed by digestion with collagenase type I for tumor and spleen ≈30 min at 37 °C. Then, mixtures were passed through 70 µm filters and washed using PBS to obtain the single‐cell suspension for flow cytometry analysis. Organs were fixed using 4% polyformaldehyde for immunohistochemical (IHC) analysis.

The in vivo investigation was performed three times. For the final experiment, each treatment group included 10 mice. Throughout the treatment period, all 10 mice per group were monitored for tumor volume and body weight. On Day 19, five mice per group were randomly selected for tissue harvesting and used for ex vivo tumor weight analysis, flow cytometry, and IHC analyses. The remaining five mice per group were used for survival analysis. Mice were sacrificed when the sizes of tumors reached 2 cm^3^, or when ulceration is found.

### Flow Cytometry Analysis of Immune Microenvironment of Mice

Tumor and spleen single‐cell suspensions of each mouse were counted and respectively stained with the three antibody panels shown in Table S7 (Supporting Information). Samples were analyzed using BD FACSVerse. Raw data were analyzed with Flowjo v10.10. The gating strategy was depicted in Figure S25 (Supporting Information).

### TGFβ1 Immunohistochemical (IHC) Analysis

Fixed organ sections were treated with 3% hydrogen peroxide for 25 min to block endogenous peroxidase activity. The sections were then blocked with 3% BSA at room temperature for 30 min and incubated with anti‐TGFβ1 (1:100; Abcam‐ ab215715) at 4 °C overnight. The sections were then incubated with an anti‐rabbit secondary antibody (1 ×; Tongling biology; DD23), followed by incubation with a freshly prepared 3,3′‐diaminobenzidine substrate solution to detect bound antibody. The sections were counterstained with hematoxylin and then with aqueous ammonia, dehydrated, and cover‐slipped.

The sections were scanned and then analyzed using ImageJ software, installed the plugin IHC Profiler. The specific operating procedure was: ImageJ‐plugins‐IHC Profiler‐Cytoplasmic stained image mode‐H DAB vectors. A demonstration of the operation process was shown in Figure S20 (Supporting Information).

### Statistical Analysis

Statistical analyses were performed by GraphPad Prism 8.2.1. Data are presented as the mean ± standard (s.d.) for all experiments. One‐way ANOVA with Tukey's multiple comparisons test, unpaired two‐tailed Student's t‐tests or log‐rank (Mantel–Cox) test were performed to determine the *p*‐values. To ensure figure clarity, the specific *p*‐values indicating the significance of group differences in Figure 3, 4, 5, Figures S16 and S19 (Supporting Information) were listed in Table S5 (Supporting Information).

### Animal Use and Care

C57BL/6J mice (male, 6–8 weeks old) were purchased from Jiesijie Laboratory Animal Center (Shanghai, China). All the animals were housed and fed in specific‐pathogen‐free environments with a temperature of 22 ± 1 °C, relative humidity of 50 ± 1%, and a light/dark cycle of 12/12 h. The animals were maintained in accordance with the guidelines of the Shanghai Medical Experimental Animal Care (AD20240221).

## Conflict of Interest

The authors declare no conflict of interest.

## Author Contributions

X.C. and D.L. contributed equally to this work. X.C. designed and performed most of the experiments, analyzed the data, arranged the figures, and prepared the manuscript. D.L. assisted in structure production, performed most of the animal experiments, and analyzed the data. J.J. assisted with the flow cytometry experiments. H.Y. assisted with the western blotting experiments. Y.S. assisted with the co‐culture assays. Y.L. provided valuable suggestions about the TCR‐T cell. J.S. and Y.Y. initiated the project, supervised the study, interpreted the data, and prepared the manuscript. All authors reviewed and approved the manuscript.

## Supporting information



Supporting Information

## Data Availability

The data that support the findings of this study are available in the supplementary material of this article.
