# Supplementary material for: Engineering DNA Origami Captors for TGFβ1 Sequestration to Enhance Tumor Immune Modulation and Therapy
Source: Adv Sci (Weinh). 2025 Jul 11;12(37):e06827. doi: 10.1002/advs.202506827 (PMC12499412; doi:10.1002/advs.202506827)
Supplement: Supplementary file 1 — Supporting Information [file ADVS-12-e06827-s001.docx]

**Supplementary Information**

**Engineering DNA Origami Captors for TGFβ1 Sequestration to Enhance Tumor Immune Modulation and Therapy**

Xiao Chen, Dunfang Liu, Jiahui Jin, Han Yao, Yao Sheng, Yarong Liu,
Jingwei Sun and Yang Yang

Contents

[Results 2](#_Toc91778415)

[Figure S1. 3](#_Toc91778416)

[Figure S2. 4](#_Toc91778416)

[Figure S3. 4](#_Toc91778416)

[Figure S4. 5](#_Toc91778417)

[Figure S5. 5](#_Toc91778418)

[Figure S6. 6](#_Toc91778419)

[Figure S7. 7](#_Toc91778420)

[Figure S8. 7](#_Toc91778421)

[Figure S9. 7](#_Toc91778422)

[Figure S10. 8](#_Toc91778423)

[Figure S11. 8](#_Toc91778424)

[Figure S12. 9](#_Toc91778425)

[Figure S13. 10](#_Toc91778425)

[Figure S14. 10](#_Toc91778425)

[Figure S15. 11](#_Toc91778425)

[Figure S16. 11](#_Toc91778425)

[Figure S17. 12](#_Toc91778425)

[Figure S18. 12](#_Toc91778425)

[Figure S19. 13](#_Toc91778425)

[Figure S20. 13](#_Toc91778425)

[Figure S21. 14](#_Toc91778425)

[Figure S22. 14](#_Toc91778425)

[Figure S23. 15](#_Toc91778425)

[Figure S24. 16](#_Toc91778425)

[Figure S25. 17](#_Toc91778425)

[Table S1. 18](#_Toc91778425)

[Table S2. 20](#_Toc91778425)

[Table S3. 42](#_Toc91778425)

[Table S4. 43](#_Toc91778425)

[Table S5. 44](#_Toc91778425)

[Table S6. 52](#_Toc91778425)

[Table S7. 52](#_Toc91778425)

[Table S8. 53](#_Toc91778425)

**Supplementary Figures**


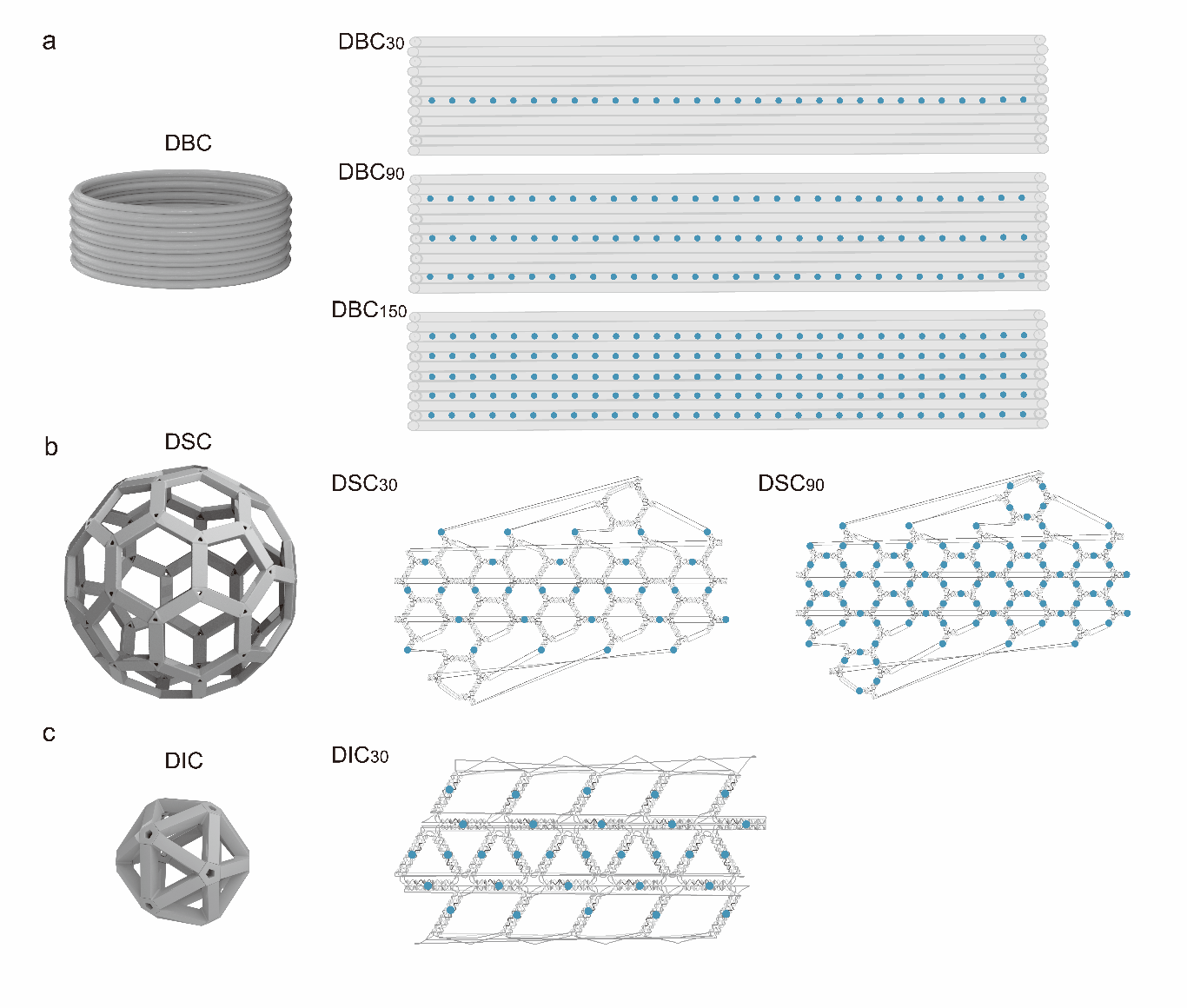


Figure S1. Scheme of the three DFCs in 3D models and 2D unfolded maps with aptamer modification sites indicated by blue dots.


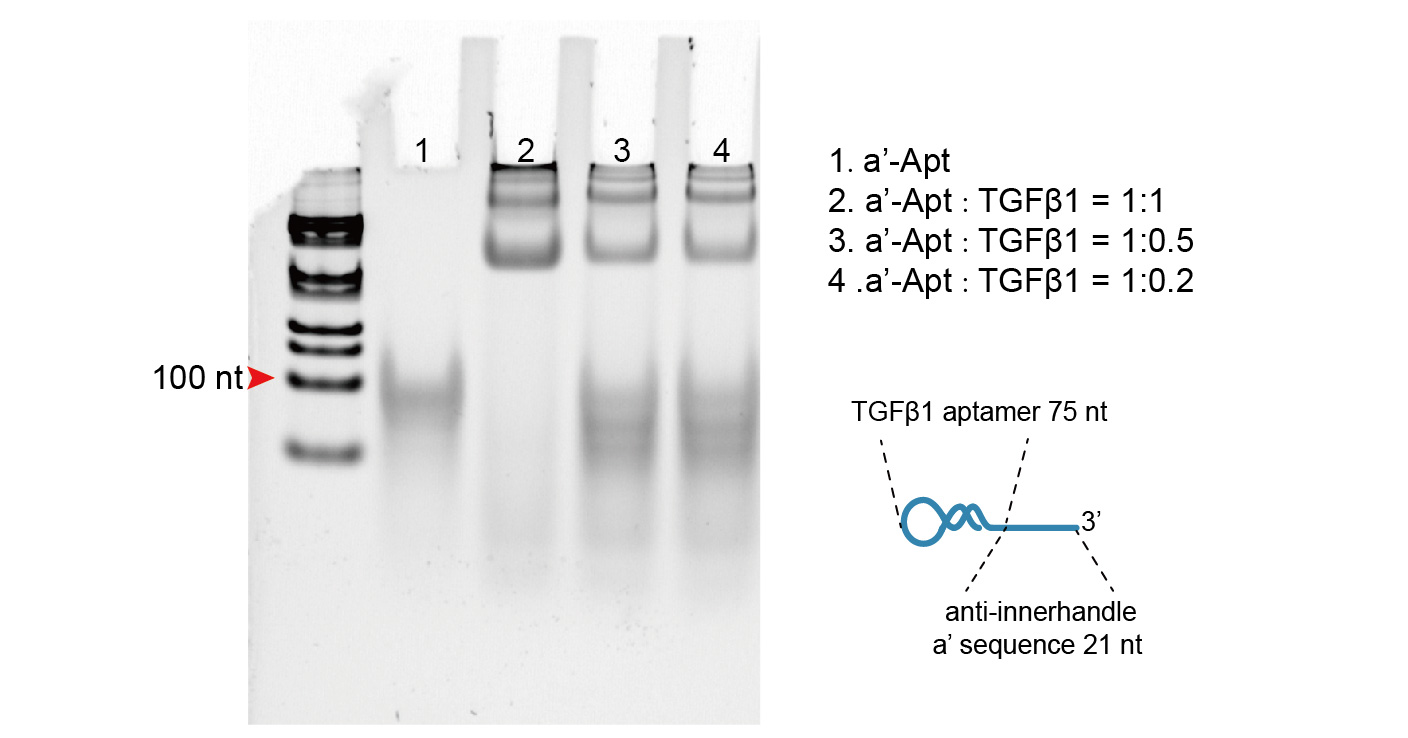


Figure S2. Native PAGE analysis of the a’-Apt (TGFβ1 aptamer) binds with TGFβ1 protein at titration ratios.

**
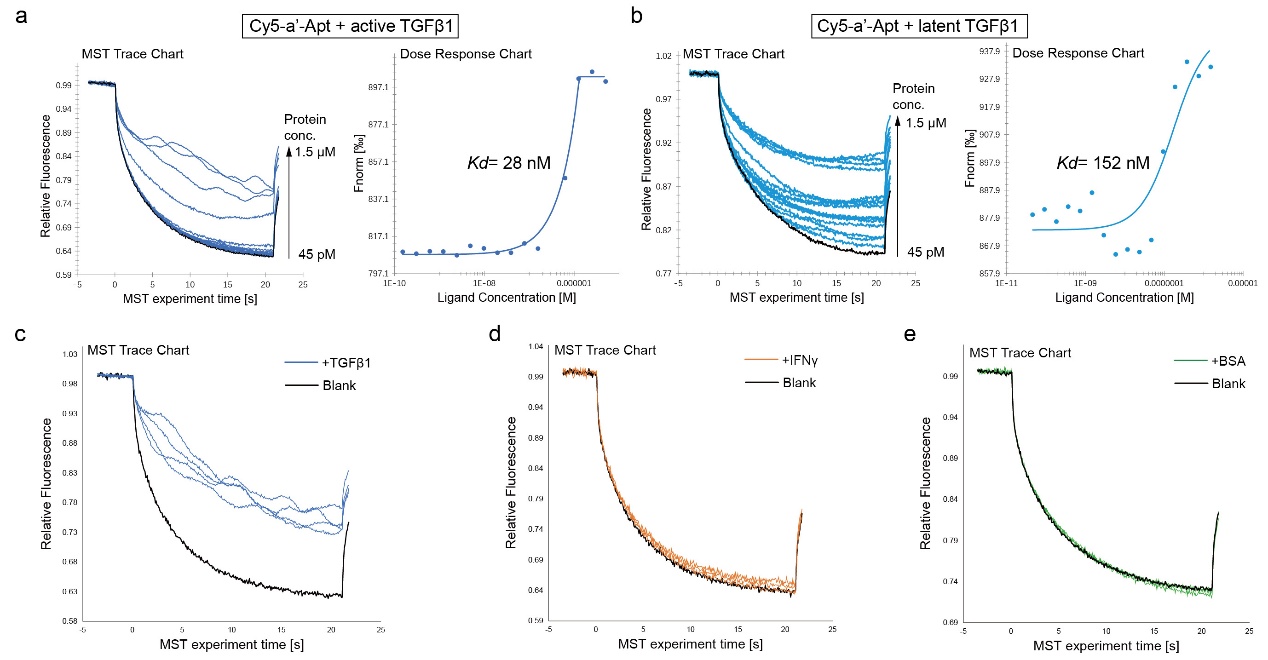
**

Figure S3. MST measurements verifying the protein binding affinity and specificity of the equipped aptamer (a’-Apt). For affinity assays, Cy5-labeled TGFβ1 aptamer was incubated with serially diluted TGFβ1 and latent TGFβ1, at concentrations ranging from 4.5 pM to 1.5 μM. For binding specificity detection, Cy5 labeled TGFβ1 aptamer respectively incubated with TGFβ1, IFNγ and BSA protein at normalized molar ratio (aptamer: protein= 1:50). A significant shift in the migration curve was observed only when incubated with TGFβ1 protein.


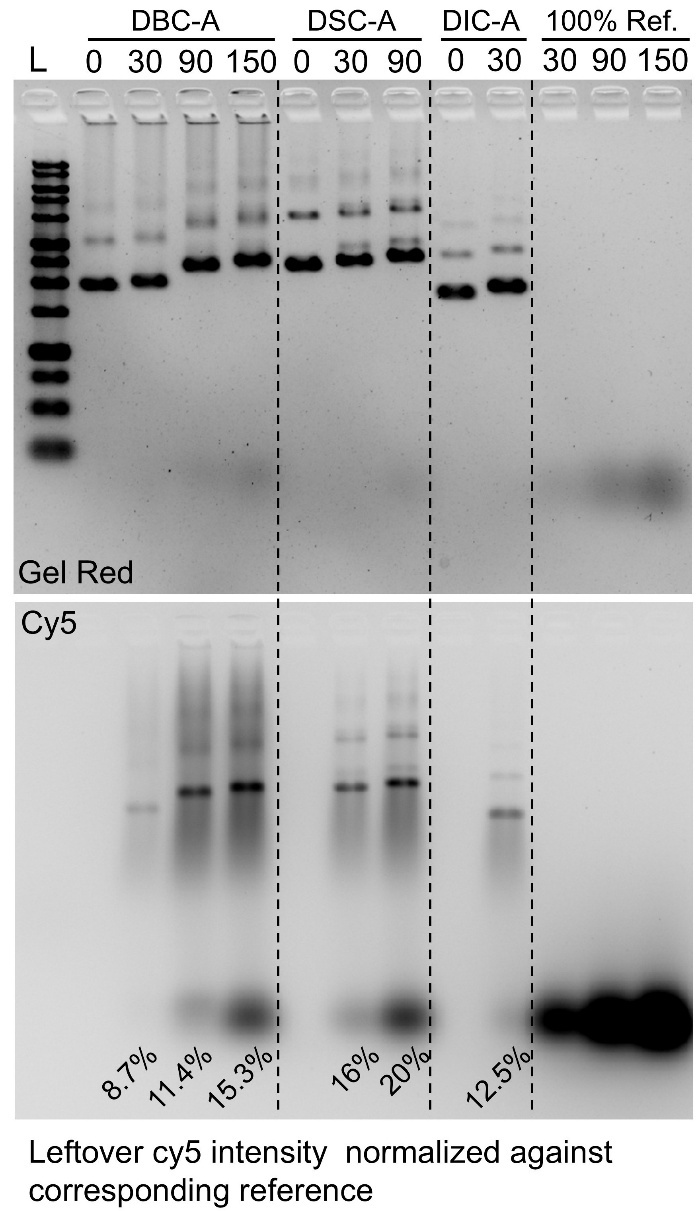


Figure S4. AGE analysis of the Cy5 labeled aptamer loading efficiency of DFCs under varying internal modification densities. Three DFCs (10 nM, 5 µL) were mixed with Cy5 labeled a’-Apt for 37°-2h. The unbound Cy5-a′-Apt was quantified and normalized against a 100% input control.


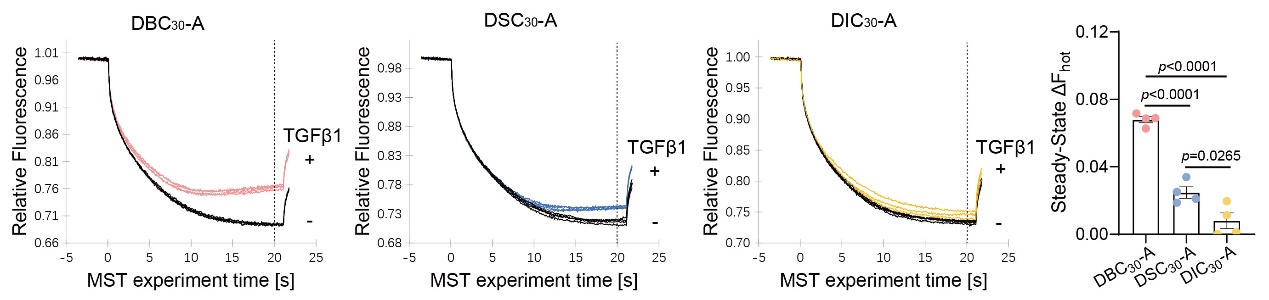


Figure S5. MST measurements verifying the TGFβ1-capturing function of aptamer-equipped DFCs and comparing the mobility shift.

**
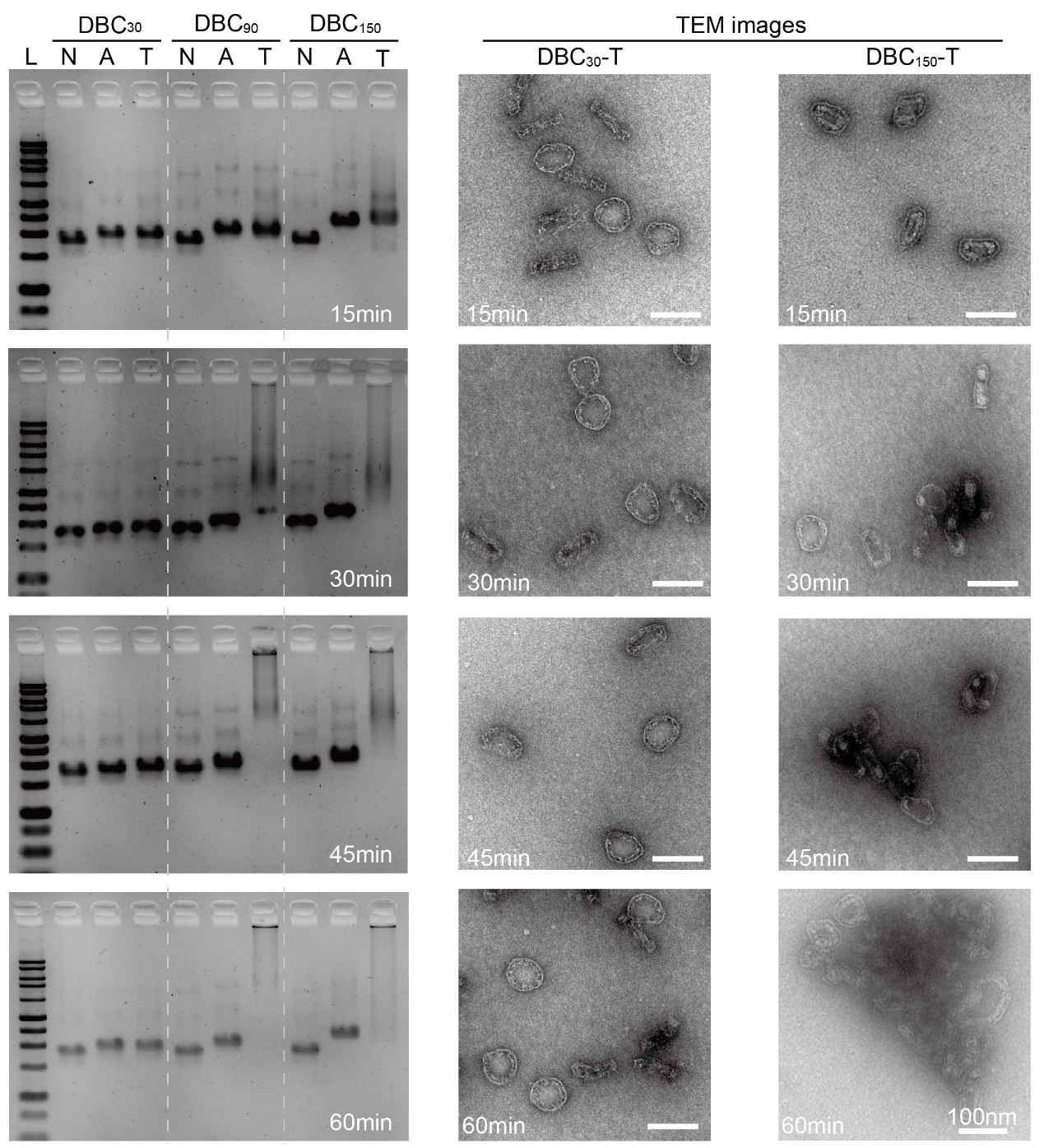
**

Figure S6. AGE images of DBC modified with 30, 90, and 150 internal aptamers incubated with TGFβ1 at different time point. (N: non-modified, A: aptamer-modified, and T: TGFβ1-incubated DFCs). TEM images comparing the morphology changes of DBC_30_-T and DBC_150_-T at different time points.


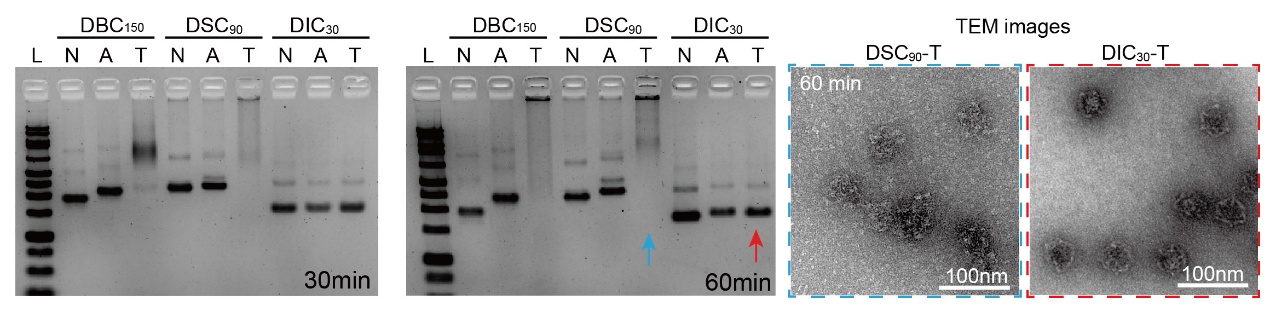
Figure S7**.** Agarose gel electrophoresis results of three types of DFCs with saturated internal aptamer modification incubated with TGFβ1 for 30 and 60 min, and TEM images of DSC_90_-T and DIC_30_-T products after the capturing process (N: non-modified, A: aptamer-modified, and T: TGFβ1-incubated DFCs).


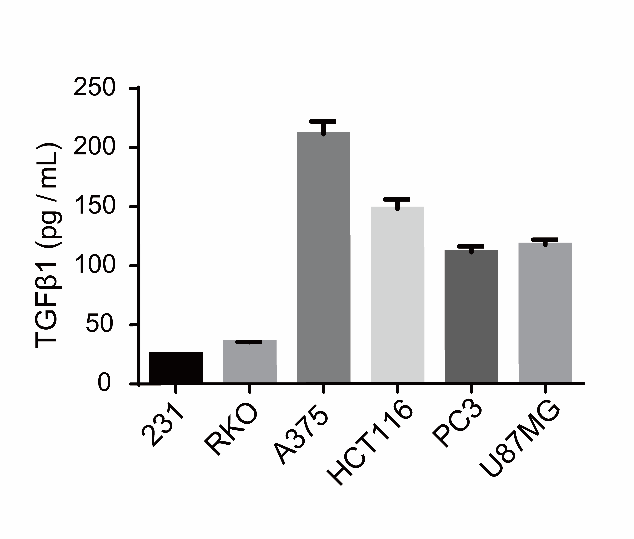


Figure S8. ELISA quantification results of TGFβ1 secretion in the supernatant of six cell lines, expressed in pg/mL/1×10^6^ cells/24h.


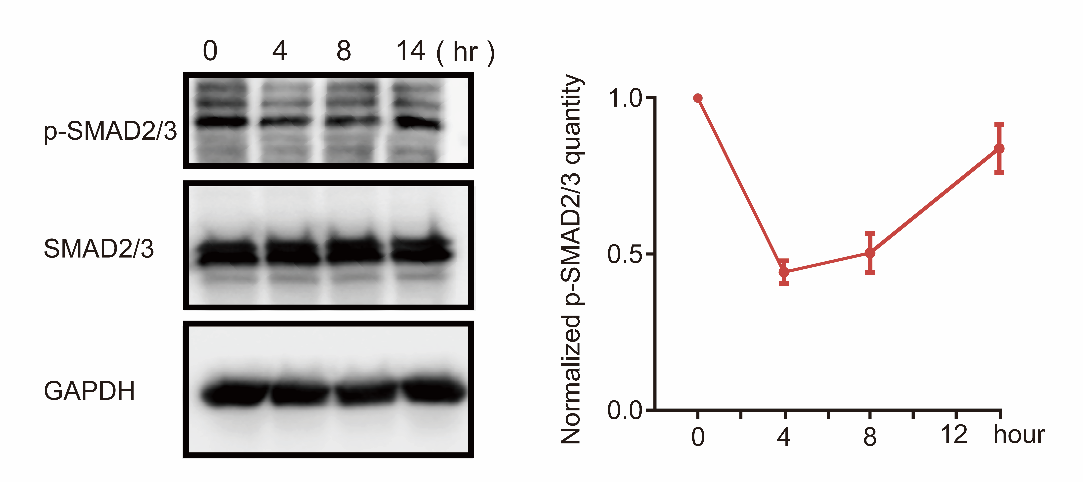


Figure S9. Western blotting images and quantified band intensity of p-SMAD2/3 levels at different time point through a 14h treatment of tumor cells with DBC_150_-A.


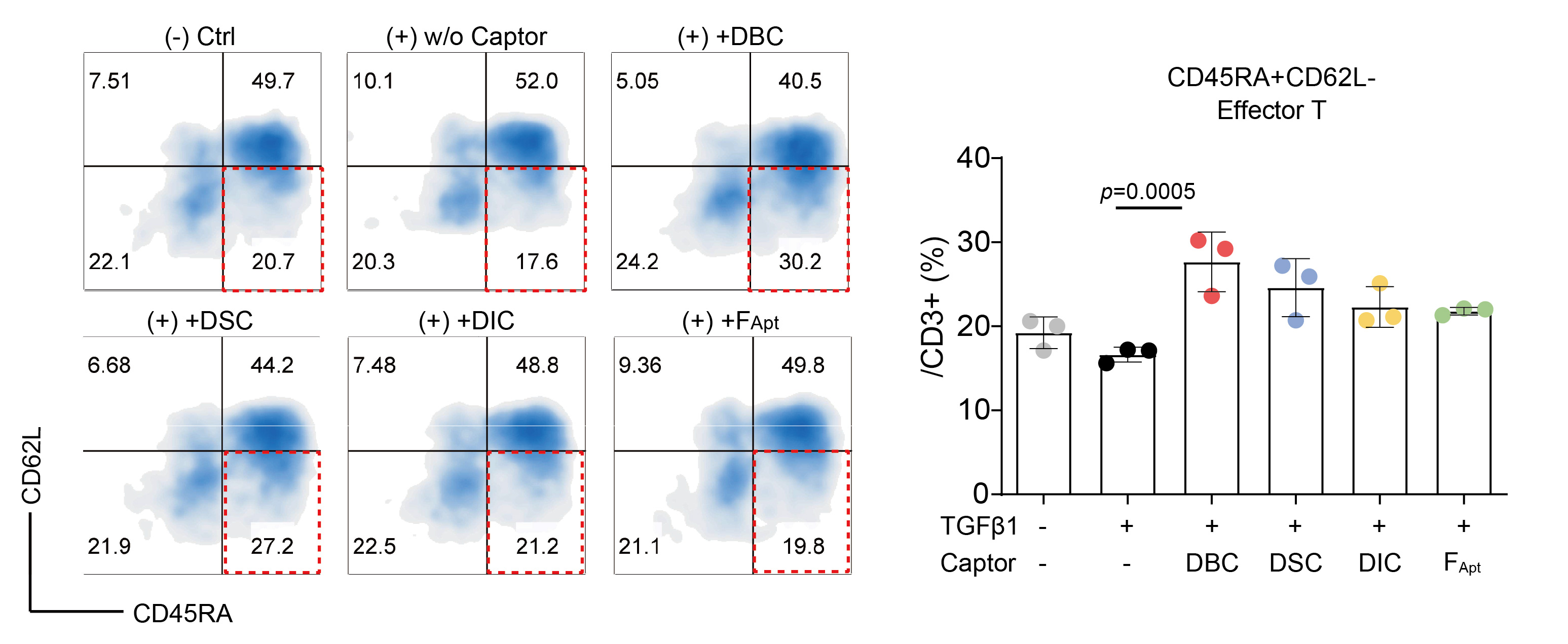


Figure S10. Flow cytometry results of CD3+CD45RA+CD62L- effector T cell population after incubating PBMC with DFCs, followed by statistical analysis.


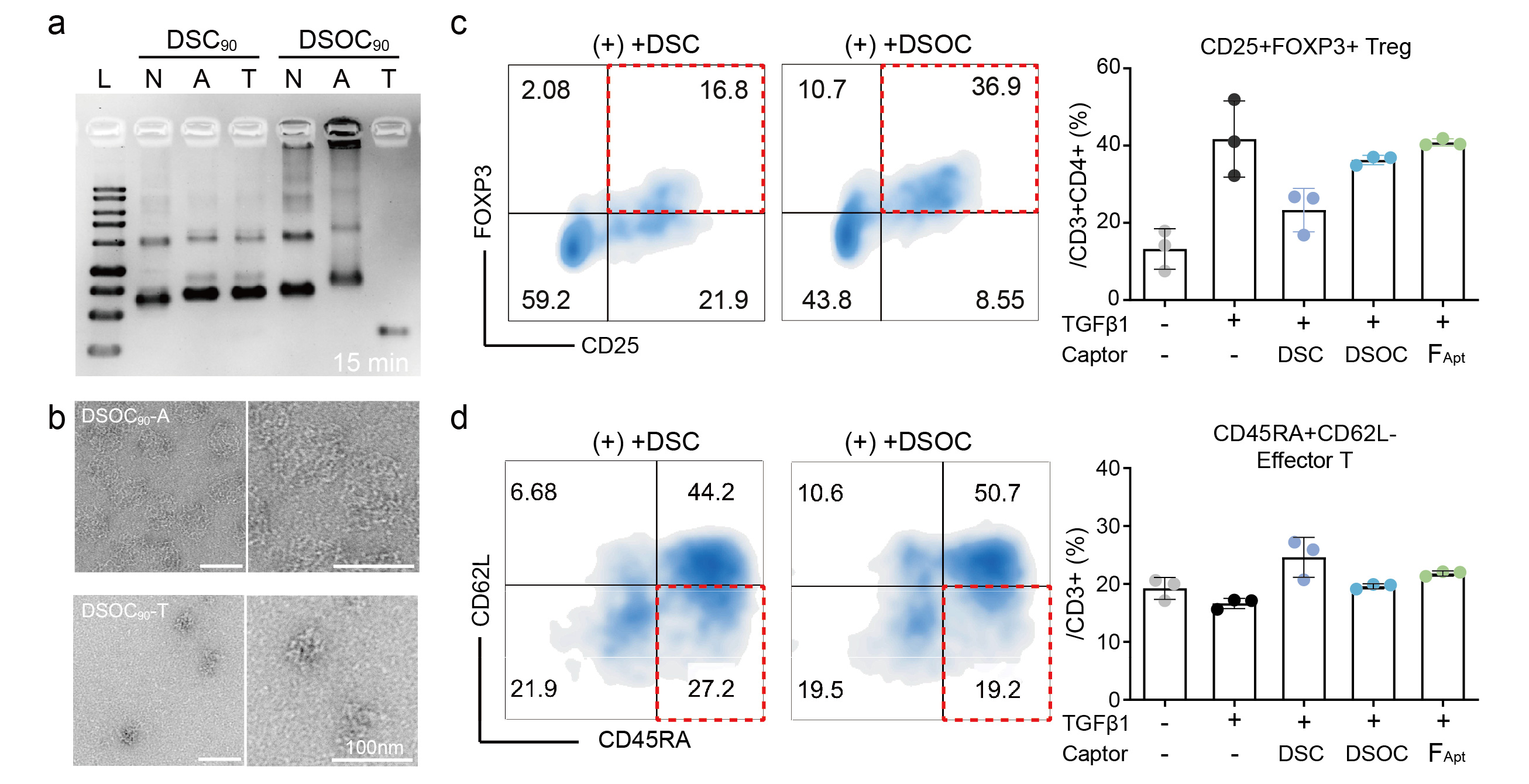


Figure S11. Comparison TGFβ1 capture capability with DSC and DSOC: (a) Agarose gel electrophoresis and (b) TEM images of DSC_90_-A90 with 90 internally modified aptamers and DSOC_90_-A with 90 externally modified aptamers. (c) Proportion of Treg cells in PBMCs via DSC or DSOC treatments. (d) Proportion of effector T cells in PBMCs via DSC or DSOC treatments.


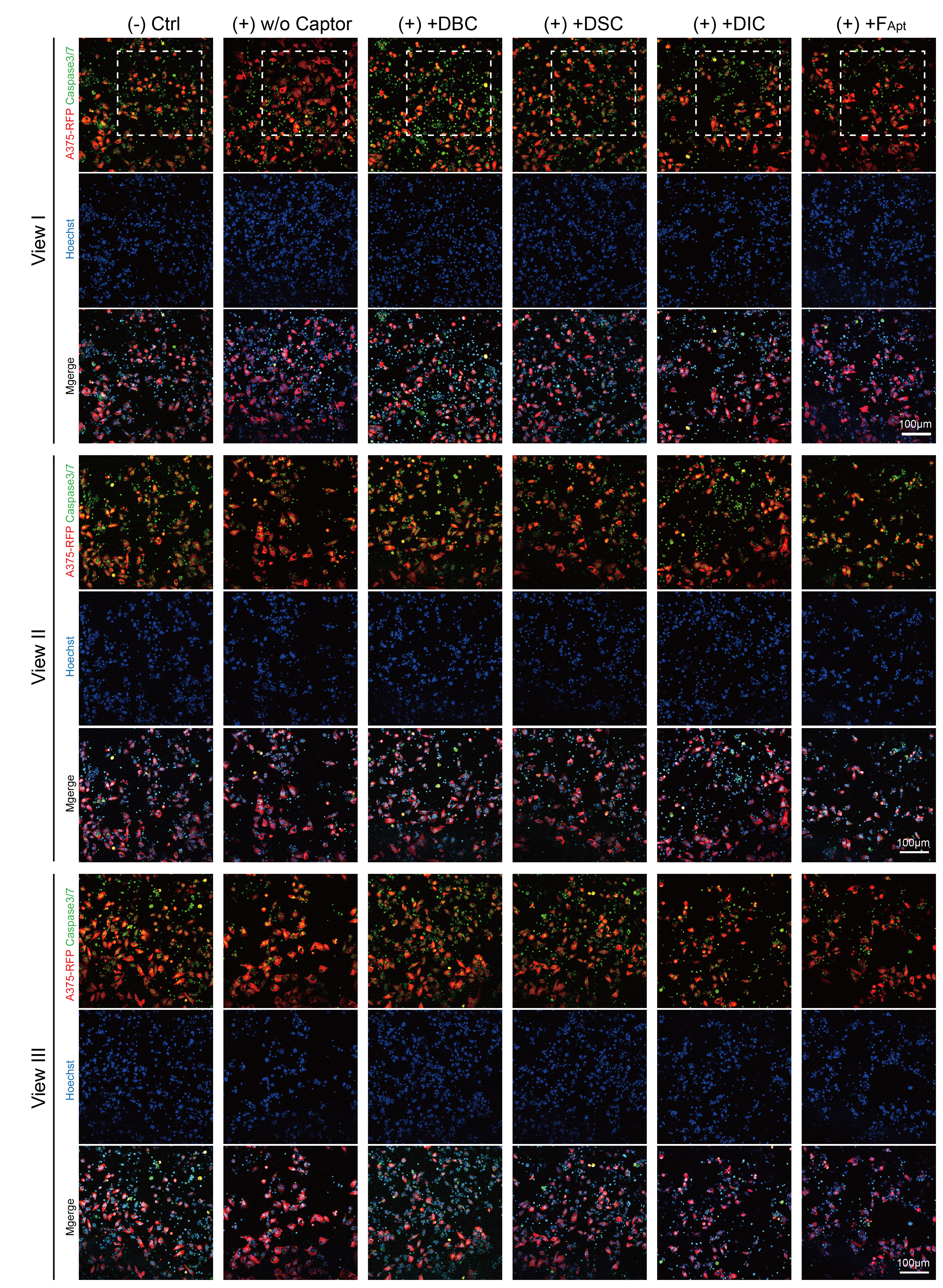


Figure S12. Three group of representative confocal images of PBMC killing assay against A375-RFP tumor cells. (+) or (-) reflects if additional TGFβ1 was supplemented. The squared areas from images in the first row were displayed in Figure 3.


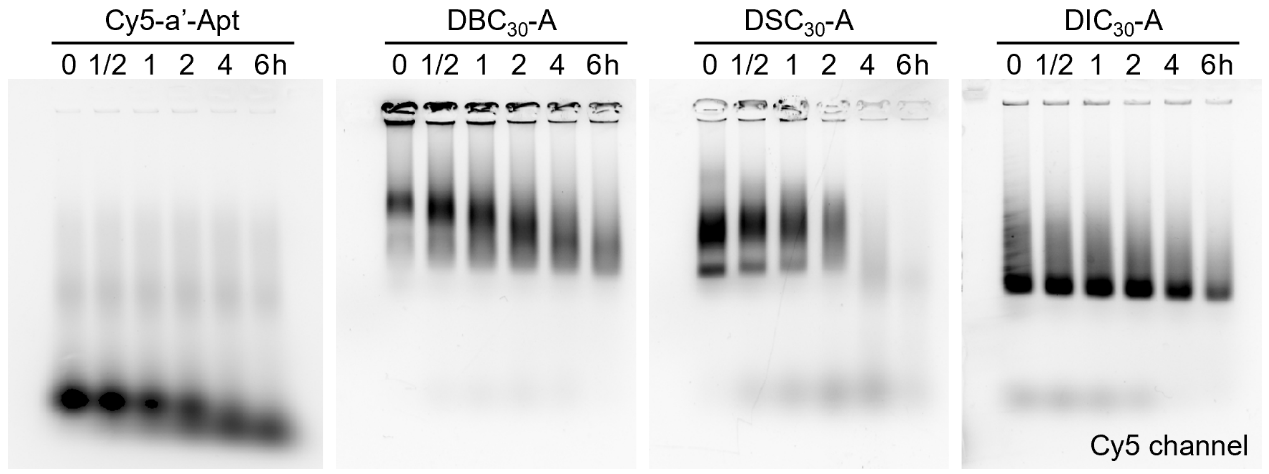


Figure S13. Serum stability assessment of free Cy5-a′-Apt and 30-aptamer-conjugated DFCs. Free aptamer and aptamer-conjugated DFCs were incubated at the same molar concentration (0.05pmole) in 80% FBS for various durations, followed by analysis using agarose gel electrophoresis.

**
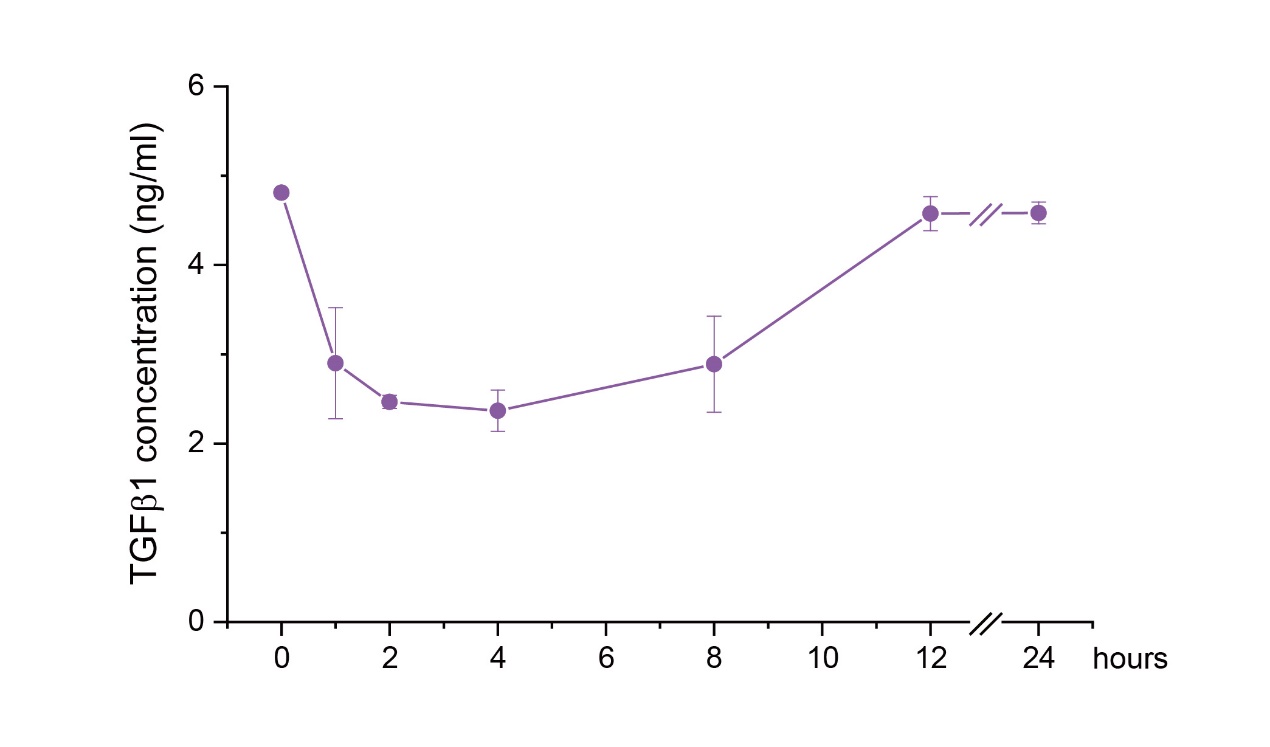
**

Figure S14. Circulating TGFβ1 concentration at different time points after tail vein injection of DBC for TGFβ1 clearance.


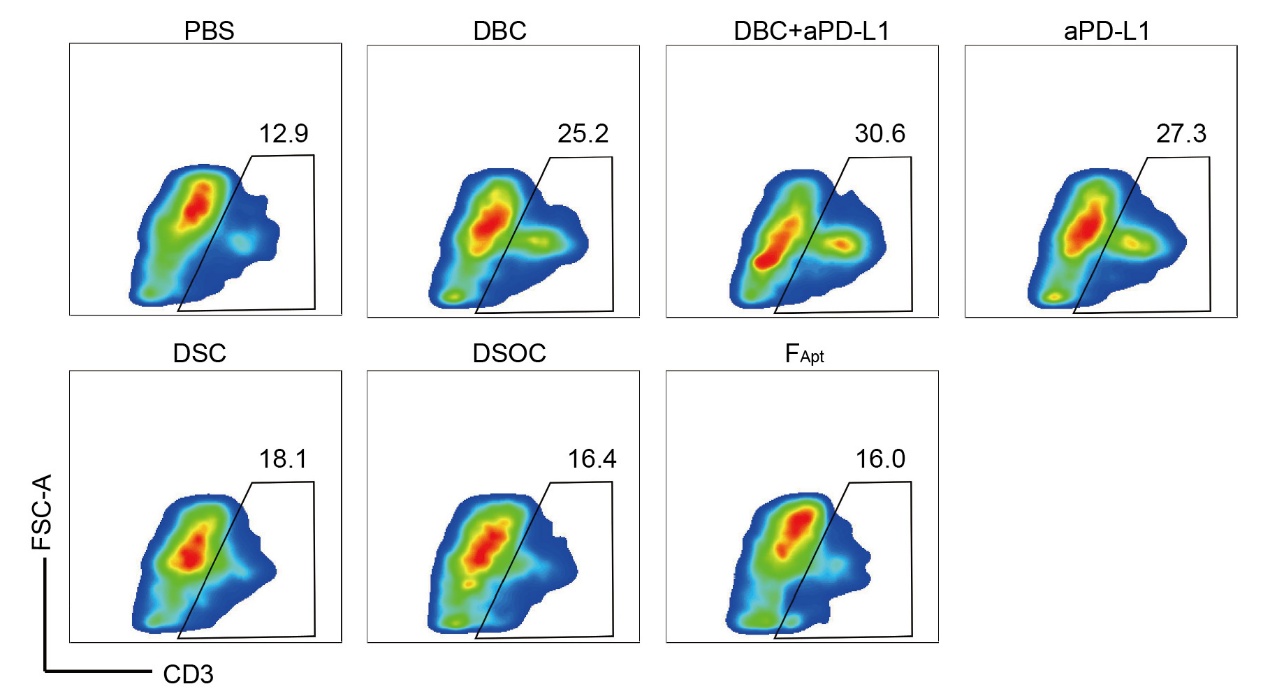


Figure S15. Flow cytometry gating plots of tumor-infiltrating CD45+CD3+ T cells in each group.


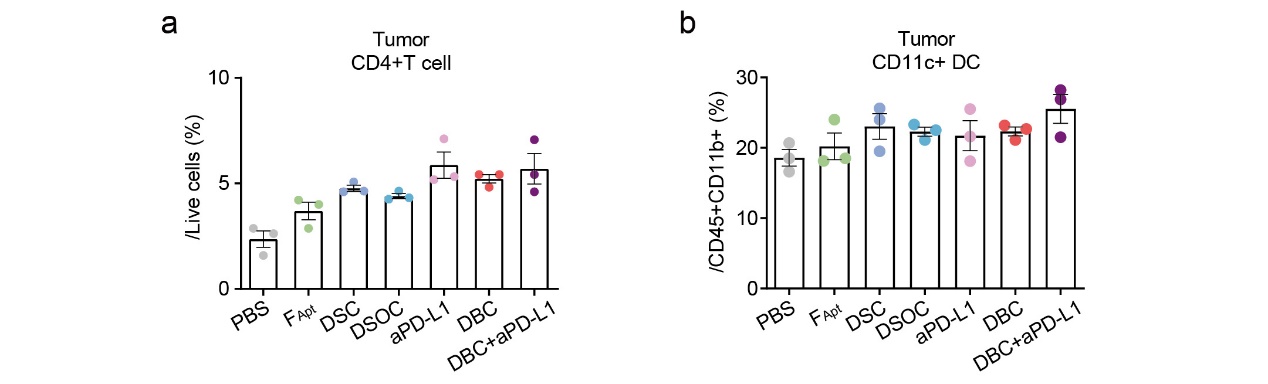


Figure S16. Flow cytometry analysis of tumor-infiltrating CD4+ T cells (a) and DCs (b) across different experimental groups *in vivo*.

**
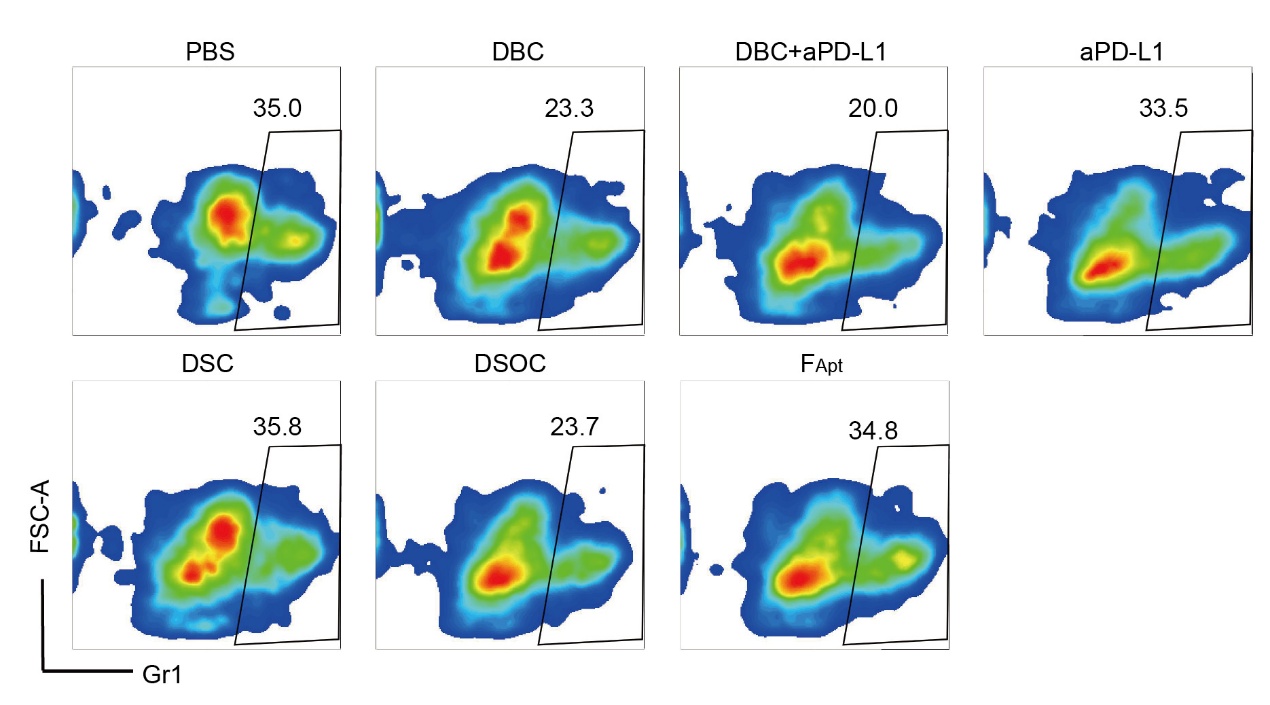
**

Figure S17. Flow cytometry gating plots of tumor-infiltrating CD45+CD11b+Gr1+ MDSC cells in each group.

**
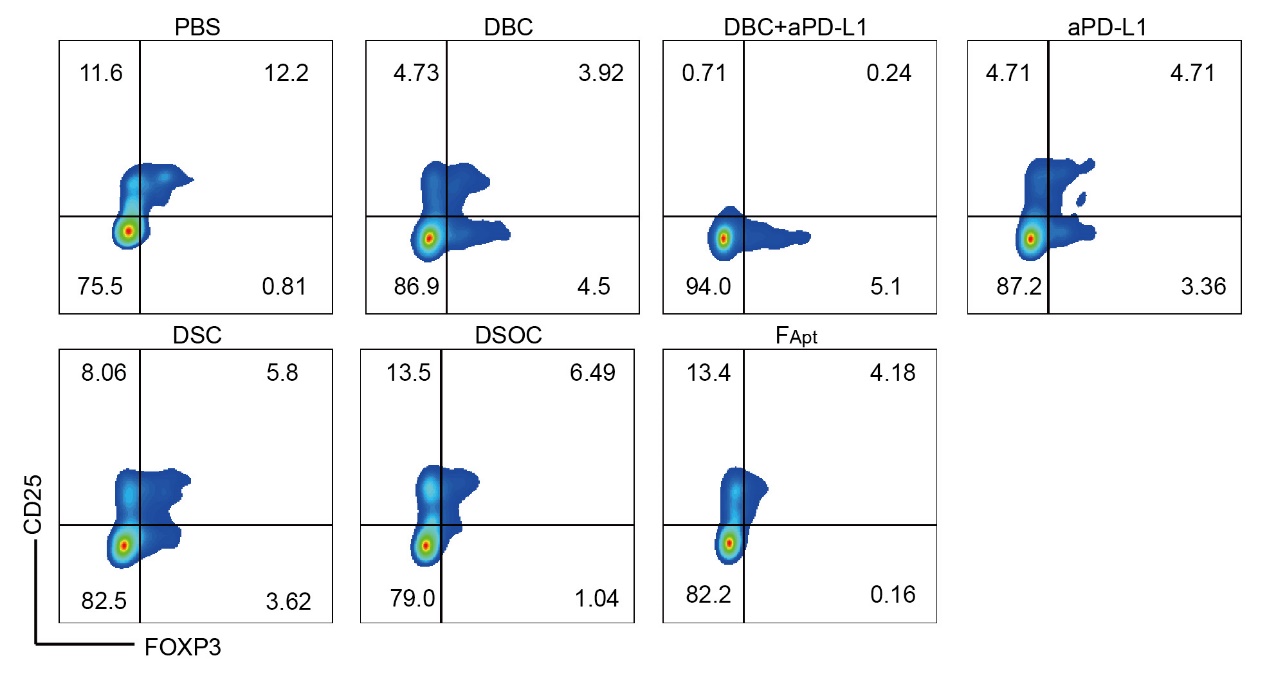
**

Figure S18. Flow cytometry gating plots of spleen CD3+CD4+CD25+FOXP3+ Treg cells in each group.

**
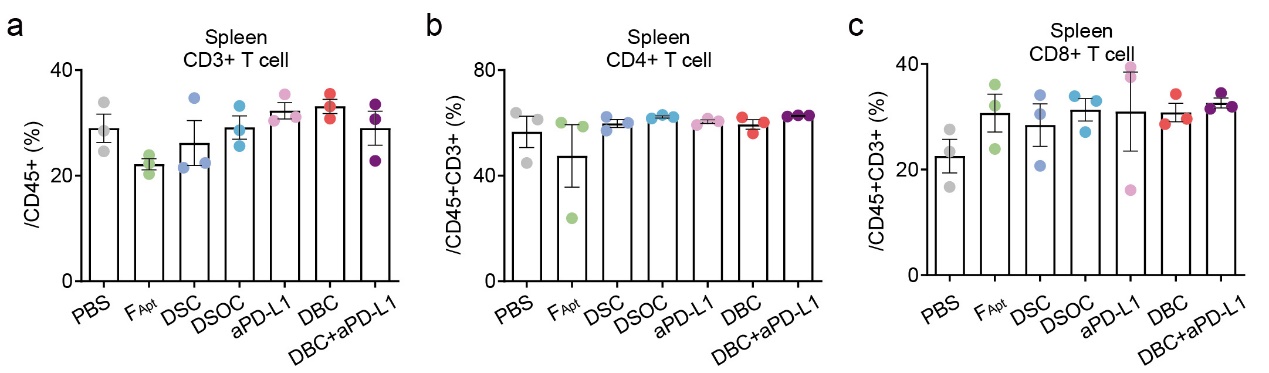
**

Figure S19. Flow cytometry data for CD3+ T cells (a), CD4+ T cells (b), and CD8+ T cells (c) in the spleens of mice from each group.

**
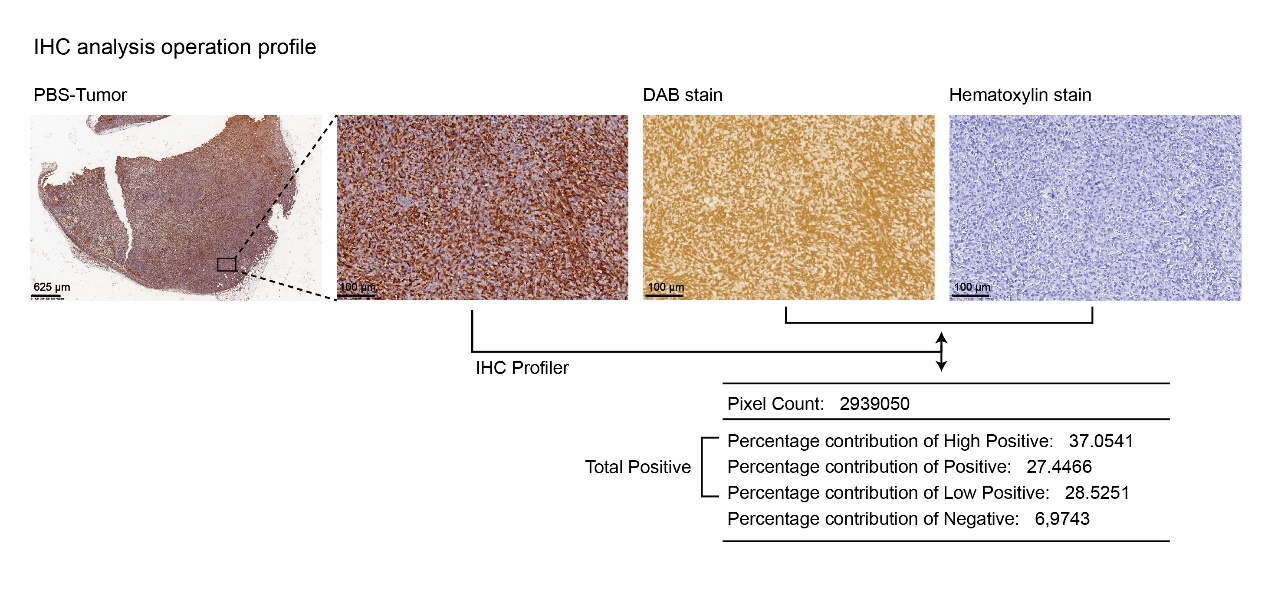
**

Figure S20. An example demonstrating the IHC processing and quantification method.


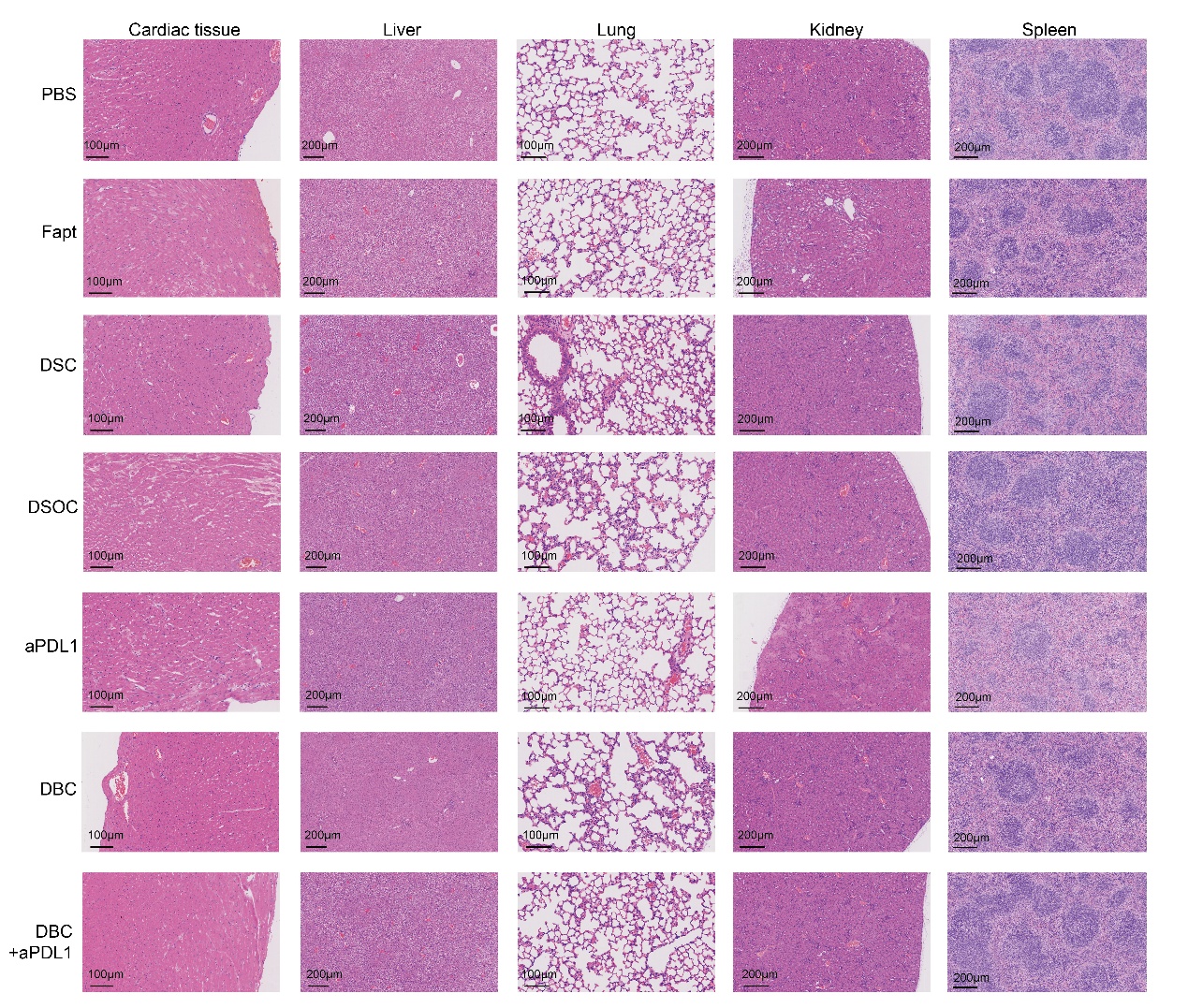


Figure S21. Representative H&E staining images of cardiac tissue, liver, lung, kidney and spleen for each group.

**
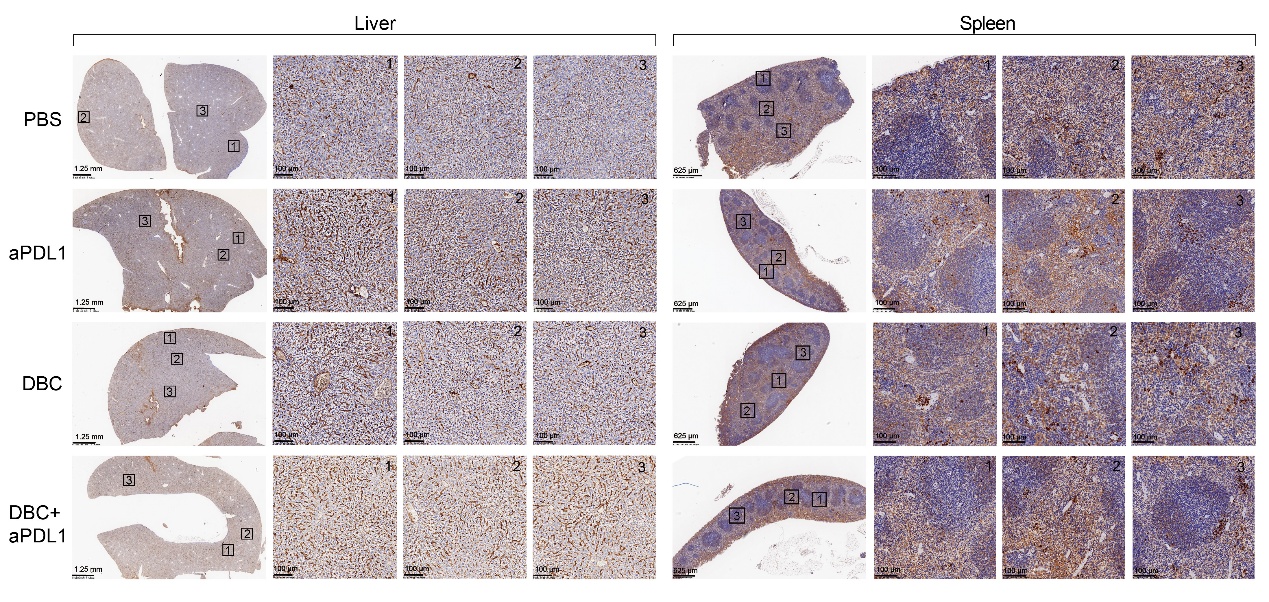
**

Figure S22. Representative liver and spleen TGFβ1 IHC images from mice treated by PBS, aPD-L1, DBC and the combined therapy.

**
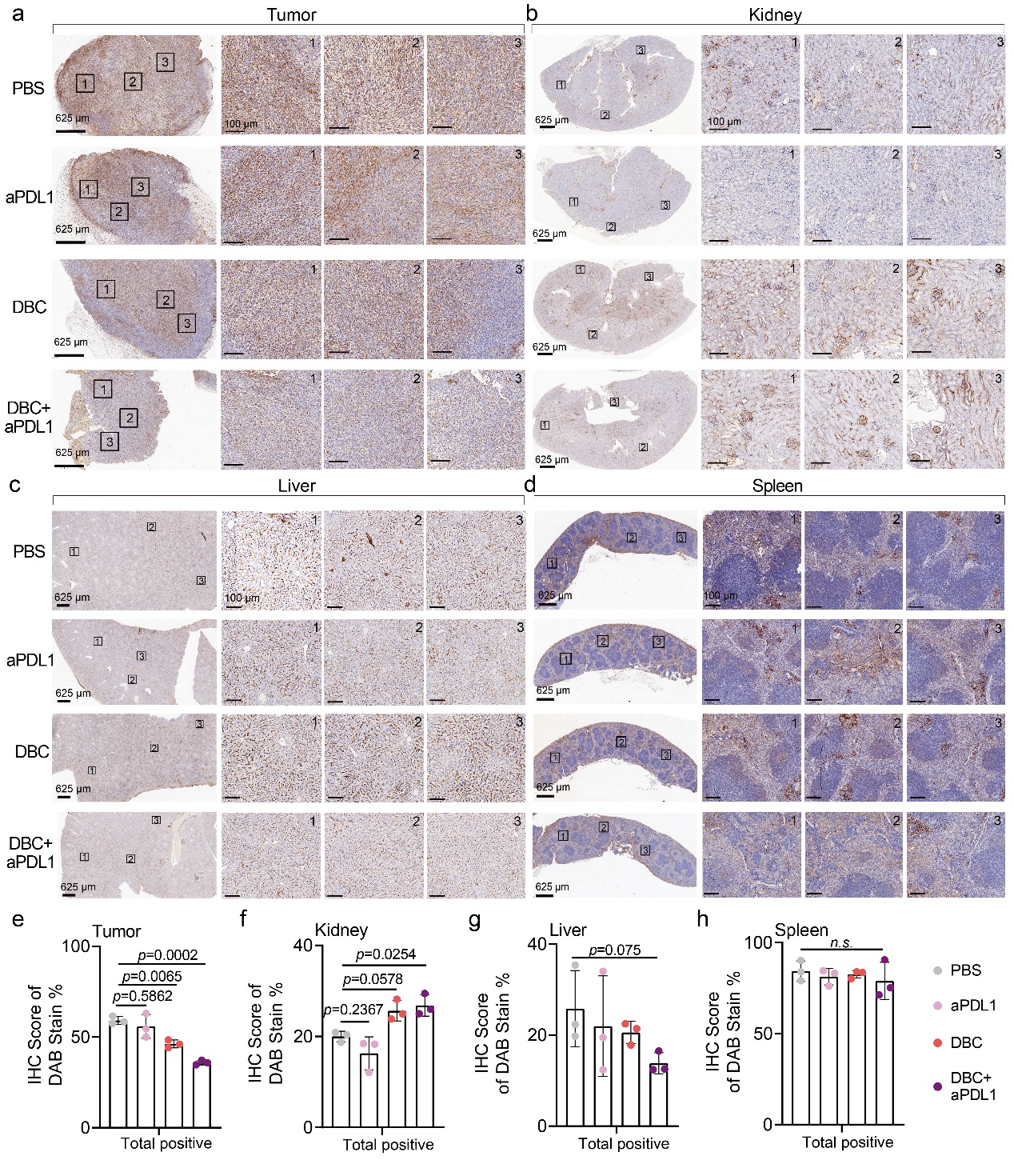
**

**Figure S23.** Representative TGFβ1 IHC images of tumor, kidney, liver, and spleen from additional individuals in each group (distinct from those in **Figures 5** and **S22**).


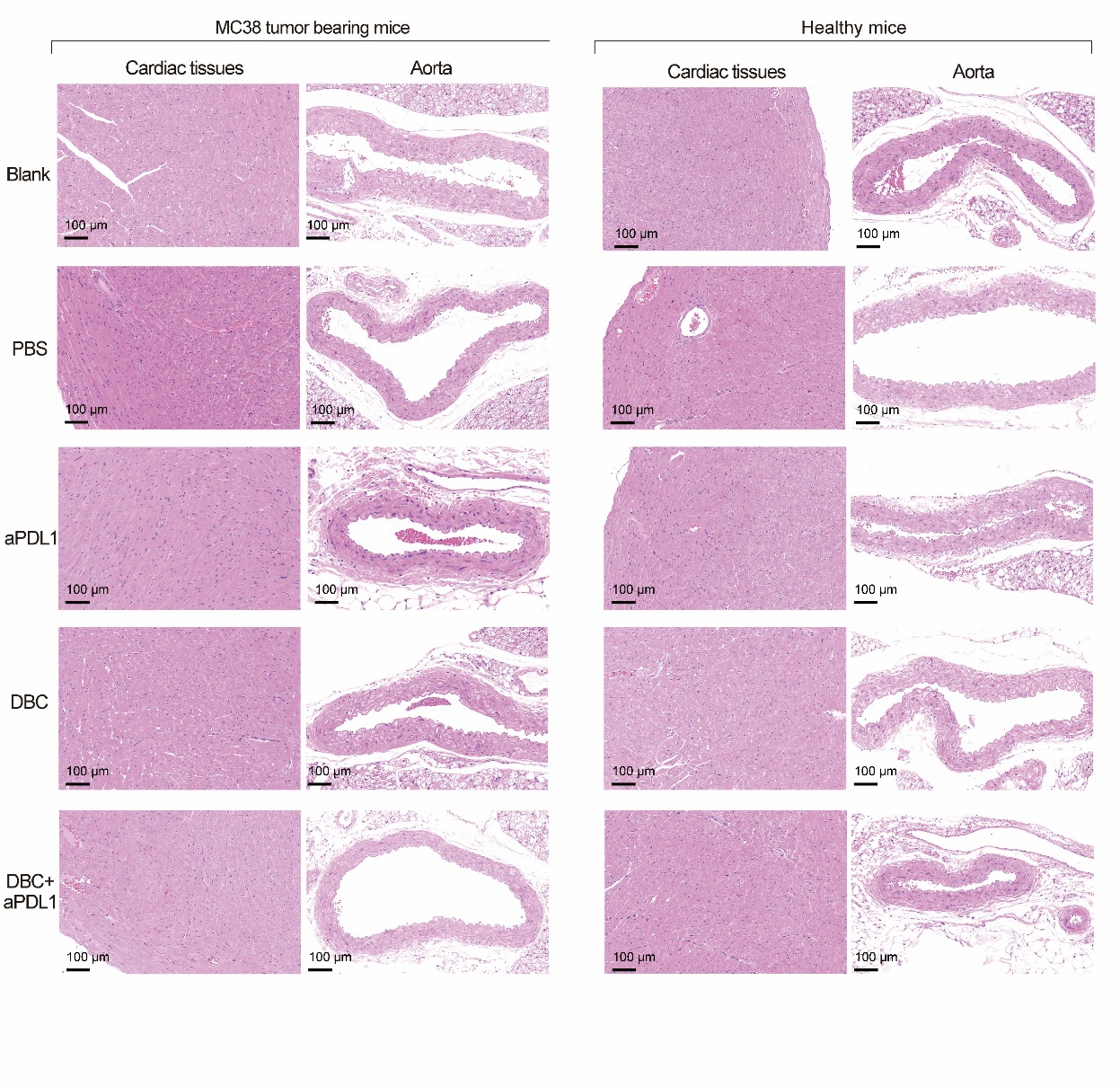


Figure S24. Representative cardiac tissues and aorta H&E staining images of MC38 tumor-bearing mice and healthy mice treated with PBS, aPD-L1, DBC, or the combination injection.


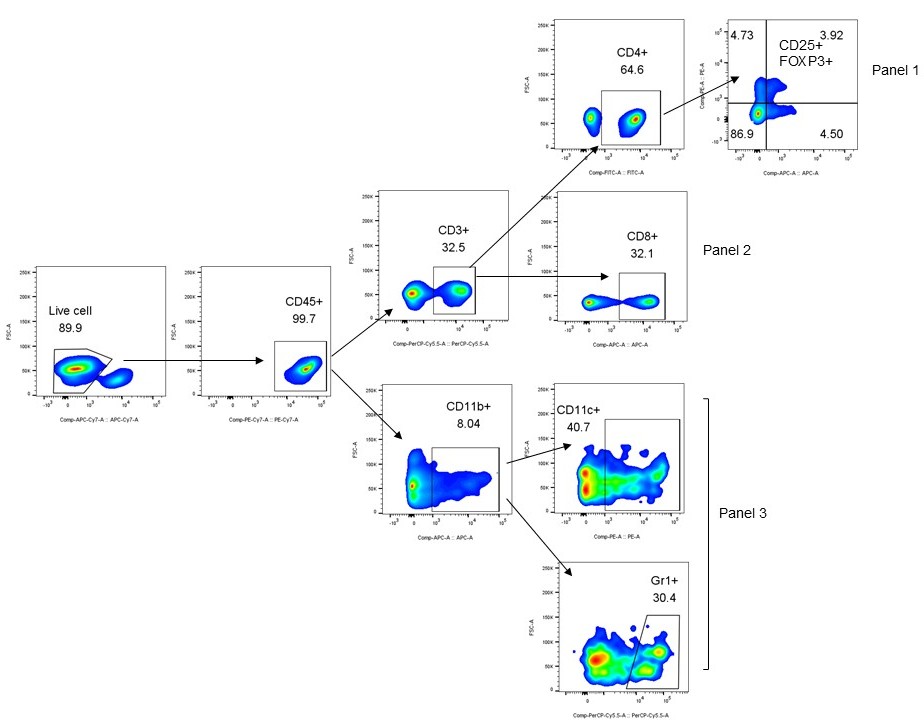


Figure S25. A presentative example showing flow cytometry gating strategy in animal experiments.

**Supplementary Tables**

**Table S1. Dosage and administration of reported antagonistic TGFβ antibodies, fusion proteins and small molecule inhibitors**

|  | **Dosage** | **Method of administration** |
| --- | --- | --- |
| ^[1]^anti-CTLA4-TGFβRII | 5mg/kg  125μg/mouse  13.9μM×100μL/mouse | Intraperitoneal injection  Once a week |
| ^[1]^Anti-PDL1- TGFβRII | 5mg/kg  125μg/mouse  12.5μM×100μL/mouse | Intraperitoneal injection  Once a week |
| ^[2]^4T-Trap (fused antibodies for CD4+TGFβRII) | 5mg/kg  125μg/mouse  6.6μM×100~200μL/mouse | Intravenous injection  Twice a week |
| ^[3]^YM101 (fused antibodies targeting PDL1 and TGFβ) | 9mg/kg  225μg/mouse | Intraperitoneal injection  Every other day |
| ^[4-6]^M7824 (anti-PDL1 and TGFβ fusion protein, TGF-β trap) | 20mg/kg  500μg/mouse  27.8μM×100μL/mouse | Intraperitoneal injection  Every other day |
| ^[7-9]^LY2157299 (Galunisertib) : | 45-75 mg/kg  1.125~1.875 mg/mouse  3~5.1 mM×200μL/mouse | Oral infusion  Once a day |
| ^[10]^Tranilast: TGFβ inhibitor | 200~300mg/kg  5~7.5 mg/ mouse  15.3~23 mM×200μL/mouse | Oral infusion  Twice a day |

* Mouse body weight is estimated to be 25g in all the above studies

1. Ravi, R. et al. Bifunctional immune checkpoint-targeted antibody-ligand traps that simultaneously disable TGFβ enhance the efficacy of cancer immunotherapy. Nat Commun 9, 741, doi:10.1038/s41467-017-02696-6 (2018).
2. Li, S. et al. Cancer immunotherapy via targeted TGF-β signalling blockade in TH cells. Nature 587, 121-125, doi:10.1038/s41586-020-2850-3 (2020).
3. Yi, M. et al. The construction, expression, and enhanced anti-tumor activity of YM101: a bispecific antibody simultaneously targeting TGF-β and PD-L1. J Hematol Oncol 14, 27, doi:10.1186/s13045-021-01045-x (2021).
4. Lan, Y. et al. Enhanced preclinical antitumor activity of M7824, a bifunctional fusion protein simultaneously targeting PD-L1 and TGF-β. Sci Transl Med 10, doi:10.1126/scitranslmed.aan5488 (2018).
5. Horn, L. A. et al. Remodeling the tumor microenvironment via blockade of LAIR-1 and TGF-β signaling enables PD-L1-mediated tumor eradication. J Clin Invest 132, doi:10.1172/JCI155148 (2022).
6. Lan, Y. et al. Simultaneous targeting of TGF-β/PD-L1 synergizes with radiotherapy by reprogramming the tumor microenvironment to overcome immune evasion. Cancer Cell 39, doi:10.1016/j.ccell.2021.08.008 (2021).
7. Brandes, A. A. et al. A Phase II randomized study of galunisertib monotherapy or galunisertib plus lomustine compared with lomustine monotherapy in patients with recurrent glioblastoma. Neuro Oncol 18, 1146-1156, doi:10.1093/neuonc/now009 (2016).
8. Wang, Y. et al. Co-inhibition of the TGF-β pathway and the PD-L1 checkpoint by pH-responsive clustered nanoparticles for pancreatic cancer microenvironment regulation and anti-tumor immunotherapy. Biomater Sci 8, 5121-5132, doi:10.1039/d0bm00916d (2020).
9. Yingling, J. M. et al. Preclinical assessment of galunisertib (LY2157299 monohydrate), a first-in-class transforming growth factor-β receptor type I inhibitor. Oncotarget 9, 6659-6677, doi:10.18632/oncotarget.23795 (2018).
10. Panagi, M. et al. TGF-β inhibition combined with cytotoxic nanomedicine normalizes triple negative breast cancer microenvironment towards anti-tumor immunity. Theranostics 10, 1910-1922, doi:10.7150/thno.36936 (2020).

**Table S2. Staple strands sequence Information of all the three DFCs.**

| DNA Barrel Captor | | |
| --- | --- | --- |
| Name | **Sequence(5’-3’)** | **Length (nt)** |
| DBC-Main-01 | GTTTTAGAAAATAATGTAAATCCTGATT | 28 |
| DBC-Main-02 | GTATTCTTACGAGCTGAGTGATTACAAA | 28 |
| DBC-Main-03 | CAGATATCAATAATCAGTACAAATTATT | 28 |
| DBC-Main-04 | CGCGCCCTATCATTAATTACCGAGCAAA | 28 |
| DBC-Main-05 | TTCATCGAAACCAAACATTTAAAACATC | 28 |
| DBC-Main-06 | GCGTCTGATGTGAGAGTCACGATGTTCT | 28 |
| DBC-Main-07 | AACGCCACGGATTCGGTAACGATTCATG | 28 |
| DBC-Main-08 | GCTCATTCGGCGGATGCTGCAGTCCTTA | 28 |
| DBC-Main-09 | ATTAAATTAGGTCACAGCTGGCCTTATG | 28 |
| DBC-Main-10 | TAATATTGGCGCATCGGGCCTAAATAAC | 28 |
| DBC-Main-11 | AAATATTTGCCAGTTGTTGGGATTTACG | 28 |
| DBC-Main-12 | AACAGGACAGTATCCGCCATTCTCTATG | 28 |
| DBC-Main-13 | GATAATCCGCACTCGAAACCAGCCCTGC | 28 |
| DBC-Main-14 | TTAACTGATCATAACCGACCGAAAAGTT | 28 |
| DBC-Main-15 | GAAGCGCCTGTTTATAAATTTATTTTGC | 28 |
| DBC-Main-16 | AGAGAATATACAAAAGTTAATACCAGAA | 28 |
| DBC-Main-17 | GAAAATAAGCCAACAACTTTTTCATATT | 28 |
| DBC-Main-18 | TTTTTGTCTTAATTGCAAGACGATGGCA | 28 |
| DBC-Main-19 | CCAATCCTTAACAAAATGCTGTCCTGAT | 28 |
| DBC-Main-20 | AAACAGCTAGGCAGGGGTTATTCTGAAT | 28 |
| DBC-Main-21 | CTAATTTCAGTAATTTTTAACAACCTAC | 28 |
| DBC-Main-22 | TAACGAGTACCGACCATAGGTTTGCACG | 28 |
| DBC-Main-23 | ATCCTGATCTGTCCAATAGTGGAAATTG | 28 |
| DBC-Main-24 | TTTGCACAACAACAATTAAGATTTAACG | 28 |
| DBC-Main-25 | AAATCAAGAACGCGAAAACATGTAACAG | 28 |
| DBC-Main-26 | TGCGGGAATAGATAAATTTTCGGAGAAA | 28 |
| DBC-Main-27 | AATAGAAAGGAAACAGTTTTACATCTTTAGAAGGA | 35 |
| DBC-Main-28 | ATGACAACAACGCCCAGTACCCATTAGCAAGTTTA | 35 |
| DBC-Main-29 | CATAACCTTCGTCAGTCGAGAATCACCATAGAAAA | 35 |
| DBC-Main-30 | CTGAGGCCCATGTAATAGCCCTTGGGAACAGCGCC | 35 |
| DBC-Main-31 | AGGCCGCGATAGCAACCGTACACCGTCAGACATTC | 35 |
| DBC-Main-32 | ACCCTCACCACCACCCGCCACTTCATTAGGAAGGT | 35 |
| DBC-Main-33 | GGCTACAGCTCAACTGGAAGTATATTTTCCGGAGA | 35 |
| DBC-Main-34 | AAAGACTTTGCTGAAGTTGATAAAATTTCAATATG | 35 |
| DBC-Main-35 | TTTCCATTGCGGATACGAGTACTTTATTTAGCTGA | 35 |
| DBC-Main-36 | CGTAATGTTTTGATTTAGATAAATACTTGAGGGTA | 35 |
| DBC-Main-37 | CCAACCTGAGAGTATCAATAACCAAAAAATCTACA | 35 |
| DBC-Main-38 | AAAAGAAAACTCCATTTTCATCATAAAGATTGCCT | 35 |
| DBC-Main-39 | ATCTTTGAGCGAACAAAAGGTTAAGCAAACAAGAG | 35 |
| DBC-Main-40 | TACCAAGGCGTTTTTAATAGTAAGGCAAAATCGTA | 35 |
| DBC-Main-41 | AGCCGGATTTAATCGATTGGCCCAGAGCTATCAGA | 35 |
| DBC-Main-42 | GTCAATCTTGAGATACAAATAGAGCCGCGAATTGA | 35 |
| DBC-Main-43 | CTGACCAAGAACGACCAGAATCCCTCAGTAAGAGC | 35 |
| DBC-Main-44 | AGATGAATTGCCCTTGAATTTCGCCACCAGCAATA | 35 |
| DBC-Main-45 | GGCGCATTGCTCATCGTCATACGGAACCGCCCTTT | 35 |
| DBC-Main-46 | TCATCAACCCAAATATACAGGCACCGGACAGATAG | 35 |
| DBC-Main-47 | TAATAATTTCAGCGTTGAGTAAGCCCCCAATAATA | 35 |
| DBC-Main-48 | AATCTCCTGCTAAAAACAGTTTTCATCGAGAACTG | 35 |
| DBC-Main-49 | AAAGGAGGAATTTTTTTCGGAGCGTCAGCTTATTA | 35 |
| DBC-Main-50 | GGTTTATTCTTTCCAACATGAACAGAATAAACGTA | 35 |
| DBC-Main-51 | GTGAATTAACGATCGAGACTCCAGCACCAAGGTGG | 35 |
| DBC-Main-52 | ATACCGAGACAGCCTAGGATTCACCAATAACGCAA | 35 |
| DBC-Out-n-01 | GTCCAATACTGGTGGCAGAGGGGGTAAT | 28 |
| DBC-Out-n-02 | TTCATTGAATCACTCCGGAATCGTCATA | 28 |
| DBC-Out-n-03 | GACCATAAATCCAGCCCCCTCAAATGCT | 28 |
| DBC-Out-n-04 | CCTGACTATTAAAATAAAAATCAGGTCT | 28 |
| DBC-Out-n-05 | CAACGGAGCCCGAACCTAGTCAGAAGCAA | 29 |
| DBC00-F-n-01 | GATAAAAGTCTGAATCTATCAGGGCGAT | 28 |
| DBC00-F-n-02 | CGAACGATGGCAGACGTCAAAGGGCGA | 27 |
| DBC00-F-n-03 | AAACATCGACCAGTATTAAAGAACGTGG | 28 |
| DBC00-F-n-04 | ATGCGCGTCTGGCCAGTTTGGAACAAGA | 28 |
| DBC00-F-n-05 | TGAATGGCTTCTGAATAGGGTTGAGTG | 27 |
| DBC00-F-n-06 | GCCCGAGCCTGAAAGCGTAAGAATACGCAAATCAAAAGAATA | 42 |
| DBC00-F-n-07 | TCGAATTAATTGTTGAAATCGGCAAAAT | 28 |
| DBC00-F-n-08 | AGGATCCCACACAATCCTGTTTGATGG | 27 |
| DBC00-F-n-09 | TTGTTACGCATAAAGTTTGCCCCAGCAG | 28 |
| DBC00-F-n-10 | GGGCTTATGCCTAAGCAGCAAGCGGTC | 27 |
| DBC00-F-n-11 | GGTTGGTCACATTACCGCCTGGCCCTGA | 28 |
| DBC00-F-n-12 | TTCTCCGACTGCCCGCAACAGCTGATTG | 28 |
| DBC00-F-n-13 | ACGCATTAACCTGTTCTTTTCACCAGTG | 28 |
| DBC00-F-n-14 | ATAGGGGTAATGAAATTGGGCGCCAGGG | 28 |
| DBC00-F-n-15 | AGAAGTTTTGCGAGAGGCGG | 20 |
| DBC00-F-n-16 | AGTAAAATGTTTGTGAGGCTTTTGCAAA | 28 |
| DBC00-F-n-17 | AACGTGCTTTCCAAAACCAAA | 21 |
| DBC00-F-n-18 | TTCGACAGCTAAACGACGAGCACGTAT | 27 |
| DBC00-F-n-19 | AGTATTAAGGGATTGGCGCGTACTATGG | 28 |
| DBC00-F-n-20 | TAATACATACGCCAGCGCTTAATGCGCC | 28 |
| DBC00-F-n-21 | AGATTAGTTTTTATGCGTAACCACCAC | 27 |
| DBC00-F-n-22 | TTAGGAGCCGAGTACAAGTGTAGCGGTC | 28 |
| DBC00-F-n-23 | GGAAGGTTCACGCAGAGCGGGCGCTAGG | 28 |
| DBC00-F-n-24 | CAACAGTCAATACTGGAAGGGAAGAAA | 27 |
| DBC00-F-n-25 | ATATCTGTAACATCGCCGGCGAACGTGG | 28 |
| DBC00-F-n-26 | CAAATATAAGAACTATTTAGAGCTTGAC | 28 |
| DBC00-F-n-27 | AGCATCAGCTGGTAAACCCTAAAGGGA | 27 |
| DBC00-F-n-28 | AGCAGCAATTACCGTGCCGTAAAGCACT | 28 |
| DBC00-F-n-29 | ACAGTGCGGAAAAACAAGTTTTTTGGG | 27 |
| DBC00-F-n-30 | TCAGTATCCTACATTACGTGAACCATCA | 28 |
| DBC02-IN-Ha-01 | GGCCCACTTTGACGCTCAATCCAGAGGTCTTCACACCACACTCCATCTA | 49 |
| DBC02-IN-Ha-02 | AAAACCGATGGATTATTTACATACCACCCTTCACACCACACTCCATCTA | 49 |
| DBC02-IN-Ha-03 | ACTCCAATTCACCAGTCACACGCCATTACTTCACACCACACTCCATCTA | 49 |
| DBC02-IN-Ha-04 | GTCCACTAATAAAAGGGACATAACTGATCTTCACACCACACTCCATCTA | 49 |
| DBC02-IN-Ha-05 | TTGTTCCAACAGAGATAGAACCCTATTACTTCACACCACACTCCATCTA | 49 |
| DBC02-IN-Ha-06 | ATATTTTAAGAAAACAAGCTTTCTCAGGTAGTGGCACAGACACTTCACACCACACTCCATCTA | 63 |
| DBC02-IN-Ha-07 | CCCTTATTGTTTCCTGTGTGACGTAATCCTTCACACCACACTCCATCTA | 49 |
| DBC02-IN-Ha-08 | TGGTTCCATCCGCTCACAATTCCCGGGTCTTCACACCACACTCCATCTA | 49 |
| DBC02-IN-Ha-09 | GCGAAAACATACGAGCCGGAACTCGATACTTCACACCACACTCCATCTA | 49 |
| DBC02-IN-Ha-10 | CACGCTGGTGTAAAGCCTGGGGAGCTACCTTCACACCACACTCCATCTA | 49 |
| DBC02-IN-Ha-11 | GAGAGTTTGAGTGAGCTAACTGTAATGACTTCACACCACACTCCATCTA | 49 |
| DBC02-IN-Ha-12 | CCCTTCAATTGCGTTGCGCTCAACTCTGCTTCACACCACACTCCATCTA | 49 |
| DBC02-IN-Ha-13 | AGACGGGCTTTCCAGTCGGGATCACATACTTCACACCACACTCCATCTA | 49 |
| DBC02-IN-Ha-14 | TGGTTTTCGTGCCAGCTGCATCCTTGAACTTCACACCACACTCCATCTA | 49 |
| DBC02-IN-Ha-15 | TTTGCGTTCGGCCAACGCGCGCGGTGGGCTTCACACCACACTCCATCTA | 49 |
| DBC02-IN-Ha-16 | AAAAAAGGAATTACTCAGACTGGATAGCCTTCACACCACACTCCATCTA | 49 |
| DBC02-IN-Ha-17 | ATAGCGATTACCAGACGACGACACTATCCTTCACACCACACTCCATCTA | 49 |
| DBC02-IN-Ha-18 | ATTAAATCCTTTAATATCGTTAGAATCAGAGCGGGAACTCGTCTTCACACCACACTCCATCTA | 63 |
| DBC02-IN-Ha-19 | TTGCTTTAGGAGGCCGATTAAGACTTTACTTCACACCACACTCCATCTA | 49 |
| DBC02-IN-Ha-20 | GCTACAGTTAGACAGGAACGGTTTGAGGCTTCACACCACACTCCATCTA | 49 |
| DBC02-IN-Ha-21 | ACCCGCCGAATCCTGAGAAGTGAGCCGTCTTCACACCACACTCCATCTA | 49 |
| DBC02-IN-Ha-22 | ACGCTGCAATCAGTGAGGCCACACTAACCTTCACACCACACTCCATCTA | 49 |
| DBC02-IN-Ha-23 | GCGCTGGAAAGAGTCTGTCCATATCTAACTTCACACCACACTCCATCTA | 49 |
| DBC02-IN-Ha-24 | GCGAAAGAATTAACCGTTGTAGTGAAAGCTTCACACCACACTCCATCTA | 49 |
| DBC02-IN-Ha-25 | CGAGAAATCTTTGATTAGTAAGTCAGTTCTTCACACCACACTCCATCTA | 49 |
| DBC02-IN-Ha-26 | GGGGAAAACTTGCCTGAGTAGCAAACCCCTTCACACCACACTCCATCTA | 49 |
| DBC02-IN-Ha-27 | GCCCCCGCAAACTATCGGCCTTCCTTGCCTTCACACCACACTCCATCTA | 49 |
| DBC02-IN-Ha-28 | AAATCGGATATCCAGAACAATAATGAAACTTCACACCACACTCCATCTA | 49 |
| DBC02-IN-Ha-29 | GTCGAGGCCAGCCATTGCAACACACGCTCTTCACACCACACTCCATCTA | 49 |
| DBC02-IN-Ha-30 | CCCAAATCGCTCATGGAAATATAACACCCTTCACACCACACTCCATCTA | 49 |
| DBC04-IN-Ha-01 | GAGGCGGCAATAACGGATTCGCGTCGCTCTTCACACCACACTCCATCTA | 49 |
| DBC04-IN-Ha-02 | AGCAGAAGCTTTGAATACCAAGATAACCCTTCACACCACACTCCATCTA | 49 |
| DBC04-IN-Ha-03 | AAAATACATCGCGCAGAGGCGTAAATCACTTCACACCACACTCCATCTA | 49 |
| DBC04-IN-Ha-04 | AGCCCTACATTTCAATTACCTTTTTTTACTTCACACCACACTCCATCTA | 49 |
| DBC04-IN-Ha-05 | GTCTTTAAGAAGATGATGAAACACAATTCTTCACACCACACTCCATCTA | 49 |
| DBC04-IN-Ha-06 | ATTAATTGTACCGCACTCATCGAGAACTCGGCCAGTGCCAAACTTCACACCACACTCCATCTA | 63 |
| DBC04-IN-Ha-07 | ATGGTCAAGAAGCCAGGGTGGACGTTGTCTTCACACCACACTCCATCTA | 49 |
| DBC04-IN-Ha-08 | ACCGAGCTCTAAGTGGTTGTGACCAGGGCTTCACACCACACTCCATCTA | 49 |
| DBC04-IN-Ha-09 | AAGACGGCGCACGACTTAAGTAGGCGATCTTCACACCACACTCCATCTA | 49 |
| DBC04-IN-Ha-10 | GTGGTGCGTGCTGAATTGTCAACGAAAGCTTCACACCACACTCCATCTA | 49 |
| DBC04-IN-Ha-11 | GTAAACAACAATGTCCCGCCACTTCGCTCTTCACACCACACTCCATCTA | 49 |
| DBC04-IN-Ha-12 | ACCTCCTCCCGCTTCTAATCTAAGGGCGCTTCACACCACACTCCATCTA | 49 |
| DBC04-IN-Ha-13 | AATCATCTCGCCCTGGAGTGACAGGCTGCTTCACACCACACTCCATCTA | 49 |
| DBC04-IN-Ha-14 | TCGGCTGATACCGACAGTGCGGGCAAAGCTTCACACCACACTCCATCTA | 49 |
| DBC04-IN-Ha-15 | CACGAATCATCTGTAAGCAACCGCTTCTCTTCACACCACACTCCATCTA | 49 |
| DBC04-IN-Ha-16 | TTAAACAGTTCATGATACATAACGCCTACTTCACACCACACTCCATCTA | 49 |
| DBC04-IN-Ha-17 | ATAACCCGAGGCATAGTAAGATTCAACTCTTCACACCACACTCCATCTA | 49 |
| DBC04-IN-Ha-18 | AAATAAGGCGTTCAGCGCCCGAACGTTATTAATTTTTGTGATCTTCACACCACACTCCATCTA | 63 |
| DBC04-IN-Ha-19 | CAAACAATGAGTAACATTATCAATGGTTCTTCACACCACACTCCATCTA | 49 |
| DBC04-IN-Ha-20 | ATTTAGAGGAACAAAGAAACCTTCATCTCTTCACACCACACTCCATCTA | 49 |
| DBC04-IN-Ha-21 | CAATAGAGGAGCGGAATTATCATCAAATCTTCACACCACACTCCATCTA | 49 |
| DBC04-IN-Ha-22 | AACTAATCCTGATTATCAGATAAAGAACCTTCACACCACACTCCATCTA | 49 |
| DBC04-IN-Ha-23 | AATATCTATTCATCAATATAAATGCAAACTTCACACCACACTCCATCTA | 49 |
| DBC04-IN-Ha-24 | GAATTGATGTTTGGATTATACTATAACTCTTCACACCACACTCCATCTA | 49 |
| DBC04-IN-Ha-25 | GGCAAATAATGGAAGGGTTAGCTCCGGCCTTCACACCACACTCCATCTA | 49 |
| DBC04-IN-Ha-26 | TCAATCACATATCAAAATTATCTGAGAGCTTCACACCACACTCCATCTA | 49 |
| DBC04-IN-Ha-27 | TGAACCTTAAAACAGAAATAAAAATTTACTTCACACCACACTCCATCTA | 49 |
| DBC04-IN-Ha-28 | AATCTAACGTAGATTTTCAGGCGCTGAGCTTCACACCACACTCCATCTA | 49 |
| DBC04-IN-Ha-29 | GAGAGCCTCAGATGAATATACAAGCGATCTTCACACCACACTCCATCTA | 49 |
| DBC04-IN-Ha-30 | GCCTGCATACCTTTTACATCGCCTTAGACTTCACACCACACTCCATCTA | 49 |
| DBC06-IN-Ha-01 | ATTAATTAGTCCTGAACAAGACGAACCTCTTCACACCACACTCCATCTA | 49 |
| DBC06-IN-Ha-02 | TTGCTTCTATCCCATCCTAATTAAGAACCTTCACACCACACTCCATCTA | 49 |
| DBC06-IN-Ha-03 | ATATATGATGTAGAAACCAATAGAAGGCCTTCACACCACACTCCATCTA | 49 |
| DBC06-IN-Ha-04 | ATGGAAACGGCTGTCTTTCCTAATAGCACTTCACACCACACTCCATCTA | 49 |
| DBC06-IN-Ha-05 | TCATTTGCCAAGAACGGGTATTTAGGAACTTCACACCACACTCCATCTA | 49 |
| DBC06-IN-Ha-06 | TTTTATTAAATATTGAGTAAAGATTCAAAGCAAGCAAGCCGTCTTCACACCACACTCCATCTA | 63 |
| DBC06-IN-Ha-07 | AAAACGATTCATCAACATTAAGCCTTCCCTTCACACCACACTCCATCTA | 49 |
| DBC06-IN-Ha-08 | TTTTCCCCGAGTAACAACCCGTTCAAAACTTCACACCACACTCCATCTA | 49 |
| DBC06-IN-Ha-09 | TAAGTTGTCCGTGGGAACAAATTTTAACCTTCACACCACACTCCATCTA | 49 |
| DBC06-IN-Ha-10 | GGGGATGTTGACCGTAATGGGATTTTGTCTTCACACCACACTCCATCTA | 49 |
| DBC06-IN-Ha-11 | ATTACGCCGTTGGTGTAGATGTTGTTAACTTCACACCACACTCCATCTA | 49 |
| DBC06-IN-Ha-12 | ATCGGTGCGTAACCGTGCATCTAAATTGCTTCACACCACACTCCATCTA | 49 |
| DBC06-IN-Ha-13 | CGCAACTTGAGGGGACGACGAAGATTGTCTTCACACCACACTCCATCTA | 49 |
| DBC06-IN-Ha-14 | CGCCATTGGCCTCAGGAAGATAGAAAAGCTTCACACCACACTCCATCTA | 49 |
| DBC06-IN-Ha-15 | GGTGCCGCAGCCAGCTTTCCGTATGTACCTTCACACCACACTCCATCTA | 49 |
| DBC06-IN-Ha-16 | TTACGAACTAACGGGTGAAAACGAGAATCTTCACACCACACTCCATCTA | 49 |
| DBC06-IN-Ha-17 | AATGCAGAGATTTAGGAATACAGAAAGACTTCACACCACACTCCATCTA | 49 |
| DBC06-IN-Ha-18 | CTGAACAAAGTCGTCAAAATAAGAATAAACACCGGAAACACCCTTCACACCACACTCCATCTA | 63 |
| DBC06-IN-Ha-19 | TGAAATATTACTAGAAAAAGCATTAGACCTTCACACCACACTCCATCTA | 49 |
| DBC06-IN-Ha-20 | TCTGACCGTATCATATGCGTTAACATAACTTCACACCACACTCCATCTA | 49 |
| DBC06-IN-Ha-21 | ATATTTTTTCTTACCAGTATAAGCAGCCCTTCACACCACACTCCATCTA | 49 |
| DBC06-IN-Ha-22 | GCGAGAAGCTCAACAGTAGGGTTAACGTCTTCACACCACACTCCATCTA | 49 |
| DBC06-IN-Ha-23 | TCCAATCGAGAATCGCCATATAAATAAGCTTCACACCACACTCCATCTA | 49 |
| DBC06-IN-Ha-24 | ATATGTACGCCAACATGTAATTCATATTCTTCACACCACACTCCATCTA | 49 |
| DBC06-IN-Ha-25 | TTAGGTTAGGCATTTTCGAGCGCCAGTTCTTCACACCACACTCCATCTA | 49 |
| DBC06-IN-Ha-26 | ACTACCTAAGAGAATATAAAGCGTCTTTCTTCACACCACACTCCATCTA | 49 |
| DBC06-IN-Ha-27 | TCAAAATAAAAGGTAAAGTAATATCTTACTTCACACCACACTCCATCTA | 49 |
| DBC06-IN-Ha-28 | AAGAGTCAGACGACGACAATACCAGCTACTTCACACCACACTCCATCTA | 49 |
| DBC06-IN-Ha-29 | AGCTTAGTGTTCAGCTAATGCAGATTAGCTTCACACCACACTCCATCTA | 49 |
| DBC06-IN-Ha-30 | ATCCTTGCCTGTTTATCAACAGGTTTTGCTTCACACCACACTCCATCTA | 49 |
| DBC08-IN-Ha-01 | CCCGACTAGACACCACGGAATAAGGCCGCTTCACACCACACTCCATCTA | 49 |
| DBC08-IN-Ha-02 | GCGAGGCTTTTGTCACAATCAAGTAGCACTTCACACCACACTCCATCTA | 49 |
| DBC08-IN-Ha-03 | TTATCCGTTCATATGGTTTACTTAGAGCCTTCACACCACACTCCATCTA | 49 |
| DBC08-IN-Ha-04 | AGCAAATAAAGACAAAAGGGCCCGACTTCTTCACACCACACTCCATCTA | 49 |
| DBC08-IN-Ha-05 | TCATTACAACCGATTGAGGGAGAAGGTGCTTCACACCACACTCCATCTA | 49 |
| DBC08-IN-Ha-06 | GAAATTACCTCAGAACCGCCACCCTCACAGTAATGTGTAGCGCTTCACACCACACTCCATCTA | 63 |
| DBC08-IN-Ha-07 | TGTAGCCAAGGGTGAGAAAGGAAATGCACTTCACACCACACTCCATCTA | 49 |
| DBC08-IN-Ha-08 | ATAATTCCAGTCAAATCACCATTTAGAACTTCACACCACACTCCATCTA | 49 |
| DBC08-IN-Ha-09 | CAATAGGATATTCAACCGTTCTCAACGCCTTCACACCACACTCCATCTA | 49 |
| DBC08-IN-Ha-10 | TAAATCATAAATTAATGCCGGATTGCGGCTTCACACCACACTCCATCTA | 49 |
| DBC08-IN-Ha-11 | AATTCGCGCTATTTTTGAGAGCATTATGCTTCACACCACACTCCATCTA | 49 |
| DBC08-IN-Ha-12 | TAAACGTAAGGCTATCAGGTCCTAAATCCTTCACACCACACTCCATCTA | 49 |
| DBC08-IN-Ha-13 | ATAAGCGAGAGTCTGGAGCAATAAAGCCCTTCACACCACACTCCATCTA | 49 |
| DBC08-IN-Ha-14 | CCCCAAAAATCGATGAACGGTAGAATTACTTCACACCACACTCCATCTA | 49 |
| DBC08-IN-Ha-15 | CCCGGTTAAACTAGCATGTCATAAATCACTTCACACCACACTCCATCTA | 49 |
| DBC08-IN-Ha-16 | AGCGGATTGCATCATACGTTAATAAAACCTTCACACCACACTCCATCTA | 49 |
| DBC08-IN-Ha-17 | TTCATCAAACAACATTATTACTGGGAAGCTTCACACCACACTCCATCTA | 49 |
| DBC08-IN-Ha-18 | CAGCATTGACAGGTAGAGAGGGTAATTGAGCGCTAACGCCGCCTTCACACCACACTCCATCTA | 63 |
| DBC08-IN-Ha-19 | GGGAGAAGAGATAACCCACAACACCAGACTTCACACCACACTCCATCTA | 49 |
| DBC08-IN-Ha-20 | AAACAGGGTTAAGCCCAATAAAGCCACCCTTCACACCACACTCCATCTA | 49 |
| DBC08-IN-Ha-21 | TTTACAGAAGAAACAATGAAATCTCAGACTTCACACCACACTCCATCTA | 49 |
| DBC08-IN-Ha-22 | CAAAAATGCTATCTTACCGAAGCCTCCCCTTCACACCACACTCCATCTA | 49 |
| DBC08-IN-Ha-23 | AAACGATTTAAGAAAAGTAAGACCAGAGCTTCACACCACACTCCATCTA | 49 |
| DBC08-IN-Ha-24 | ATTTATCCCGAACAAAGTTACCTCATAACTTCACACCACACTCCATCTA | 49 |
| DBC08-IN-Ha-25 | ACAAAATAACCGAGGAAACGCTTATTAGCTTCACACCACACTCCATCTA | 49 |
| DBC08-IN-Ha-26 | CCAGAGCACGGAATACCCAAAGCATTTTCTTCACACCACACTCCATCTA | 49 |
| DBC08-IN-Ha-27 | CCAACGCGCATGATTAAGACTCACTGTACTTCACACCACACTCCATCTA | 49 |
| DBC08-IN-Ha-28 | CAATTTTCGCAGTATGTTAGCCAAGTTTCTTCACACCACACTCCATCTA | 49 |
| DBC08-IN-Ha-29 | TTGCTATGAAAATACATACATAGTAATCCTTCACACCACACTCCATCTA | 49 |
| DBC08-IN-Ha-30 | AAGCCTTCAACATATAAAAGAGAAACCACTTCACACCACACTCCATCTA | 49 |
| DBC10-IN-Ha-01 | GAAACGTAGCGGGGTTTTGCTTGTAGCACTTCACACCACACTCCATCTA | 49 |
| DBC10-IN-Ha-02 | CCATTACAGGCGGATAAGTGCCCCAGTACTTCACACCACACTCCATCTA | 49 |
| DBC10-IN-Ha-03 | CAGCAAAGGGTTGATATAAGTCCGTAACCTTCACACCACACTCCATCTA | 49 |
| DBC10-IN-Ha-04 | GAGCCATGGAATAGGTGTATCAGCCCAACTTCACACCACACTCCATCTA | 49 |
| DBC10-IN-Ha-05 | AATTATCTCAGGAGGTTTAGTACCTCATCTTCACACCACACTCCATCTA | 49 |
| DBC10-IN-Ha-06 | CATCGGACAAGAACCGCCACCCTTCACACCACACTCCATCTA | 42 |
| DBC10-IN-Ha-07 | ATGCCTGTAAAGTACGGTGTCATGTTTTCTTCACACCACACTCCATCTA | 49 |
| DBC10-IN-Ha-08 | CCCTCATTTCATTCCATATAACATATAACTTCACACCACACTCCATCTA | 49 |
| DBC10-IN-Ha-09 | AAGGATATCCCAATTCTGCGAGGCTTAGCTTCACACCACACTCCATCTA | 49 |
| DBC10-IN-Ha-10 | GAGAAGCGATTTAGTTTGACCAAAGAGGCTTCACACCACACTCCATCTA | 49 |
| DBC10-IN-Ha-11 | ACCCTGTCATTTCGCAAATGGCCTTTAACTTCACACCACACTCCATCTA | 49 |
| DBC10-IN-Ha-12 | GGTTGTACCTGTTTAGCTATAACAGGTCCTTCACACCACACTCCATCTA | 49 |
| DBC10-IN-Ha-13 | TCAGAGTTGGGGCGCGAGCTGCAGACCGCTTCACACCACACTCCATCTA | 49 |
| DBC10-IN-Ha-14 | GCAAAATGGCATCAATTCTACAATTCGACTTCACACCACACTCCATCTA | 49 |
| DBC10-IN-Ha-15 | TACAGGCAGTAGCATTAACATAGACTTCCTTCACACCACACTCCATCTA | 49 |
| DBC10-IN-Ha-16 | CTGATAACTCAAAAAGATTAACTTCACACCACACTCCATCTA | 42 |
| DBC10-IN-Ha-17 | AAAAATCTTATACCAGTCAGGATTTTAACTTCACACCACACTCCATCTA | 49 |
| DBC10-IN-Ha-18 | CGCGACCTTATGCGACGAGGTTGAGGCAGGTCAGACATTGTGCTTCACACCACACTCCATCTA | 63 |
| DBC10-IN-Ha-19 | ACCACCACTTGATATTCACAAGGTTTAACTTCACACCACACTCCATCTA | 49 |
| DBC10-IN-Ha-20 | ACCCTCAAATCCTCATTAAAGGTAGTAACTTCACACCACACTCCATCTA | 49 |
| DBC10-IN-Ha-21 | ACCGCCAGGAAAGCGCAGTCTCGACGAGCTTCACACCACACTCCATCTA | 49 |
| DBC10-IN-Ha-22 | TCAGAGCACCGTTCCAGTAAGTCAGTGACTTCACACCACACTCCATCTA | 49 |
| DBC10-IN-Ha-23 | CCACCACCATGGCTTTTGATGCAACGTACTTCACACCACACTCCATCTA | 49 |
| DBC10-IN-Ha-24 | TCAAAATAGTGTACTGGTAATACGGATACTTCACACCACACTCCATCTA | 49 |
| DBC10-IN-Ha-25 | CGTTTGCACGGGGTCAGTGCCGAGTGAGCTTCACACCACACTCCATCTA | 49 |
| DBC10-IN-Ha-26 | CGGTCATACAGTGCCCGTATACAACTTTCTTCACACCACACTCCATCTA | 49 |
| DBC10-IN-Ha-27 | GCGCGTTAATGCCCCCTGCCTACTGTATCTTCACACCACACTCCATCTA | 49 |
| DBC10-IN-Ha-28 | GCCTTTAACCTATTATTCTGAAGACGTTCTTCACACCACACTCCATCTA | 49 |
| DBC10-IN-Ha-29 | AGTAGCGAAGTATTAAGAGGCTTAAAGTCTTCACACCACACTCCATCTA | 49 |
| DBC10-IN-Ha-30 | TCGATAGCTCAAGAGAAGGATCTCATAGCTTCACACCACACTCCATCTA | 49 |
| DBC11-D-n-01 | TTCCACATAGTTGCGCCGACA | 21 |
| DBC11-D-n-02 | CAAACTACAACCATCGCCCACG | 22 |
| DBC11-D-n-03 | ACTGAGTGATATATTCGGTCG | 21 |
| DBC11-D-n-04 | TAGGAACTTGCAGGGAGTTAA | 21 |
| DBC11-D-n-05 | TTTCAGGTTTTGCGGGATCGTC | 22 |
| DBC11-D-n-06 | CTCAGAGGCAGCGAAAGACAG | 21 |
| DBC11-D-n-07 | AAATATGACGAGGGTAGCAAC | 21 |
| DBC11-D-n-08 | TGCTGTAGAGGCTTTGAGGACT | 22 |
| DBC11-D-n-09 | AGCTTAATTTTCATGAGGAAG | 21 |
| DBC11-D-n-10 | TCATTTTTAAACGGGTAAAATA | 22 |
| DBC11-D-n-11 | TTGCTCCCCACTACGAAGGCA | 21 |
| DBC11-D-n-12 | AGGATTAAAAACGAAAGAGGC | 21 |
| DBC11-D-n-13 | GAAGCATACACTAAAACACTC | 21 |
| DBC11-D-n-14 | GCTTCAAACCCCCAGCGATTA | 21 |
| DBC11-D-n-15 | AAATATCCGCGAAACAAAGTA | 21 |
| DBC11-D-n-16 | GAGGAAGATTTGTATCATCGC | 21 |
| DBC11-D-n-17 | GAACTGGATTGTGTCGAAATC | 21 |
| DBC11-D-n-18 | AATTACCTGCTCCATGTTACTT | 22 |
| DBC11-D-n-19 | TTTCAACACGAGGCGCAGACG | 21 |
| DBC11-D-n-20 | ATTGGGCATAAGGGAACCGAA | 21 |
| DBC11-D-n-21 | AAACACCACTTTGAAAGAGGAC | 22 |
| DBC11-D-n-22 | ATAAGGCCGGTGTACAGACCA | 21 |
| DBC11-D-n-23 | ACAAAGCAGGCTGGCTGACCT | 21 |
| DBC11-D-n-24 | TTCATTAGAGTAATCTTGACAA | 22 |
| DBC11-D-n-25 | GACAACTAAAGGAATTGCGAA | 21 |
| DBC11-D-n-26 | CAACAGTTTTTTCACGTTGAA | 21 |
| DBC11-D-n-27 | GGGATTTAAAAAAAAGGCTCCA | 22 |
| DBC11-D-n-28 | AGTAAATCCTTTAATTGTATC | 21 |
| DBC11-D-n-29 | TTTGTCGCAGCTTGCTTTCGAG | 22 |
| DBC11-D-n-30 | TTAGCGTTCTTAAACAGCTTG | 21 |
| DNA Soccer-ball Captor | | |
| DSC-Vertex-001 | GAGACCATACAGGCATTTTAGGCAA | 25 |
| DSC-Vertex-002 | AGACGGGCTCAAAAGGGTGAGAATTTTAGGCCG | 33 |
| DSC-Vertex-003 | AGAATTGCCTGAGTATTTTATGTGT | 25 |
| DSC-Vertex-004 | AGTAGATATTCAACCTTTTGTTCTA | 25 |
| DSC-Vertex-005 | TTTTTCATCAATTCTTTTTACTAAT | 25 |
| DSC-Vertex-006 | CTGACGCATAATGCCGGAGAGGGTTTTTAGCTA | 33 |
| DSC-Vertex-007 | ATTTGAGGTCATTGCTTTTCTGAGA | 25 |
| DSC-Vertex-008 | TAATCGTTTAGCTATTTTTATTTTC | 25 |
| DSC-Vertex-009 | CGCCATTCAACAAGAGAATCGATTTTTGAACGG | 33 |
| DSC-Vertex-010 | TACATTATGTACCCCTTTTGGTTGA | 25 |
| DSC-Vertex-011 | CCAAAAACAGATTTAGTTTGACCTTTTATTAGA | 33 |
| DSC-Vertex-012 | TAATCGTTGATTCCCTTTTAATTCT | 25 |
| DSC-Vertex-013 | TAAATACTTTTGCGGTTTTGAGAAG | 25 |
| DSC-Vertex-014 | AGAACCCTCAGAGCATTTTTAAAGC | 25 |
| DSC-Vertex-015 | TGTTTAGAAACGCAAGGATAAAATTTTATTTTT | 33 |
| DSC-Vertex-016 | CTTTTTCACCGCCTGTTTTGCCCTG | 25 |
| DSC-Vertex-017 | ATCATGGTTTGGGCGCCAGGGTGTTTTGTTTTT | 33 |
| DSC-Vertex-018 | AGAGAACGCGCGGGGTTTTAGAGGC | 25 |
| DSC-Vertex-019 | GGCCTTCTCCGAACTTTTTCTGACC | 25 |
| DSC-Vertex-020 | AAAACGACGTCGGTGGGCACGAATTTTTATAGG | 33 |
| DSC-Vertex-021 | TCCTGGCGGCCCTGCTTTTCATCTG | 25 |
| DSC-Vertex-022 | GCAAAGGGAAGGGCGTTTTATCGGT | 25 |
| DSC-Vertex-023 | TTCGCGTCGCTTCTGGTGCCGGATTTTAACCAG | 33 |
| DSC-Vertex-024 | GCGGGCGCACTCCAGTTTTCCAGCT | 25 |
| DSC-Vertex-025 | GTCTGAGATTGTATATTTTAGCAAA | 25 |
| DSC-Vertex-026 | AGAAGCAAAATATGCAACTAAAGTTTTTACGGT | 33 |
| DSC-Vertex-027 | TATTTATAATGCTGTTTTTAGCTCA | 25 |
| DSC-Vertex-028 | GGTAAACTGCGGAATTTTTCGTCAT | 25 |
| DSC-Vertex-029 | AAGAATAGAAAAGAAGTTTTGCCTTTTAGAGGG | 33 |
| DSC-Vertex-030 | AAATAAAACCAAAATTTTTAGCGAG | 25 |
| DSC-Vertex-031 | GAACAGCCCCCGATTTTTTTAGAGC | 25 |
| DSC-Vertex-032 | CCTTAGTGTTGTTCCTTTTAGTTTG | 25 |
| DSC-Vertex-033 | TTGACCGAAATCGGCTTTTAAAATC | 25 |
| DSC-Vertex-034 | TGCCATGGTTTGCCCTTTTCAGCAG | 25 |
| DSC-Vertex-035 | GAAAGGTCGGGAAACTTTTCTGTCG | 25 |
| DSC-Vertex-036 | GCGAACGAGAAAGGATTTTAGGGAA | 25 |
| DSC-Vertex-037 | CTCACAGGAACGGTATTTTCGCCAG | 25 |
| DSC-Vertex-038 | GCTCGGTGAAATTGTTTTTTATCCG | 25 |
| DSC-Vertex-039 | AATCCGATCCCCGGGTTTTTACCGA | 25 |
| DSC-Vertex-040 | TGGAGGGCTTAAGCTTTTTACGTGG | 25 |
| DSC-Vertex-041 | AAAGACTATTTACGCTTTTTCGCCC | 25 |
| DSC-Vertex-042 | TGCTTGTGAGGCCACTTTTCGAGTA | 25 |
| DSC-Vertex-043 | GGTGGGGATTATTTATTTTCATTGG | 25 |
| DSC-Vertex-044 | GTCACTCTCAGGAGATTTTAGCCAG | 25 |
| DSC-Vertex-045 | CAGATCGCCAGGGTTTTTTTTCCCA | 25 |
| DSC-Vertex-046 | GACGATGGCGAAAGGTTTTGGGATG | 25 |
| DSC-Vertex-047 | GGCCAGCCAGTTTGATTTTGGGGAC | 25 |
| DSC-Vertex-048 | TGCTGATAAAAGGGATTTTCATTCT | 25 |
| DSC-Vertex-049 | AAATGCCACGCTGAGTTTTAGCCAG | 25 |
| DSC-Vertex-050 | CAGCATTAACCAATATTTTGGAACG | 25 |
| DSC-Vertex-051 | CCATCAGCTTTCATCTTTTAACATT | 25 |
| DSC-Vertex-052 | AAATTCCTTGCTGAATTTTCCTCAA | 25 |
| DSC-Vertex-053 | ATATCAGAGGTCATTTTTTTTTGCG | 25 |
| DSC-Vertex-054 | GATGGTGTTAAAATTTTTTCGCATT | 25 |
| DSC-Vertex-055 | CCGAAACGTTATTAATTTTTTTTAA | 25 |
| DSC-Vertex-056 | GACTAAAGATTAAGATTTTGGAAGC | 25 |
| DSC-Vertex-057 | AAGTTATCAGGTCTTTTTTTACCCT | 25 |
| DSC-Vertex-058 | AACCCGCTTTAAACATTTTGTTCAG | 25 |
| DSC-Vertex-059 | CAGAAAGAGCAACACTTTTTATCAT | 25 |
| DSC-Vertex-060 | AAAACGGAACAAAGATTTTAACCAC | 25 |
| DSC-Vertex-061 | GCCAATCCTGATTATTTTTCAGATG | 25 |
| DSC-Vertex-062 | GTCTATCACAACTAATGCAGATATTTTCATAAC | 33 |
| DSC-Vertex-063 | ATGGCCAGTTGAGATTTTTTTAGGA | 25 |
| DSC-Vertex-064 | TTTCATCGTTTGGGGTCGAGGTGTTTTCCGTAA | 33 |
| DSC-Vertex-065 | AGGGCCGTGAACCATTTTTCACCCA | 25 |
| DSC-Vertex-066 | AGCACTGGACTCCAATTTTCGTCAA | 25 |
| DSC-Vertex-067 | GGTCAAATGCGCCGCTTTTTACAGG | 25 |
| DSC-Vertex-068 | GCGCGCTAACTCACATTTTTTAATT | 25 |
| DSC-Vertex-069 | GCGTTCGCTGGCAAGTTTTTGTAGC | 25 |
| DSC-Vertex-070 | TAAGAGAAAATCAGAGCGGGAGCTTTTTAAACA | 33 |
| DSC-Vertex-071 | AAAGCCACGTATAACTTTTGTGCTT | 25 |
| DSC-Vertex-072 | GGAGGCGGAAGCATATTTTAAGTGT | 25 |
| DSC-Vertex-073 | TTCTTAGAACTCAAATTTTCTATCG | 25 |
| DSC-Vertex-074 | GCCTTAACCTTATGATTTTCAATGT | 25 |
| DSC-Vertex-075 | CCCGCACCGTTGTAGTTTTCAATAC | 25 |
| DSC-Vertex-076 | AATATATTAAACGCTCATGGAAATTTTTACCTA | 33 |
| DSC-Vertex-077 | TTAAGATTACCGCCATTTTGCCATT | 25 |
| DSC-Vertex-078 | CATTTAATTCATGCGTTTTCACGAC | 25 |
| DSC-Vertex-079 | TACGTCTTTAATGCGTTTTCGAACT | 25 |
| DSC-Vertex-080 | GATGGCCTGAAAGCGTTTTTAAGAA | 25 |
| DSC-Vertex-081 | GATAGAGGTCACGTTTTTTGGTGTA | 25 |
| DSC-Vertex-082 | CAGTAGATTCTCCGTTTTTGGGAAC | 25 |
| DSC-Vertex-083 | ATAAATCAATAAAACAGAGGTGATTTTGGCGGT | 33 |
| DSC-Vertex-084 | AAACGAATACCGAACTTTTGAACCA | 25 |
| DSC-Vertex-085 | CAGTTTTTAGGAGCATTTTCTAACA | 25 |
| DSC-Vertex-086 | AGTACTCAGTTGGCATTTTAATCAA | 25 |
| DSC-Vertex-087 | ACTAACAGGTCAGGATTTTTTAGAG | 25 |
| DSC-Vertex-088 | CAACTAATTCGAGCTTTTTTCAAAG | 25 |
| DSC-Vertex-089 | CGAACTAATACATTTTTTTGAGGAT | 25 |
| DSC-Vertex-090 | AGAAATAATAGACTTTACAAACATTTTATTCGA | 33 |
| DSC-Vertex-091 | ATCAGCAGAGAGAATTTTTAACATA | 25 |
| DSC-Vertex-092 | AAAACCTCATCGAGATTTTACAAGC | 25 |
| DSC-Vertex-093 | AAGCCGCCCAATAGCTTTTAAGCAA | 25 |
| DSC-Vertex-094 | GTAACCACTGTCTTTCCTTATCATTTTTTCCAA | 33 |
| DSC-Vertex-095 | GAACGGAATTAACTGTTTTAACACC | 25 |
| DSC-Vertex-096 | CTGAACATGTAGAAATTTTCCAATC | 25 |
| DSC-Vertex-097 | GTCCATACATGGCTTTTTTTTGATG | 25 |
| DSC-Vertex-098 | ATACATTTAGGCAGATTTTGGCATT | 25 |
| DSC-Vertex-099 | TTCGAAAGGTAAAGTTTTTAATTCT | 25 |
| DSC-Vertex-100 | AATAACATGTAGGGCTTAATTGATTTTGAATCG | 33 |
| DSC-Vertex-101 | CCATATTAACGGGGTTTTTCAGTGC | 25 |
| DSC-Vertex-102 | CTTGATACCAGTATATTTTAAGCCA | 25 |
| DSC-Vertex-103 | TGGTTAGTACAAACTTTTTACAACG | 25 |
| DSC-Vertex-104 | CCTGTAAGACAAAGATTTTACGCGA | 25 |
| DSC-Vertex-105 | GAAAATCTGACCTAATTTTATTTAA | 25 |
| DSC-Vertex-106 | CAATATTTGGTTGGGTTATATAATTTTCTATAT | 33 |
| DSC-Vertex-107 | GTAAAATAGTTAGCGTTTTTAACGA | 25 |
| DSC-Vertex-108 | TCTAAAGACTACCTTTTTTTTTAAC | 25 |
| DSC-Vertex-109 | ATCGTTCGTCACCCTTTTTCAGCAG | 25 |
| DSC-Vertex-110 | ATGGAAACCTTGCTTTTTTCTGTAA | 25 |
| DSC-Vertex-111 | CGAAATGAATTACCTTTTTTTTTTA | 25 |
| DSC-Vertex-112 | TTGAGGAAAAACAAACATCAAGATTTTAAACAA | 33 |
| DSC-Vertex-113 | CTTTGAATTACCTGATTTTGCAAAA | 25 |
| DSC-Vertex-114 | AATTAGCAACGGCTATTTTCAGAGG | 25 |
| DSC-Vertex-115 | GATGATCATCAAGAGTTTTTAATCT | 25 |
| DSC-Vertex-116 | TTGCATTCAGGTTTATTTTACGTCA | 25 |
| DSC-Vertex-117 | TGACAACCATATCAATTTTAATTAT | 25 |
| DSC-Vertex-118 | ACATTTTGTTTGGATTTTTTATACT | 25 |
| DSC-Vertex-119 | GCTGCACGAACTAACTTTTGGAACA | 25 |
| DSC-Vertex-120 | TCTGAAAATCAACGTTTTTAACAAA | 25 |
| DSC-Vertex-121 | AATAACGCTAATATCTTTTAGAGAG | 25 |
| DSC-Vertex-122 | ATAACGCAAGAAACATTTTATGAAA | 25 |
| DSC-Vertex-123 | TAGCAAGTCCTGAACTTTTAAGAAA | 25 |
| DSC-Vertex-124 | ACGCGTTTTTAAGAATTTTAAGTAA | 25 |
| DSC-Vertex-125 | GCAGATGGAAAGCGCTTTTAGTCTC | 25 |
| DSC-Vertex-126 | TGAATGTTCAGCTAATTTTTGCAGA | 25 |
| DSC-Vertex-127 | TATTTAAGAGGCTGATTTTGACTCC | 25 |
| DSC-Vertex-128 | AGTATGTTAATGCCCTTTTCCTGCC | 25 |
| DSC-Vertex-129 | TCAAGTAGAAAAAGCTTTTCTGTTT | 25 |
| DSC-Vertex-130 | GTACCAATAAGGCGTTTTTTAAATA | 25 |
| DSC-Vertex-131 | GGCGGCCCAATAGGATTTTACCCAT | 25 |
| DSC-Vertex-132 | AGAATGTTTTGCTCATTTTGTACCA | 25 |
| DSC-Vertex-133 | TCTGTCAGCGGAGTGTTTTAGAATA | 25 |
| DSC-Vertex-134 | GAATTGTTAGTAAATTTTTGAATTT | 25 |
| DSC-Vertex-135 | GAAAGGAGAAGAGTCTTTTAATAGT | 25 |
| DSC-Vertex-136 | GGGAGTAGAATCCTTTTTTGAAAAC | 25 |
| DSC-Vertex-137 | GTTGAGTCGCTGAGGTTTTCTTGCA | 25 |
| DSC-Vertex-138 | ATAGCATAATAATTTTTTTTTTCAC | 25 |
| DSC-Vertex-139 | ACGGGCCTAAAACGATTTTAAGAGG | 25 |
| DSC-Vertex-140 | TCGCGAGGAAGTTTCTTTTCATTAA | 25 |
| DSC-Vertex-141 | CAAAAATACCAAGTTTTTTACAAAA | 25 |
| DSC-Vertex-142 | GACCATACATCGGGATTTTGAAACA | 25 |
| DSC-Vertex-143 | TATACAGATGAACGGTTTTTGTACA | 25 |
| DSC-Vertex-144 | ATAACTTTGACCCCCTTTTAGCGAT | 25 |
| DSC-Vertex-145 | TGGGACCCTGACGAGTTTTAAACAC | 25 |
| DSC-Vertex-146 | CAGAATTCAACTTTATTTTATCATT | 25 |
| DSC-Vertex-147 | GTGAAATACCAGTCATTTTGGACGT | 25 |
| DSC-Vertex-148 | GTCAATTCTAAGAACTTTTGCGAGG | 25 |
| DSC-Vertex-149 | TTTTGCGATTTTTTGTTTTTTTAAC | 25 |
| DSC-Vertex-150 | TGCGATTTAACCTCCCGACTTGCTTTTGGGAGG | 33 |
| DSC-Vertex-151 | ATTGAGTTGTTACAAAATAAACATTTTGCCATA | 33 |
| DSC-Vertex-152 | GCTACGCGTCTTTCCTTTTAGAGCC | 25 |
| DSC-Vertex-153 | TTATTTGCTATTTTGTTTTCACCCA | 25 |
| DSC-Vertex-154 | ATTATTCTATTGGCCTTGATATTTTTTCACAAA | 33 |
| DSC-Vertex-155 | CAAATAGGAAACCGATTTTGGAAAC | 25 |
| DSC-Vertex-156 | GCAATGACAGGAGGTTTTTTGAGGC | 25 |
| DSC-Vertex-157 | TTGCTAAAGAACCGCCACCCTCATTTTGAGCCA | 33 |
| DSC-Vertex-158 | GGAATCGCCACCCTCTTTTAGAACC | 25 |
| DSC-Vertex-159 | CCACCGATATAAGTATTTTTAGCCC | 25 |
| DSC-Vertex-160 | GTAATGCCCGCCGACAATGACAATTTTCAACCA | 33 |
| DSC-Vertex-161 | TCGCCCAAAAGGAGCTTTTCTTTAA | 25 |
| DSC-Vertex-162 | TTGTACTTAAACAGCTTTTTTGATA | 25 |
| DSC-Vertex-163 | AAATTGGGGACGGTCAATCATAATTTTGGGAAC | 33 |
| DSC-Vertex-164 | CGCCTATGTTACTTATTTTGCCGGA | 25 |
| DSC-Vertex-165 | CGAACCGGAGATTTGTTTTTATCAT | 25 |
| DSC-Vertex-166 | CCTGAATCTCACCAGTAGCACCATTTTTTACCA | 33 |
| DSC-Vertex-167 | TTCATCCATTTGGGATTTTATTAGA | 25 |
| DSC-Vertex-168 | TTAGCATATTGACGGTTTTAAATTA | 25 |
| DSC-Vertex-169 | CCACCACTGGCATGATTTTTTAAGA | 25 |
| DSC-Vertex-170 | GAAAAAGAGCCGCCATTTTCCAGAA | 25 |
| DSC-Vertex-171 | AATTATCACGCAGTATGTTAGCATTTTAACGTA | 33 |
| DSC-Vertex-172 | CCGCCATATAAAAGATTTTAACGCA | 25 |
| DSC-Vertex-173 | CACCGTACTCAGAGCCGCCACCCTTTTTCAGAA | 33 |
| DSC-Vertex-174 | AAGACCAGAGCCACCTTTTACCGGA | 25 |
| DSC-Vertex-175 | CTTTTGTCACAATCATTTTATAGAA | 25 |
| DSC-Vertex-176 | TCAGCTTGCCCCTTATTAGCGTTTTTTTGCCAT | 33 |
| DSC-Vertex-177 | AATTCTTTTCATCGGTTTTCATTTT | 25 |
| DSC-Vertex-178 | TCAACGAAACCATCGTTTTATAGCA | 25 |
| DSC-Vertex-179 | TGCCTGACAAAAGGGTTTTCGACAT | 25 |
| DSC-Vertex-180 | GTGTCGAACAGTAGCGACAGAATTTTTCAAGTT | 33 |
| DSC-In-001 | ACATCCAATAAATAGTCAAATCACCCTTCACACCACACTCCATCTA | 46 |
| DSC-In-002 | GCTGAAAAGGTGGGAGAGATCTACACTTCACACCACACTCCATCTA | 46 |
| DSC-In-003 | TGGTCAATAACCTGTAAAACTAGCACTTCACACCACACTCCATCTA | 46 |
| DSC-In-004 | GCGAACGAGTATTATGACTTCACACCACACTCCATCTA | 38 |
| DSC-In-005 | TTAAGCAATAAAGCCTCATATATTTCTTCACACCACACTCCATCTA | 46 |
| DSC-In-006 | AGGTAAAGATAACAGCTCTTCACACCACACTCCATCTA | 38 |
| DSC-In-007 | GCTGATAAATTTTCACACTTCACACCACACTCCATCTA | 38 |
| DSC-In-008 | GTCTGGAGCAAGGCTGCCTTCACACCACACTCCATCTA | 38 |
| DSC-In-009 | ATTCCATATAACAAGAAAAGCCCCACTTCACACCACACTCCATCTA | 46 |
| DSC-In-010 | CCTTTATTTCCTGGATACTTCACACCACACTCCATCTA | 38 |
| DSC-In-011 | AGGCTTTTGCCCCGAGACTTCACACCACACTCCATCTA | 38 |
| DSC-In-012 | AAGCGGTCCACGCGCTGCATTAATGCTTCACACCACACTCCATCTA | 46 |
| DSC-In-013 | GGTTTGCGTACATAGCTCTTCACACCACACTCCATCTA | 38 |
| DSC-In-014 | AATGAGTAAACAGTGACTCTATGATCTTCACACCACACTCCATCTA | 46 |
| DSC-In-015 | TAAGCAACTCGGCCAGTCTTCACACCACACTCCATCTA | 38 |
| DSC-In-016 | CTATTACGCCAGCCAGTATCGGCCTCTTCACACCACACTCCATCTA | 46 |
| DSC-In-017 | TTCCGGCACCTGGCCTTCTTCACACCACACTCCATCTA | 38 |
| DSC-In-018 | AACGTTAATATTTCTTAGAGCTTAACTTCACACCACACTCCATCTA | 46 |
| DSC-In-019 | ACATGTTTTAAGCGGATCTTCACACCACACTCCATCTA | 38 |
| DSC-In-020 | ATCCCCCTCAAATTCGTTTACCAGACTTCACACCACACTCCATCTA | 46 |
| DSC-In-021 | CCGGCGAACGTGGAATCCTGTTTGACTTCACACCACACTCCATCTA | 46 |
| DSC-In-022 | GTTTTTATAATCAGTTACCTCGATACTTCACACCACACTCCATCTA | 46 |
| DSC-In-023 | CACACGACCAGTACAAGGCGATTAACTTCACACCACACTCCATCTA | 46 |
| DSC-In-024 | ATCTAAAGCATCATTTGTTAAATCACTTCACACCACACTCCATCTA | 46 |
| DSC-In-025 | ATTATCATTTTGCGAGAATGACCATCTTCACACCACACTCCATCTA | 46 |
| DSC-In-026 | AACCCTAAAGGGAAGAGTCCACTATCTTCACACCACACTCCATCTA | 46 |
| DSC-In-027 | GCGGGCGCTAGGGGCGCTCACTGCCCTTCACACCACACTCCATCTA | 46 |
| DSC-In-028 | AGGGATTTTAGACAATTCCACACAACTTCACACCACACTCCATCTA | 46 |
| DSC-In-029 | ATCACGCAAATTACAAAATAACCCCCTTCACACCACACTCCATCTA | 46 |
| DSC-In-030 | AATCGTCTGAAATATGTTCTTCTAACTTCACACCACACTCCATCTA | 46 |
| DSC-In-031 | AGAACCCTTCTGAGCGCATCGTAACCTTCACACCACACTCCATCTA | 46 |
| DSC-In-032 | GCCTGCAACAGTGTGAGCGAGTAACCTTCACACCACACTCCATCTA | 46 |
| DSC-In-033 | AATCAATATCTGGCTTTAATTGCTCCTTCACACCACACTCCATCTA | 46 |
| DSC-In-034 | ATCCTTTGCCCGAAGACTTCAAATACTTCACACCACACTCCATCTA | 46 |
| DSC-In-035 | ATTATCATCATATAAGGAATTACGACTTCACACCACACTCCATCTA | 46 |
| DSC-In-036 | ATACCACATTGGGCGATCTTCACACCACACTCCATCTA | 38 |
| DSC-In-037 | CCTAATGAGTGAGTACTATGGTTGCCTTCACACCACACTCCATCTA | 46 |
| DSC-In-038 | GTGCTGAATTGTCGCTGGTAATATCCTTCACACCACACTCCATCTA | 46 |
| DSC-In-039 | ACCGTAATGGGATCCCTAAAACATCCTTCACACCACACTCCATCTA | 46 |
| DSC-In-040 | AAGCAAACTCCAATAGATTAGAGCCCTTCACACCACACTCCATCTA | 46 |
| DSC-In-041 | AATCAAGTTTTAGGAATCTTCACACCACACTCCATCTA | 38 |
| DSC-In-042 | AATAATCGGCCACACCCCTTCACACCACACTCCATCTA | 38 |
| DSC-In-043 | TCCTCGTTAGTATAAAGCTTCACACCACACTCCATCTA | 38 |
| DSC-In-044 | ACGCTCAACACACTTGCCTTCACACCACACTCCATCTA | 38 |
| DSC-In-045 | GCAACAGGAATTAGTTACTTCACACCACACTCCATCTA | 38 |
| DSC-In-046 | CTCCGGCTTATTGAATGCTTCACACCACACTCCATCTA | 38 |
| DSC-In-047 | CCAGCAGAAGATATATGCTTCACACCACACTCCATCTA | 38 |
| DSC-In-048 | GAAGATGATGGGTTATCCTTCACACCACACTCCATCTA | 38 |
| DSC-In-049 | TTAGAAGTATAGAAATTCTTCACACCACACTCCATCTA | 38 |
| DSC-In-050 | AATATAATCCTGAATTACAGGTAGACTTCACACCACACTCCATCTA | 46 |
| DSC-In-051 | ACCAAGTACCGCAAGGGAAGCGCATCTTCACACCACACTCCATCTA | 46 |
| DSC-In-052 | CGCCAACATGTAAGGAGTGTACTGGCTTCACACCACACTCCATCTA | 46 |
| DSC-In-053 | CAAATCCAATCGCAGCATTCCACAGCTTCACACCACACTCCATCTA | 46 |
| DSC-In-054 | TAACAATTTCATTGACAGCATCGGACTTCACACCACACTCCATCTA | 46 |
| DSC-In-055 | AGGGTTAGAACCTAGAACCGGATATCTTCACACCACACTCCATCTA | 46 |
| DSC-In-056 | ATAGCAGCCTTTAATATAGAAGGCTCTTCACACCACACTCCATCTA | 46 |
| DSC-In-057 | GAGGGTAATTGAGTATCCCATCCTACTTCACACCACACTCCATCTA | 46 |
| DSC-In-058 | CCAGTAAGCGTCAGACGACGACAATCTTCACACCACACTCCATCTA | 46 |
| DSC-In-059 | GCCCGTATAAACACATATGCGTTATCTTCACACCACACTCCATCTA | 46 |
| DSC-In-060 | GAGTTTCGTCACCTGAAATACCGACCTTCACACCACACTCCATCTA | 46 |
| DSC-In-061 | CGTCTTTCCAGACTATCAAAATCATCTTCACACCACACTCCATCTA | 46 |
| DSC-In-062 | CGCTTTTGCGGGACGCTATTAATTACTTCACACCACACTCCATCTA | 46 |
| DSC-In-063 | AGACTTTTTCATGCAGAGGCGAATTCTTCACACCACACTCCATCTA | 46 |
| DSC-In-064 | GGCTGGCTGACCTATATACAGTAACCTTCACACCACACTCCATCTA | 46 |
| DSC-In-065 | TGAATAAGGCTTGAGAAAAATCTACCTTCACACCACACTCCATCTA | 46 |
| DSC-In-066 | CTTACCGAAGCCCCCTGTTTATCAACTTCACACCACACTCCATCTA | 46 |
| DSC-In-067 | TAGGATTAGCGGGAAACACCGGAATCTTCACACCACACTCCATCTA | 46 |
| DSC-In-068 | AAAGGAATTGCGAGATAGCTTAGATCTTCACACCACACTCCATCTA | 46 |
| DSC-In-069 | TAAAACACTCATCGGATTCGCCTGACTTCACACCACACTCCATCTA | 46 |
| DSC-In-070 | CGTTTTAGCGTAAGAACCTTCACACCACACTCCATCTA | 38 |
| DSC-In-071 | TCCAAATAAGAAAAAGCCTTAAATCCTTCACACCACACTCCATCTA | 46 |
| DSC-In-072 | TAATTTGCCAAAGCCCACTTCACACCACACTCCATCTA | 38 |
| DSC-In-073 | ATTAAAGCCAGAATAGCCGAACAAACTTCACACCACACTCCATCTA | 46 |
| DSC-In-074 | AGGTCAGACGGAAACATCTTCACACCACACTCCATCTA | 38 |
| DSC-In-075 | CAGGGATAGCAAGATAAGTGCCGTCCTTCACACCACACTCCATCTA | 46 |
| DSC-In-076 | GCCACCCTCACAACTTTCTTCACACCACACTCCATCTA | 38 |
| DSC-In-077 | ACCGATATATTCGAAATCTCCAAAACTTCACACCACACTCCATCTA | 46 |
| DSC-In-078 | CCGATAGTTGACTACGACTTCACACCACACTCCATCTA | 38 |
| DSC-In-079 | TTTGAAAGAGGACCAAGCGCGAAACCTTCACACCACACTCCATCTA | 46 |
| DSC-In-080 | ACGAGGCGCACTTGAGACTTCACACCACACTCCATCTA | 38 |
| DSC-In-081 | GCCAGCAAAATTACCAACTTCACACCACACTCCATCTA | 38 |
| DSC-In-082 | AATACCCAAAAGAACCAGAGCCGCCCTTCACACCACACTCCATCTA | 46 |
| DSC-In-083 | ACCGCCTCCCTCAGGAGCTTCACACCACACTCCATCTA | 38 |
| DSC-In-084 | CGGTCATAGCCTTTCGACTTCACACCACACTCCATCTA | 38 |
| DSC-In-085 | GCACCGTAATATCCGCGCTTCACACCACACTCCATCTA | 38 |
| DSC-In-086 | GGAGGGAAGGTAAAAGGCCGGAAACCTTCACACCACACTCCATCTA | 46 |
| DSC-In-087 | CTCCTTATTACCGTCACCTTCACACCACACTCCATCTA | 38 |
| DSC-In-088 | TAAAGGTGGCAACACCCTCAGAGCCCTTCACACCACACTCCATCTA | 46 |
| DSC-In-089 | ATAAGTTTATTTTCATAATCAAAATCTTCACACCACACTCCATCTA | 46 |
| DSC-In-090 | TACCAGCGCCAAATTAGCGTCAGACCTTCACACCACACTCCATCTA | 46 |
| DSC-Out-n-001 | ATCAATATGTAGCATTA | 18 |
| DSC-Out-n-002 | AAGGCTATCGGGCGCGA | 18 |
| DSC-Out-n-003 | TGTCAATCATTCGCAAA | 18 |
| DSC-Out-n-004 | CCCTGTAATCGGTTGTA | 18 |
| DSC-Out-n-005 | TAAATGCAATAGCAAAA | 18 |
| DSC-Out-n-006 | GATTGCCCTCACCAGTG | 18 |
| DSC-Out-n-007 | TAAATCATTTGAATCGG | 18 |
| DSC-Out-n-008 | GCAACTGTTGCGCCATT | 18 |
| DSC-Out-n-009 | AAAACAGGAGAAGTTTC | 18 |
| DSC-Out-n-010 | GCGTCCAATTAGTAAAA | 18 |
| DSC-Out-n-011 | TAGGGTTGATAAATCAA | 18 |
| DSC-Out-n-012 | AATCGGCCAGTTGCAGC | 18 |
| DSC-Out-n-013 | GTTTCCTGTAATTCGTA | 18 |
| DSC-Out-n-014 | ACCGACAGTGTTGGTGT | 18 |
| DSC-Out-n-015 | GCCAAGCTTGACGTTGT | 18 |
| DSC-Out-n-016 | CAGGAAGATCCTCTTCG | 18 |
| DSC-Out-n-017 | CCTGTAGCCAAAAATAA | 18 |
| DSC-Out-n-018 | TTGCTGAATAAATTGTA | 18 |
| DSC-Out-n-019 | TGCATCAAATTATAGTC | 18 |
| DSC-Out-n-020 | CGACGATAATTCATTGA | 18 |
| DSC-Out-n-021 | TGGTGGTTCGGGGAAAG | 18 |
| DSC-Out-n-022 | AAGACGGAGTGAGAAGT | 18 |
| DSC-Out-n-023 | GTTGGGTAATCACCAGT | 18 |
| DSC-Out-n-024 | GCTCATTTTAATGAAAA | 18 |
| DSC-Out-n-025 | AAATCAAAATGAGTAAC | 18 |
| DSC-Out-n-026 | TAAAGAACGTAAATCGG | 18 |
| DSC-Out-n-027 | CGCTTTCCACGAAAGGA | 18 |
| DSC-Out-n-028 | CATACGAGCCCGATTAA | 18 |
| DSC-Out-n-029 | GCTTCTAATGTCTGTCC | 18 |
| DSC-Out-n-030 | GTGGTTGTGTGACGCTC | 18 |
| DSC-Out-n-031 | CGTGCATCTACAGAGAT | 18 |
| DSC-Out-n-032 | AACCCGTCGTTAACACC | 18 |
| DSC-Out-n-033 | CTTTTGATAAAACCCTC | 18 |
| DSC-Out-n-034 | TCGCGTTTTCGTATTAA | 18 |
| DSC-Out-n-035 | GGCATAGTAGGAGCGGA | 18 |
| DSC-Out-n-036 | GGCCCACTAGAAAAACC | 18 |
| DSC-Out-n-037 | TTTGACGAGCTGGGGTG | 18 |
| DSC-Out-n-038 | CAGAACAATTGTCCTTA | 18 |
| DSC-Out-n-039 | GCCATTAAAGCGGATTG | 18 |
| DSC-Out-n-040 | GTCAATAGACAGACCGG | 18 |
| DSC-Out-n-041 | CATTACCGCGTTTTTAT | 18 |
| DSC-Out-n-042 | GCCGCGCTTCGCTGCGC | 18 |
| DSC-Out-n-043 | TACCGACAAGCCAGTAA | 18 |
| DSC-Out-n-044 | CTGAGTAGATGATTAGT | 18 |
| DSC-Out-n-045 | ATTTCATCTCTTTTTCA | 18 |
| DSC-Out-n-046 | GCTATTAGTGGCACAGA | 18 |
| DSC-Out-n-047 | TGAGTGAATAACAGTAC | 18 |
| DSC-Out-n-048 | TAAAATATCGAAAGGAA | 18 |
| DSC-Out-n-049 | GCGTAGATTCGTAAAAC | 18 |
| DSC-Out-n-050 | AAGATTCATAATTCATC | 18 |
| DSC-Out-n-051 | TAGACGGGAGGTATTAA | 18 |
| DSC-Out-n-052 | TAATAAGTTTTTAACAA | 18 |
| DSC-Out-n-053 | ACAGCCCTCTGCTGATG | 18 |
| DSC-Out-n-054 | ACGAGGGTAATTACATT | 18 |
| DSC-Out-n-055 | TCATTACCCATAATGGA | 18 |
| DSC-Out-n-056 | TATCCGGTAAAATGAAA | 18 |
| DSC-Out-n-057 | ATTTACGAGCAAAGTCA | 18 |
| DSC-Out-n-058 | AAACAACATTTACCGTT | 18 |
| DSC-Out-n-059 | ACAAATTCTGTAACAGT | 18 |
| DSC-Out-n-060 | CGTGTGATAGTAACACT | 18 |
| DSC-Out-n-061 | AGGTCTGAGAGTTTTGT | 18 |
| DSC-Out-n-062 | ATTTTCCCTTTAAAGGC | 18 |
| DSC-Out-n-063 | ATTCATTTCAGGACTAA | 18 |
| DSC-Out-n-064 | AGTACCTTTGGCGCATA | 18 |
| DSC-Out-n-065 | GTTAATAAATCATTCAG | 18 |
| DSC-Out-n-066 | CAATAGATAATAGCTAT | 18 |
| DSC-Out-n-067 | CATAATTACAGAAGGAT | 18 |
| DSC-Out-n-068 | TAAGACGCTGAACAACT | 18 |
| DSC-Out-n-069 | TTGCTTTGAGAATACAC | 18 |
| DSC-Out-n-070 | TGGCTCATTTTACCTTA | 18 |
| DSC-Out-n-071 | AAGATTAGTTATCCCAA | 18 |
| DSC-Out-n-072 | ATAATAAGACCACAAGA | 18 |
| DSC-Out-n-073 | GTTACCAGAAAATCCTC | 18 |
| DSC-Out-n-074 | GAAAGTATTCGGAACCT | 18 |
| DSC-Out-n-075 | GAGAGGGTTCTCATTTT | 18 |
| DSC-Out-n-076 | CAACAGTTTATGGGATT | 18 |
| DSC-Out-n-077 | AAAAGGCTCCACGCATA | 18 |
| DSC-Out-n-078 | AGGCACCAATAAAATAC | 18 |
| DSC-Out-n-079 | AAAGTACAATGACCAAC | 18 |
| DSC-Out-n-080 | TGGTTTAATCGAGTAGT | 18 |
| DSC-Out-n-081 | CGCTAACGAAATTTTAT | 18 |
| DSC-Out-n-082 | GCCAGCATTAATAACGG | 18 |
| DSC-Out-n-083 | GTTTAGTACAGGTGTAT | 18 |
| DSC-Out-n-084 | GGTGAATTTTCGGTTTA | 18 |
| DSC-Out-n-085 | ACCTGCTCCGATAAATT | 18 |
| DSC-Out-n-086 | GTCACCAATCGATTGAG | 18 |
| DSC-Out-n-087 | CGACTTGAGTAAAGGTG | 18 |
| DSC-Out-n-088 | ACCACCCTCTACATACA | 18 |
| DSC-Out-n-089 | CACCGGAACACCACGGA | 18 |
| DSC-Out-n-090 | TGTAGCGCGATATGGTT | 18 |
| DSC-Out-b-001 | ATCAATATGTAGCATTAAAATTATCTACCACAACTCAC | 39 |
| DSC-Out-b-002 | AAGGCTATCGGGCGCGAAAATTATCTACCACAACTCAC | 39 |
| DSC-Out-b-003 | TGTCAATCATTCGCAAAAAATTATCTACCACAACTCAC | 39 |
| DSC-Out-b-004 | CCCTGTAATCGGTTGTAAAATTATCTACCACAACTCAC | 39 |
| DSC-Out-b-005 | TAAATGCAATAGCAAAAAAATTATCTACCACAACTCAC | 39 |
| DSC-Out-b-006 | GATTGCCCTCACCAGTGAAATTATCTACCACAACTCAC | 39 |
| DSC-Out-b-007 | TAAATCATTTGAATCGGAAATTATCTACCACAACTCAC | 39 |
| DSC-Out-b-008 | GCAACTGTTGCGCCATTAAATTATCTACCACAACTCAC | 39 |
| DSC-Out-b-009 | AAAACAGGAGAAGTTTCAAATTATCTACCACAACTCAC | 39 |
| DSC-Out-b-010 | GCGTCCAATTAGTAAAAAAATTATCTACCACAACTCAC | 39 |
| DSC-Out-b-011 | TAGGGTTGATAAATCAAAAATTATCTACCACAACTCAC | 39 |
| DSC-Out-b-012 | AATCGGCCAGTTGCAGCAAATTATCTACCACAACTCAC | 39 |
| DSC-Out-b-013 | GTTTCCTGTAATTCGTAAAATTATCTACCACAACTCAC | 39 |
| DSC-Out-b-014 | ACCGACAGTGTTGGTGTAAATTATCTACCACAACTCAC | 39 |
| DSC-Out-b-015 | GCCAAGCTTGACGTTGTAAATTATCTACCACAACTCAC | 39 |
| DSC-Out-b-016 | CAGGAAGATCCTCTTCGAAATTATCTACCACAACTCAC | 39 |
| DSC-Out-b-017 | CCTGTAGCCAAAAATAAAAATTATCTACCACAACTCAC | 39 |
| DSC-Out-b-018 | TTGCTGAATAAATTGTAAAATTATCTACCACAACTCAC | 39 |
| DSC-Out-b-019 | TGCATCAAATTATAGTCAAATTATCTACCACAACTCAC | 39 |
| DSC-Out-b-020 | CGACGATAATTCATTGAAAATTATCTACCACAACTCAC | 39 |
| DSC-Out-b-021 | TGGTGGTTCGGGGAAAGAAATTATCTACCACAACTCAC | 39 |
| DSC-Out-b-022 | AAGACGGAGTGAGAAGTAAATTATCTACCACAACTCAC | 39 |
| DSC-Out-b-023 | GTTGGGTAATCACCAGTAAATTATCTACCACAACTCAC | 39 |
| DSC-Out-b-024 | GCTCATTTTAATGAAAAAAATTATCTACCACAACTCAC | 39 |
| DSC-Out-b-025 | AAATCAAAATGAGTAACAAATTATCTACCACAACTCAC | 39 |
| DSC-Out-b-026 | TAAAGAACGTAAATCGGAAATTATCTACCACAACTCAC | 39 |
| DSC-Out-b-027 | CGCTTTCCACGAAAGGAAAATTATCTACCACAACTCAC | 39 |
| DSC-Out-b-028 | CATACGAGCCCGATTAAAAATTATCTACCACAACTCAC | 39 |
| DSC-Out-b-029 | GCTTCTAATGTCTGTCCAAATTATCTACCACAACTCAC | 39 |
| DSC-Out-b-030 | GTGGTTGTGTGACGCTCAAATTATCTACCACAACTCAC | 39 |
| DSC-Out-b-031 | CGTGCATCTACAGAGATAAATTATCTACCACAACTCAC | 39 |
| DSC-Out-b-032 | AACCCGTCGTTAACACCAAATTATCTACCACAACTCAC | 39 |
| DSC-Out-b-033 | CTTTTGATAAAACCCTCAAATTATCTACCACAACTCAC | 39 |
| DSC-Out-b-034 | TCGCGTTTTCGTATTAAAAATTATCTACCACAACTCAC | 39 |
| DSC-Out-b-035 | GGCATAGTAGGAGCGGAAAATTATCTACCACAACTCAC | 39 |
| DSC-Out-b-036 | GGCCCACTAGAAAAACCAAATTATCTACCACAACTCAC | 39 |
| DSC-Out-b-037 | TTTGACGAGCTGGGGTGAAATTATCTACCACAACTCAC | 39 |
| DSC-Out-b-038 | CAGAACAATTGTCCTTAAAATTATCTACCACAACTCAC | 39 |
| DSC-Out-b-039 | GCCATTAAAGCGGATTGAAATTATCTACCACAACTCAC | 39 |
| DSC-Out-b-040 | GTCAATAGACAGACCGGAAATTATCTACCACAACTCAC | 39 |
| DSC-Out-b-041 | CATTACCGCGTTTTTATAAATTATCTACCACAACTCAC | 39 |
| DSC-Out-b-042 | GCCGCGCTTCGCTGCGCAAATTATCTACCACAACTCAC | 39 |
| DSC-Out-b-043 | TACCGACAAGCCAGTAAAAATTATCTACCACAACTCAC | 39 |
| DSC-Out-b-044 | CTGAGTAGATGATTAGTAAATTATCTACCACAACTCAC | 39 |
| DSC-Out-b-045 | ATTTCATCTCTTTTTCAAAATTATCTACCACAACTCAC | 39 |
| DSC-Out-b-046 | GCTATTAGTGGCACAGAAAATTATCTACCACAACTCAC | 39 |
| DSC-Out-b-047 | TGAGTGAATAACAGTACAAATTATCTACCACAACTCAC | 39 |
| DSC-Out-b-048 | TAAAATATCGAAAGGAAAAATTATCTACCACAACTCAC | 39 |
| DSC-Out-b-049 | GCGTAGATTCGTAAAACAAATTATCTACCACAACTCAC | 39 |
| DSC-Out-b-050 | AAGATTCATAATTCATCAAATTATCTACCACAACTCAC | 39 |
| DSC-Out-b-051 | TAGACGGGAGGTATTAAAAATTATCTACCACAACTCAC | 39 |
| DSC-Out-b-052 | TAATAAGTTTTTAACAAAAATTATCTACCACAACTCAC | 39 |
| DSC-Out-b-053 | ACAGCCCTCTGCTGATGAAATTATCTACCACAACTCAC | 39 |
| DSC-Out-b-054 | ACGAGGGTAATTACATTAAATTATCTACCACAACTCAC | 39 |
| DSC-Out-b-055 | TCATTACCCATAATGGAAAATTATCTACCACAACTCAC | 39 |
| DSC-Out-b-056 | TATCCGGTAAAATGAAAAAATTATCTACCACAACTCAC | 39 |
| DSC-Out-b-057 | ATTTACGAGCAAAGTCAAAATTATCTACCACAACTCAC | 39 |
| DSC-Out-b-058 | AAACAACATTTACCGTTAAATTATCTACCACAACTCAC | 39 |
| DSC-Out-b-059 | ACAAATTCTGTAACAGTAAATTATCTACCACAACTCAC | 39 |
| DSC-Out-b-060 | CGTGTGATAGTAACACTAAATTATCTACCACAACTCAC | 39 |
| DSC-Out-b-061 | AGGTCTGAGAGTTTTGTAAATTATCTACCACAACTCAC | 39 |
| DSC-Out-b-062 | ATTTTCCCTTTAAAGGCAAATTATCTACCACAACTCAC | 39 |
| DSC-Out-b-063 | ATTCATTTCAGGACTAAAAATTATCTACCACAACTCAC | 39 |
| DSC-Out-b-064 | AGTACCTTTGGCGCATAAAATTATCTACCACAACTCAC | 39 |
| DSC-Out-b-065 | GTTAATAAATCATTCAGAAATTATCTACCACAACTCAC | 39 |
| DSC-Out-b-066 | CAATAGATAATAGCTATAAATTATCTACCACAACTCAC | 39 |
| DSC-Out-b-067 | CATAATTACAGAAGGATAAATTATCTACCACAACTCAC | 39 |
| DSC-Out-b-068 | TAAGACGCTGAACAACTAAATTATCTACCACAACTCAC | 39 |
| DSC-Out-b-069 | TTGCTTTGAGAATACACAAATTATCTACCACAACTCAC | 39 |
| DSC-Out-b-070 | TGGCTCATTTTACCTTAAAATTATCTACCACAACTCAC | 39 |
| DSC-Out-b-071 | AAGATTAGTTATCCCAAAAATTATCTACCACAACTCAC | 39 |
| DSC-Out-b-072 | ATAATAAGACCACAAGAAAATTATCTACCACAACTCAC | 39 |
| DSC-Out-b-073 | GTTACCAGAAAATCCTCAAATTATCTACCACAACTCAC | 39 |
| DSC-Out-b-074 | GAAAGTATTCGGAACCTAAATTATCTACCACAACTCAC | 39 |
| DSC-Out-b-075 | GAGAGGGTTCTCATTTTAAATTATCTACCACAACTCAC | 39 |
| DSC-Out-b-076 | CAACAGTTTATGGGATTAAATTATCTACCACAACTCAC | 39 |
| DSC-Out-b-077 | AAAAGGCTCCACGCATAAAATTATCTACCACAACTCAC | 39 |
| DSC-Out-b-078 | AGGCACCAATAAAATACAAATTATCTACCACAACTCAC | 39 |
| DSC-Out-b-079 | AAAGTACAATGACCAACAAATTATCTACCACAACTCAC | 39 |
| DSC-Out-b-080 | TGGTTTAATCGAGTAGTAAATTATCTACCACAACTCAC | 39 |
| DSC-Out-b-081 | CGCTAACGAAATTTTATAAATTATCTACCACAACTCAC | 39 |
| DSC-Out-b-082 | GCCAGCATTAATAACGGAAATTATCTACCACAACTCAC | 39 |
| DSC-Out-b-083 | GTTTAGTACAGGTGTATAAATTATCTACCACAACTCAC | 39 |
| DSC-Out-b-084 | GGTGAATTTTCGGTTTAAAATTATCTACCACAACTCAC | 39 |
| DSC-Out-b-085 | ACCTGCTCCGATAAATTAAATTATCTACCACAACTCAC | 39 |
| DSC-Out-b-086 | GTCACCAATCGATTGAGAAATTATCTACCACAACTCAC | 39 |
| DSC-Out-b-087 | CGACTTGAGTAAAGGTGAAATTATCTACCACAACTCAC | 39 |
| DSC-Out-b-088 | ACCACCCTCTACATACAAAATTATCTACCACAACTCAC | 39 |
| DSC-Out-b-089 | CACCGGAACACCACGGAAAATTATCTACCACAACTCAC | 39 |
| DSC-Out-b-090 | TGTAGCGCGATATGGTTAAATTATCTACCACAACTCAC | 39 |
| DNA Icosahedron Captor | | |
| DIC-Vertex-001 | AGACATTTTTGTCAAATCACCGTACCCCGGTTTTTTTGATAATCAGAAA | 49 |
| DIC-Vertex-002 | AATTCTTTTTGCGTCTGGCCTAGTATCGGCCTTTTTTCAGGAAGATCGC | 49 |
| DIC-Vertex-003 | TTTTGTTTTTTTAAAATTCGCA | 22 |
| DIC-Vertex-004 | AATTATTTTTACCGTTGTAGCGATAGGGTTGATTTTTGTGTTG | 43 |
| DIC-Vertex-005 | TAGGGTTTTTCGCTGGCAAGTGGAACGGTACGTTTTTCCAGAATCCTGA | 49 |
| DIC-Vertex-006 | TTCCACCCCCGATTTATTTTTGAGCTTGACGGG | 33 |
| DIC-Vertex-007 | ATTGCTTTTTTCCTTTTGATATAGATACATTTTTTTTCGCAAATGGTCA | 49 |
| DIC-Vertex-008 | CCAATTTTTTACTGCGGAATCATCGCGTTTTATTTTTATTCGAGCTTCA | 49 |
| DIC-Vertex-009 | TTCTATTTTTCTAATAGTAGTA | 22 |
| DIC-Vertex-010 | AGGCTTTTTTTTTGCAAAAGACCTCATATATTTTTTTTTAAAT | 43 |
| DIC-Vertex-011 | AAGTTTTTTTTGAGTAACATTAGGAATTACGATTTTTGGCATAGTAAGA | 49 |
| DIC-Vertex-012 | GCAATGCCAGGGTTTTTTTTTCCCAGTCACGAC | 33 |
| DIC-Vertex-013 | TAGACTTTTTTTTACAAACAACGCCCTGGAGTTTTTTGACTCTATGATA | 49 |
| DIC-Vertex-014 | ATAAATTTTTACAGAGGTGAGCTAAAATATCTTTTTTTTAGGAGCACTA | 49 |
| DIC-Vertex-015 | GGCCTTTTTTTGAATCGGCTGA | 22 |
| DIC-Vertex-016 | ATGCGTTTTTCGAACTGATAGTTATCCGCTCATTTTTCAATTC | 43 |
| DIC-Vertex-017 | TCAAATTTTTCTATCGGCCTTCAGAGATAGAATTTTTCCCTTCTGACCT | 49 |
| DIC-Vertex-018 | CACACGCGTATTGGGCTTTTTGCCAGGGTGGTT | 33 |
| DIC-Vertex-019 | TCACCTTTTTGTACTCAGGAGCAGCCCTCATATTTTTGTTAGCGTAACG | 49 |
| DIC-Vertex-020 | GGGTTTTTTTTTGCTCAGTACC | 22 |
| DIC-Vertex-021 | TTTGCTTTTTTAAACAACTTTGATACCGATAGTTTTTTTGCGCCGACAA | 49 |
| DIC-Vertex-022 | CATGAGAAGTTTCCATTTTTTTAAACGGGTAAA | 33 |
| DIC-Vertex-023 | AATAATTTTTATCCTCATTAAAACCTATTATTTTTTTCTGAAA | 43 |
| DIC-Vertex-024 | ACTAATTTTTAACACTCATCTAAAGAGGACAGTTTTTATGAACGGTGTA | 49 |
| DIC-Vertex-025 | CCGCCTTTTTGCCAGCATTGATCAACTTTAATTTTTTCATTGTGAATTA | 49 |
| DIC-Vertex-026 | TTGAGTTTTTCGCTAATATCAG | 22 |
| DIC-Vertex-027 | CATATTTTTTTCCTGATTATCCAGTACCTTTTTTTTTACATCGGGAGAA | 49 |
| DIC-Vertex-028 | GCGCAACAGTACATAATTTTTATCAATATATGT | 33 |
| DIC-Vertex-029 | ATTTTTTTTTGCACCCAGCTAAACATAAAAACTTTTTAGGGAA | 43 |
| DIC-Vertex-030 | TCCTTTTTTTGAAAACATAGCTTTTTCAAATATTTTTTATTTTAGTTAA | 49 |
| DIC-Vertex-031 | GGCGTTTTTTTTTAGCGAACCCTTAATTGAGATTTTTATCGCCATATTT | 49 |
| DIC-Vertex-032 | TAGGATTTTTATCATTACCGCG | 22 |
| DIC-Vertex-033 | TATAATTTTTAGTACCGACAATCCTTATCATTTTTTTCCAAGAACGGGT | 49 |
| DIC-Vertex-034 | AAAAGTTTTTAAACGCAAAGAGCCATTTGGGATTTTTATTAGA | 43 |
| DIC-Vertex-035 | GCCAGTTAGCGTTTGCTTTTTCATCTTTTCATA | 33 |
| DIC-Vertex-036 | CCCTCTTTTTAGAACCGCCACAACTGGCATGATTTTTTTAAGACTCCTT | 49 |
| DIC-Vertex-037 | ACTCACATTAATTGGGCGATGGCCCGTTAATA | 32 |
| DIC-Vertex-038 | AACCGTCTATCATATCGTAAAACT | 24 |
| DIC-Vertex-039 | GATGAACGGTATCTGTTGGGAAGGAGGCCGG | 31 |
| DIC-Vertex-040 | CAGGCTGCGCAATCATCTGCCAGT | 24 |
| DIC-Vertex-041 | TCGTAACCGTGTTGCGTTGCGCTCCAAAAAT | 31 |
| DIC-Vertex-042 | ATCGGCAAAATTTTATCAACAATATCACGCA | 31 |
| DIC-Vertex-043 | GAACGCGCCTGTTGGAGGCCGATT | 24 |
| DIC-Vertex-044 | GGAGCTAAACATAATATGCAACTACGGGCGC | 31 |
| DIC-Vertex-045 | CAACATGTTTTATAGCACTAAATC | 24 |
| DIC-Vertex-046 | AGGTGCCGTAATCCCTTATAAAT | 23 |
| DIC-Vertex-047 | TTCCCAATTCTTTTGAAAATCTCCACCTTTA | 31 |
| DIC-Vertex-048 | AATTTTTTCACGTTAAGAGGAAGC | 24 |
| DIC-Vertex-049 | ATCAAAAAGATTCTAAATCGGTTGATAGCGT | 31 |
| DIC-Vertex-050 | CAGAGCATAAAGTCTACAAAGGCTAGCATCAA | 32 |
| DIC-Vertex-051 | TTTTTGAGAGATTGCGAACGAGTA | 24 |
| DIC-Vertex-052 | AGCCTTTATTTTTTGTGTCGAAATTAGCGAG | 31 |
| DIC-Vertex-053 | TCGCCTGATAAATCAACTAATGCA | 24 |
| DIC-Vertex-054 | AATACCACATTTATGCGCACGACTATTTTAA | 31 |
| DIC-Vertex-055 | GGTTGTGAATTCTTGCTGCAAGGC | 24 |
| DIC-Vertex-056 | AAAGGGGGATGTCAACGCAAGGA | 23 |
| DIC-Vertex-057 | TCCCGCCAAAATAACCTACCATATGAAGTAT | 31 |
| DIC-Vertex-058 | TGGAAGGGTTAGTCAGTTGAAAGG | 24 |
| DIC-Vertex-059 | TGGCAAATCAATGGTGCTTGTTACGCAGAAG | 31 |
| DIC-Vertex-060 | CTTAAGCTACGTTAAACGGCGGATTATATAGG | 32 |
| DIC-Vertex-061 | CTCCGTGGGAACTTAACCCCGCTT | 24 |
| DIC-Vertex-062 | TCGTAATCATGTACCTTTTTAACCGTCTTTA | 31 |
| DIC-Vertex-063 | GTCTGAGAGACTTCAGTAATAAAA | 24 |
| DIC-Vertex-064 | AGTCACACGACTCCACGCTGGTTTGAAGAAC | 31 |
| DIC-Vertex-065 | GCAGCAAGCGGTTCGGCCAACGCG | 24 |
| DIC-Vertex-066 | CATTAATGAATTGTCATAGCTGT | 23 |
| DIC-Vertex-067 | CACCAGTACAATGCACCGTAATCAAGGTGTA | 31 |
| DIC-Vertex-068 | CCATCGATAGCATGGGATCGTCACCATTAGCG | 32 |
| DIC-Vertex-069 | GGCCGCTTTTGCTTTTCGAGGTGA | 24 |
| DIC-Vertex-070 | TATCAGCTTGCTTGGTTGCTTTGAATGGGAT | 31 |
| DIC-Vertex-071 | GGGCGCGTACTATACTACAACGCC | 24 |
| DIC-Vertex-072 | GCTACAGAGGCTACAGTTAATGC | 23 |
| DIC-Vertex-073 | TGCCCGTATAATACGTAACAAAGCACAAACA | 31 |
| DIC-Vertex-074 | TTACCCAAATCATATAAGGGAACC | 24 |
| DIC-Vertex-075 | GACGGTCAATCTCAAAAATCAGGTAGAATAC | 31 |
| DIC-Vertex-076 | ATGACCATAAATTTTTGAGGACTA | 24 |
| DIC-Vertex-077 | CAGAACGAGTATGAAGCCCTTTTTACCAGAG | 31 |
| DIC-Vertex-078 | AGCTATCTTACCTCCTGAGCAAAAGAGGGTAA | 32 |
| DIC-Vertex-079 | TCATTTCAATTATGTTTAACGTCA | 24 |
| DIC-Vertex-080 | TAGATTTTCAGTCTAACGGAACAATTATCAT | 31 |
| DIC-Vertex-081 | TAATAAAACGAATGTAAATTGGGC | 24 |
| DIC-Vertex-082 | AAAAATCTAAAGTTCATTTGAATT | 24 |
| DIC-Vertex-083 | ATTTAACAATTTGAAAATAGCAG | 23 |
| DIC-Vertex-084 | ACGTCAAAAATTCCGGAATCATAAAGTTGCT | 31 |
| DIC-Vertex-085 | TAAGAATAAACATATCGCAAGACA | 24 |
| DIC-Vertex-086 | ATGCAAATCCATCATCACCTTGCTCTTAGAA | 31 |
| DIC-Vertex-087 | AATTCTTACCATAAAGGGCGACATAACGCGA | 31 |
| DIC-Vertex-088 | GCGCCAAAGACATTCAGGGATAGCATTCATCG | 32 |
| DIC-Vertex-089 | CCACCCTCATTTTAGAAACCAATC | 24 |
| DIC-Vertex-090 | TACGAGCATGTTCATTTTGACGCTAAGAGAA | 31 |
| DIC-Vertex-091 | TGGAAATACCTATGTATAAAGCCA | 24 |
| DIC-Vertex-092 | GCGCGTTTTCATGGTGAATTATC | 23 |
| DIC-Vertex-093 | GCTTTTGATGATTTCGGCATTTTC | 24 |
| DIC-Vertex-094 | AAACCGAGGAATACAGGAGTGTACGCCGCCA | 31 |
| DIC-Vertex-095 | CCAGTTACAAAATACGCAATAATA | 24 |
| DIC-Vertex-096 | TATTCATTAAATTAAACAGCCATAAACATAT | 31 |
| DIC-Red-001 | CCCAAATCGGACTCCAAGATTGTACACTATTAAAAGGGAGGTTTGGAATGGGGTCG | 56 |
| DIC-Red-002 | GAGTCTGGAATTAATGTATTTTCAACCGTTCTAATCATATATCAATATAGAGAATC | 56 |
| DIC-Red-003 | TCTTCGCTCCAGGCAACGGCACCGCTTCTGGTTGGGTAACGCCTGAGTAGCTGGCG | 56 |
| DIC-Red-004 | GTCACGTTGAGCGAGTGCCATCTGCATCAACAACGACGACTCCTGTAGTGGGCGCA | 56 |
| DIC-Red-005 | TCGGGAAACTGGGGTGTCAGCTCAGCATAAAGGGCGGTTTAACATACGGCCAGCTG | 56 |
| DIC-Red-006 | AGTTTCATATTGCTGAGGCGAGAAGATGGCTTTTGACCATAGAGGTCAACAGTTGA | 56 |
| DIC-Red-007 | ACCCTGTATAGCAAAAAATCATACAGGCAAGGTTTAGAACAGTTTTGCGCGGGAGA | 56 |
| DIC-Red-008 | TGAATTGTGCCAGGGTCAGTGCCAAGCTTTCTTTTACGCTTTCGACAATGACAATG | 56 |
| DIC-Red-009 | GATCCCCGGGTTGGTGCATTTCTCCGAACTCTTGAAATTGCCCTAAAAGCTCGAAT | 56 |
| DIC-Red-010 | ATCCTGTTTCACCGCCAGACGGGCAACAGCTGTAGCCCGAAATACTTCGTTCCGAA | 56 |
| DIC-Red-011 | GAAAAATATAAACAACCAGTGAGGGTCCAGACGCTGTCTTAAGGTAAATCCTAATT | 56 |
| DIC-Red-012 | GCTTTCCTCGCCGCGCTCTTTCCAGCGTAACCTTTAGACAGTAGCGGTTCAGAGCG | 56 |
| DIC-Red-013 | AAGGAGCCACAACTAAAAGCAAACGTGAGAATAACAGCTTCAACAGTTTATCGGTT | 56 |
| DIC-Red-014 | ATAGTCAGCTTTAAACCGAAGGCAGAATCCCCCTTCAAATGTCATAAACGGATTGC | 56 |
| DIC-Red-015 | GTTACTTAAAAGTACACCTCGTTTATACCAAGCAACTTTGTTGACCCCGAGGCGCA | 56 |
| DIC-Red-016 | AAGATTCAGACGTTGGGAACTGGCTCATTATAACGCCAAAATCATTTTGATTTAGG | 56 |
| DIC-Red-017 | TAAAACAGTGTTTGGAAGCCGTCATCAATATATACAGTAAAGATGATGAAATTGCG | 56 |
| DIC-Red-018 | ACCCTCAACACGCTGATTCTGTAACCGCCTGCAAGGTTATGCGGTCAGTGGTCAGT | 56 |
| DIC-Red-019 | TTATATAAATAGTGAAGTGGCACAACGCTGAGGAGAAAACGATAGCTTAAATGCTG | 56 |
| DIC-Red-020 | ATTATTTACCATTGCAAATTTAGGACAATATTCTGGCCAAGCTGGTAAGATTCACC | 56 |
| DIC-Red-021 | ATGTACCGTCAGAACCCACTCATCCTCAGAACTCCACAGAGTTTAGTAAGTTTCGT | 56 |
| DIC-Red-022 | AGCATCGGCGCTGAGGCCACGCATAACCGATATTCATGAGAAGTATTATAGCAACG | 56 |
| DIC-Red-023 | AAGGCTTGATCTTGACGCTGGCTGACCTTCATGTTTAATTCAGGAGGTAGAAACAC | 56 |
| DIC-Red-024 | CATCAAGACAAAATCGCTGATTGCTTTGAATATAATGGAATTAGACGGTTAATTAC | 56 |
| DIC-Red-025 | GTTTAGTACCGTGTGAAAATTTAATGGTTTGACAGTAGGGTCCCGACTGTTATACA | 56 |
| DIC-Red-026 | GTTTGCCTAGGCCGGATCGAGAGGACCATTACCCCCCTTACAAAATCAAGACTGTA | 56 |
| DIC-Red-027 | CGGGGTCACCGTTCCACCAGAGCCGCAGTCTCTATTTCGGAGCCAGAAAGTAACAG | 56 |
| DIC-Red-028 | TAGCCGAAAGCAAGAAAATTGAGTTAAGCCCACCCAAAAGCCTCAGAGCCAGAAGG | 56 |
| DIC-Red-029 | AAATAAGAAGCGTCTTGCAAACGTCTTACCAAGAGAGAATCAATTTTATTTGTTTA | 56 |
| DIC-Red-030 | GGGAAGGTATAGAAAATCAGATATTATTTTGTCGACTTGACACCACGGACGGAAAT | 56 |
| DIC-Ora-out-01 | GGAACCCTAAGAACGTAAGTTTTTCAAGAGTCTAAGCAAA | 41 |
| DIC-Ora-out-02 | AGCATGTCAGCTGATAAGCAAACAGATATTCATTTGGGGC | 41 |
| DIC-Ora-out-03 | GATTAAGTGCCGGAAAATTACGCCAATGTGTAGGTAAAGA | 41 |
| DIC-Ora-out-04 | TTGAGGGGTTAAATGTGGTGTAGACCAGCTTTTAAGCAAC | 41 |
| DIC-Ora-out-05 | CGGGGAGATGTAAAGCCCTGTCGTAGCCGGAATTTTTTAA | 41 |
| DIC-Ora-out-06 | GATTTAGTAGAGCTTATCCATATATTTTTGCGAGGAAGGG | 41 |
| DIC-Ora-out-07 | TAAAAATTCAAAGAATATACTTTTCAGAGGGGGTAATAGT | 41 |
| DIC-Ora-out-08 | CTAATCTACAGGAGAACAACCTTACTCGTATTAAATCCTT | 41 |
| DIC-Ora-out-09 | TTCCTGTGGACCTCCTGGTACCGACATCGCCATTAAAAAT | 41 |
| DIC-Ora-out-10 | CAAAAGAAATTGCCCTTGATGGTGTTTGATTAGTAATAAC | 41 |
| DIC-Ora-out-11 | AATAATCGGACGACAAATATCCCAGTAATTCTCCACCGAG | 41 |
| DIC-Ora-out-12 | AAAGGGATACCACACCCGTTAGAACACGCTGCGACGTTAG | 41 |
| DIC-Ora-out-13 | ATTTCTTAAGAAAGGATTTAATTGTCAGCGGATCCAACAG | 41 |
| DIC-Ora-out-14 | CCGAAAGACTCAAATGAAGCAAAGTATTCATTCCAACCTA | 41 |
| DIC-Ora-out-15 | GAACTGACCGCGAAACGCCGGAACCAGCGATTACCAGACG | 41 |
| DIC-Ora-out-16 | GATACATACCAGTCAGTCAGTTGAGCGGAACAAAGAAACC | 41 |
| DIC-Ora-out-17 | GATGAATAATCCTGATAAATAAAGGCAATTCAATAGATAA | 41 |
| DIC-Ora-out-18 | AATTGAGGAACAGTGCTCAATATCTATTAACAATCGTCGC | 41 |
| DIC-Ora-out-19 | AAGAACGCAAGAGTCACTATATGTAGATTAAGGACAATAT | 41 |
| DIC-Ora-out-20 | GGGACATTACCGCCAGCATTGGCATATCCAGACAGAGGCA | 41 |
| DIC-Ora-out-21 | TGTAGCATCGCCACCCTAACACTGCCGCCACCGAGAACAA | 41 |
| DIC-Ora-out-22 | AAGACTTTTATTCGGTAACGAGGGAGAGGCTGAGACTCCT | 41 |
| DIC-Ora-out-23 | TTGAGATGCAAGAGTACCCTGACGTGAGGCAGGTCAGACG | 41 |
| DIC-Ora-out-24 | ACCTTTTTCCAAGTTAAAACAAAAGAGAATTAACTGAACA | 41 |
| DIC-Ora-out-25 | ACGCTCAAAATACCGATCATATGCTGCGGGAGGTTTTGAA | 41 |
| DIC-Ora-out-26 | GGTCATAGCATTAGCATTAGCGTCCCAGTAGCGTTGATAT | 41 |
| DIC-Ora-out-27 | CCCCTGCCTGAATTTAGTGCCTTGTGGAAAGCACCACCGG | 41 |
| DIC-Ora-out-28 | ACGGAATAATAATAAGCAAAGTTACCACCACCCTCAGAGC | 41 |
| DIC-Ora-out-29 | CCTTTACACGCTAACGAACGATTTTCCTGAATAGAAAATA | 41 |
| DIC-Ora-out-30 | ACCGTCACCACAATCAAAATATTGAATAAGTTAGAAGGCT | 41 |
| DIC-Blue-in-001 | TATTTAAAAAACAGGAACGTCAAAGGGCGAAA | 32 |
| DIC-Blue-in-002 | GCGAGCTGTTTAGCTACCGGAGAGGGTAGCTA | 32 |
| DIC-Blue-in-003 | TTCAAAAGCAGCTTTCAGCGCCATTCGCCATT | 32 |
| DIC-Blue-in-004 | TCGTCGGTGCGGCCCTAACAACCCGTCGGATT | 32 |
| DIC-Blue-in-005 | CCAATAGGTTGTTAAACCTAATGAGTGAGCTA | 32 |
| DIC-Blue-in-006 | AAGAAAGCGCGAACGTATATAATGCTGTAGCT | 32 |
| DIC-Blue-in-007 | AAAATGTTATCCAATATTAAGCAATAAAGCCT | 32 |
| DIC-Blue-in-008 | TGCCCGAAACGACGGCGGATGTTCTTCTAAGT | 32 |
| DIC-Blue-in-009 | ACCGAACGACATAAATTAATGAGTAAACAGGG | 32 |
| DIC-Blue-in-010 | ATCACTTGCACCAGTGTGGCCCTGAGAGAGTT | 32 |
| DIC-Blue-in-011 | TAAAAGAGTTTATAATATGTTCAGCTAATGCA | 32 |
| DIC-Blue-in-012 | TAAATGAATTTTGTCGTTAATGCGCCGCTACA | 32 |
| DIC-Blue-in-013 | GTCAGGATCAGACCGGAGGAATTGCGAATAAT | 32 |
| DIC-Blue-in-014 | AAACGAAATGCCACTAAGTTCAGAAAACGAGA | 32 |
| DIC-Blue-in-015 | ACGATAAAATCATAACACGGAGATTTGTATCA | 32 |
| DIC-Blue-in-016 | ACCAGAAGGATTTTAAGAAGAAAAATCTACGT | 32 |
| DIC-Blue-in-017 | TACATTTGTAGATTAGTTATACTTCTGAATAA | 32 |
| DIC-Blue-in-018 | TATTAATTAACCTTGCGAGCCAGCAGCAAATG | 32 |
| DIC-Blue-in-019 | TTTTGAATAAGAATACTTTATCAAAATCATAG | 32 |
| DIC-Blue-in-020 | TTTTCGAGCAACATGTACAGGAAAAACGCTCA | 32 |
| DIC-Blue-in-021 | GCAAGCCGAAGTACCGGCCACCCTCAGAGCCA | 32 |
| DIC-Blue-in-022 | CAAGAGAAACCATCGCCTTGCAGGGAGTTAAA | 32 |
| DIC-Blue-in-023 | ATTGGCCTGCGCATAGAAGAACCGGATATTCA | 32 |
| DIC-Blue-in-024 | CCCTGAACGGATTCGCCGCAGAGGCGAATTAT | 32 |
| DIC-Blue-in-025 | GCCTTAAATCTGACCTTAAATAAGGCGTTAAA | 32 |
| DIC-Blue-in-026 | AAGTATAGAAGTGCCGAACGTCACCAATGAAA | 32 |
| DIC-Blue-in-027 | AACCGCCTCACCGGAAGTAAGCGTCATACATG | 32 |
| DIC-Blue-in-028 | CGCCACCACCCACAAGACAATGAAATAGCAAT | 32 |
| DIC-Blue-in-029 | CATACATAGTATGTTATCCAGAGCCTAATTTG | 32 |
| DIC-Blue-in-030 | TATCCGGTCAAGCAAATTCATATGGTTTACCA | 32 |
| DIC-Green-in-01 | AGCCCCAATTGTAAACACTACGTGAACCATCACTTCACACCACACTCCATCTA | 53 |
| DIC-Green-in-02 | ATAACCTGAAAAGGTGTCAGGTCATTGCCTGACTTCACACCACACTCCATCTA | 53 |
| DIC-Green-in-03 | ACTCCAGCGGTGAGAAGCGATCGGTGCGGGCCCTTCACACCACACTCCATCTA | 53 |
| DIC-Green-in-04 | CCGACAGTGGGCACGAGACCGTAATGGGATAGCTTCACACCACACTCCATCTA | 53 |
| DIC-Green-in-05 | TTAAATTTAACGCCATACTGCCCGCTTTCCAGCTTCACACCACACTCCATCTA | 53 |
| DIC-Green-in-06 | GAAAGCCGGAAAGGAGAAGTACGGTGTCTGGACTTCACACCACACTCCATCTA | 53 |
| DIC-Green-in-07 | GCATTAACTAGACTGGTACCAAAAACATTATGCTTCACACCACACTCCATCTA | 53 |
| DIC-Green-in-08 | GTTGTAAACGTTATTATAAGTGTCCTTAGTGCCTTCACACCACACTCCATCTA | 53 |
| DIC-Green-in-09 | CGCATTTCAACCACCACTCGATAAAGACGGAGCTTCACACCACACTCCATCTA | 53 |
| DIC-Green-in-10 | TTTCTTTTCCTGAGTAGCCCCAGCAGGCGAAACTTCACACCACACTCCATCTA | 53 |
| DIC-Green-in-11 | GAAGTGTTTCTGTCCAGATAAGTCCTGAACAACTTCACACCACACTCCATCTA | 53 |
| DIC-Green-in-12 | ATCTAAAGTTTTCTGTCGAGCACGTATAACGTCTTCACACCACACTCCATCTA | 53 |
| DIC-Green-in-13 | AAGCGAACTAGAGAGTAAAAAAAAGGCTCCAACTTCACACCACACTCCATCTA | 53 |
| DIC-Green-in-14 | ATACGTAAGAGGCAAACTTTACCCTGACTATTCTTCACACCACACTCCATCTA | 53 |
| DIC-Green-in-15 | GCAACACTAACCAAAACCGCGACCTGCTCCATCTTCACACCACACTCCATCTA | 53 |
| DIC-Green-in-16 | CCTTATGCGAGCGGAACATTATTACAGGTAGACTTCACACCACACTCCATCTA | 53 |
| DIC-Green-in-17 | ACAACTAAAGGATTTACAAAATTATTTGCACGCTTCACACCACACTCCATCTA | 53 |
| DIC-Green-in-18 | GAGTGAATAATTTTCCGAACCTCAAATATCAACTTCACACCACACTCCATCTA | 53 |
| DIC-Green-in-19 | GAAAGCGTGGCTATTATCCGGCTTAGGTTGGGCTTCACACCACACTCCATCTA | 53 |
| DIC-Green-in-20 | AACAACGCCCAGTAATCAATCGTCTGAAATGGCTTCACACCACACTCCATCTA | 53 |
| DIC-Green-in-21 | ATTAAACCTTTTTATTAGCCCAATAGGAACCCCTTCACACCACACTCCATCTA | 53 |
| DIC-Green-in-22 | TGACAACAGGATTAGGCTCAGCAGCGAAAGACCTTCACACCACACTCCATCTA | 53 |
| DIC-Green-in-23 | CAGACCAGTGATATTCTGCTCATTCAGTGAATCTTCACACCACACTCCATCTA | 53 |
| DIC-Green-in-24 | ACAATAACAAAGTCAGAAGATGATGAAACAAACTTCACACCACACTCCATCTA | 53 |
| DIC-Green-in-25 | TTTCATCTTCAAGATTTTACTAGAAAAAGCCTCTTCACACCACACTCCATCTA | 53 |
| DIC-Green-in-26 | AGGCGGATCCCGGAATGTAGCGACAGAATCAACTTCACACCACACTCCATCTA | 53 |
| DIC-Green-in-27 | ATCAAAATCCCTCAGATGGTAATAAGTTTTAACTTCACACCACACTCCATCTA | 53 |
| DIC-Green-in-28 | AGAGATAAGAACCACCAAGAAAAGTAAGCAGACTTCACACCACACTCCATCTA | 53 |
| DIC-Green-in-29 | ATTACGCAAAGGTGGCTTATTTATCCCAATCCCTTCACACCACACTCCATCTA | 53 |
| DIC-Green-in-30 | CCCAATAGATTCTAAGTCAACCGATTGAGGGACTTCACACCACACTCCATCTA | 53 |

**Table S3. Sequence information of DFC handles and TGFβ1 aptamer.**

| Name | Sequence ( 5’ – 3’ ) | Length |
| --- | --- | --- |
| Inner  Handle a | CTTCACACCACACTCCATCTA | 21 |
| Cy5-a’ | Cy5-TAGATGGAGTGTGGTGTGAAG | 21 |
| a’-Apt  (optional 5’-Cy5) | T*CG*CT*CGG*CTT*C*A*CG*AG*ATT*C  GTGT*CGTTGTGT*C*CTGT*A*C*C*CG*C*C  TTG*A*C*C*AGT*C*A*CT*CT*AG*AG*C*A  T*C*CGG*A*CTG-TAGATGGAGTGTGGTGTGAAG | 96 |
| Outer  Handle b | AAATTATCTACCACAACTCAC | 21 |
| b’-Apt | T*CG*CT*CGG*CTT*C*A*CG*AG*ATT*C  GTGT*CGTTGTGT*C*CTGT*A*C*C*CG*C*C  TTG*A*C*C*AGT*C*A*CT*CT*AG*AG*C*A  T*C*CGG*A*CTG- GTGAGTTGTGGTAGATAATTT | 96 |

*** Note：**Phosphorothioates modification are introduced to the A and C bases of the aptamer sequence.

**Table S4. Dosage and administration of antagonistic TGFβ1 aptamers treatment regimens in the literature and our study.**

|  | **Dosage** | **Method of administration** |
| --- | --- | --- |
| ^[1]^TGFβ1 RNA aptamer  (in literature) | 10mg/kg  250μg/mouse (25g body weight)  108μM×200μL / mouse | Subcutaneous in situ injections  Once a day |
| TGFβ1 DNA aptamer (Free or assembled in DFCs) | **Aptamer:** 2mg/kg  50μg/mouse (25g body weight)  3μM×100μL / mouse  =DBC: 20 nM×100μL  =DSC or DSOC: 33.3 nM×100μL | Aptamer:  Intravenous injection  Every other day |
| Combined with aPD-L1  (in our study) | **aPD-L1:** 1mg/kg  25μg×100μL / mouse | aPD-L1:  Intraperitoneal injection at day 11 and 15 post-tumor inoculation |

1. Takahashi, M., Hashimoto, Y. & Nakamura, Y. Anti-TGF-β1 aptamer enhances therapeutic effect of tyrosine kinase inhibitor, gefitinib, on non-small cell lung cancer in xenograft model. Mol Ther Nucleic Acids 29, 969-978, doi:10.1016/j.omtn. 2022.06.001 (2022).

**Table S5**. **Summary of specific *p*-values in Figure 3, 4, 5, S13 and S16.**

| ***p-values in Figure 3*** | | | | | | | | | |  |
| --- | --- | --- | --- | --- | --- | --- | --- | --- | --- | --- |
| **Fig. 3a WB-pSMAD2/3** | | | | | **Fig. 3a WB-pSMAD2/3** | | | | |  |
| NTC *vs.*  (+)TGFβ1 | <0.0001 | | | **** | (+)TGFβ1 *vs.*  (+) DBC | <0.0001 | | | **** |  |
| NTC *vs.*  (+) DBC | <0.0001 | | | **** | (+)TGFβ1 *vs.*  (+) DSC | <0.0001 | | | **** |  |
| NTC *vs.*  (+) DSC | 0.0002 | | | *** | (+)TGFβ1 *vs.*  (+) DIC | <0.0001 | | | **** |  |
| NTC *vs.* (+) DIC | 0.3081 | | | n.s. | (+)TGFβ1 *vs.*  (+) F_Apt_ | <0.0001 | | | **** |  |
| NTC *vs.* (+) F_Apt_ | 0.5878 | | | n.s. |  |  | | |  |  |
| **Fig. 3b WB-FOXP3** | | | | | **Fig. 3b WB-FOXP3** | | | | |  |
| NTC *vs.*  (+)TGFβ1 | 0.0034 | | | ** | (+)TGFβ1 *vs.*  (+) DBC | 0.0043 | | | ** |  |
| NTC *vs.*  (+) DBC | 0.9998 | | | n.s. | (+)TGFβ1 *vs.*  (+) DSC | 0.1402 | | | n.s. |  |
| NTC *vs.*  (+) DSC | 0.1843 | | | n.s. | (+)TGFβ1 *vs.*  (+) DIC | 0.4319 | | | n.s. |  |
| NTC *vs.* (+) DIC | 0.0518 | | | n.s. | (+)TGFβ1 *vs.*  (+) F_Apt_ | 0.8251 | | | n.s. |  |
| NTC *vs.* (+) F_Apt_ | 0.0169 | | | * |  |  | | |  |  |
| **Fig. 3c CD4+CD25+FOXP3+ Treg** | | | | | **Fig. 3c CD4+CD25+FOXP3+ Treg** | | | | |  |
| NTC *vs.*  (+)TGFβ1 | 0.0006 | | | *** | (+)TGFβ1 *vs.*  (+) DBC | 0.0031 | | | ** |  |
| NTC *vs.*  (+) DBC | 0.7746 | | | n.s. | (+)TGFβ1 *vs.*  (+) DSC | 0.0301 | | | * |  |
| NTC *vs.*  (+) DSC | 0.1417 | | | n.s. | (+)TGFβ1 *vs.*  (+) DIC | 0.0886 | | | n.s. |  |
| NTC *vs.* (+) DIC | 0.0493 | | | * | (+)TGFβ1 *vs.*  (+) F_Apt_ | 0.9998 | | | n.s. |  |
| NTC *vs.* (+) F_Apt_ | 0.0008 | | | *** |  |  | | |  |  |
| **Fig. 3e Caspase3/7 MFI** | | | | | **Fig. 3e Caspase3/7 MFI** | | | | |  |
| NTC *vs.*  (+)TGFβ1 | 0.0002 | | | *** | (+)TGFβ1 *vs.*  (+) DBC | 0.0011 | | | ** |  |
| NTC *vs.*  (+) DBC | 0.1722 | | | n.s. | (+)TGFβ1 *vs.*  (+) DSC | 0.9916 | | | n.s. |  |
| NTC *vs.*  (+) DSC | 0.0316 | | | * | (+)TGFβ1 *vs.*  (+) DIC | 0.7680 | | | n.s. |  |
| NTC *vs.* (+) DIC | 0.1021 | | | n.s. | (+)TGFβ1 *vs.*  (+) F_Apt_ | 0.9983 | | | n.s. |  |
| NTC *vs.* (+) F_Apt_ | 0.0071 | | | ** | (+) DBC *vs.*  (+) F_Apt_ | 0.0028 | | | ** |  |
| **Fig. 3f Colocalization area** | | | | | **Fig. 3f Colocalization area** | | | | |  |
| NTC *vs.*  (+)TGFβ1 | 0.0025 | | | ** | (+)TGFβ1 *vs.*  (+) DBC | <0.0001 | | | **** |  |
| NTC *vs.*  (+) DBC | 0.2476 | | | n.s. | (+)TGFβ1 *vs.*  (+) DSC | 0.9985 | | | n.s. |  |
| NTC *vs.*  (+) DSC | 0.0013 | | | ** | (+)TGFβ1 *vs.*  (+) DIC | 0.9997 | | | n.s. |  |
| NTC *vs.* (+) DIC | 0.0005 | | | *** | (+)TGFβ1 *vs.*  (+) F_Apt_ | 0.9698 | | | n.s. |  |
| NTC *vs.* (+) F_Apt_ | 0.0024 | | | ** | (+) DBC *vs.*  (+) F_Apt_ | <0.0001 | | | **** |  |
| **Fig. 3g PBMC 3:1**  **dead tumor cells %** | | | | | **Fig. 3g PBMC 9:1**  **dead tumor cells %** | | | | |  |
| NTC *vs.*  (+)TGFβ1 | 0.0393 | | | * | NTC *vs.*  (+)TGFβ1 | 0.0087 | | | ** |  |
| NTC *vs.*  (+) DBC | 0.077 | | | n.s. | NTC *vs.*  (+) DBC | 0.009 | | | ** |  |
| NTC *vs.*  (+) DSC | 0.2961 | | | n.s. | NTC *vs.*  (+) DSC | 0.0161 | | | * |  |
| NTC *vs.* (+) DIC | 0.9973 | | | n.s. | NTC *vs.* (+) DIC | 0.0298 | | | * |  |
| NTC *vs.* (+) F_Apt_ | 0.3344 | | | n.s. | NTC *vs.* (+) F_Apt_ | 0.9682 | | | n.s. |  |
| **Fig. 3h TCR-T 1:1**  **dead tumor cells %** | | | | | **Fig. 3h TCR-T 3:1**  **dead tumor cells %** | | | | |  |
| NTC *vs.*  (+)TGFβ1 | 0.0067 | | | ** | NTC *vs.*  (+)TGFβ1 | 0.047 | | | * |  |
| NTC *vs.*  (+) DBC | 0.0011 | | | ** | NTC *vs.*  (+) DBC | 0.0144 | | | * |  |
| NTC *vs.*  (+) DSC | 0.0864 | | | n.s. | NTC *vs.*  (+) DSC | 0.0900 | | | n.s. |  |
| NTC *vs.*  (+) DIC | 0.5979 | | | n.s. | NTC *vs.*  (+) DIC | 0.6213 | | | n.s. |  |
| NTC *vs.*  (+) F_Apt_ | 0.2027 | | | n.s. | NTC *vs.*  (+) F_Apt_ | 0.9997 | | | n.s. |  |
| ***p*-values in Figure 4** | | | | | | | | | |  |
| **Fig. 4b Tumor volume ( day 18 )** | | | | | **Fig. 4e Tumor weight** | | | | |  |
| PBS *vs.* F_Apt_ | 0.9946 | | | n.s. | PBS *vs.* F_Apt_ | 0.3618 | | | n.s. |  |
| PBS *vs.* DSC | 0.8587 | | | n.s. | PBS *vs.* DSC | 0.6109 | | | n.s. |  |
| PBS *vs.* DSCO | 0.2674 | | | n.s. | PBS *vs.* DSCO | 0.1303 | | | n.s. |  |
| PBS *vs.* aPD-L1 | 0.0056 | | | ** | PBS *vs.* aPD-L1 | 0.0044 | | | ** |  |
| PBS *vs.* DBC | 0.0033 | | | ** | PBS *vs.* DBC | 0.0038 | | | ** |  |
| PBS *vs.* DBC+aPD-L1 | 0.0001 | | | **** | PBS *vs.* DBC+aPD-L1 | 0.0033 | | | ** |  |
| **Fig. 4g CD45+T cell in tumor** | | | | | **Fig. 4h CD3+T cell in tumor** | | | | |  |
| PBS *vs.* F_Apt_ | 0.7286 | | | n.s. | PBS *vs.* F_Apt_ | 0.4363 | | | n.s. |  |
| PBS *vs.* DSC | 0.5875 | | | n.s. | PBS *vs.* DSC | 0.0251 | | | * |  |
| PBS *vs.* DSCO | 0.1882 | | | n.s. | PBS *vs.* DSCO | 0.0362 | | | * |  |
| PBS *vs.* aPD-L1 | 0.0364 | | | * | PBS *vs.* aPD-L1 | 0.0019 | | | ** |  |
| PBS *vs.* DBC | 0.0137 | | | * | PBS *vs.* DBC | 0.0009 | | | *** |  |
| PBS *vs.* DBC+aPD-L1 | 0.0004 | | | *** | PBS *vs.* DBC+aPD-L1 | <0.0001 | | | **** |  |
| **Fig. 4i CD8+T cell in tumor** | | | | | **Fig. 4j CD4+CD25+FOXP3+T cell in tumor** | | | | |  |
| PBS *vs.* F_Apt_ | 0.9787 | | | n.s. | PBS *vs.* F_Apt_ | 0.9823 | | | n.s. |  |
| PBS *vs.* DSC | 0.2422 | | | n.s. | PBS *vs.* DSC | 0.9905 | | | n.s. |  |
| PBS *vs.* DSCO | 0.2869 | | | n.s. | PBS *vs.* DSCO | 0.4397 | | | n.s. |  |
| PBS *vs.* aPD-L1 | 0.0226 | | | * | PBS *vs.* aPD-L1 | 0.3828 | | | n.s. |  |
| PBS *vs.* DBC | 0.0118 | | | * | PBS *vs.* DBC | 0.1488 | | | n.s. |  |
| PBS *vs.* DBC+aPD-L1 | 0.0009 | | | *** | PBS *vs.* DBC+aPD-L1 | 0.2276 | | | n.s. |  |
| **Fig. 4k CD11b+Gr1+MDSC  in tumor** | | | | | **Fig. 4k CD11b+Gr1+MDSC  in tumor** | | | | |  |
| PBS *vs.* F_Apt_ | 0.9998 | | | n.s. | aPD-L1 *vs.* PBS | 0.7637 | | | n.s. |  |
| PBS *vs.* DSC | 0.9996 | | | n.s. | aPD-L1 *vs.* F_Apt_ | 0.8536 | | | n.s. |  |
| PBS *vs.* DSCO | 0.0362 | | | * | aPD-L1 *vs.* DSC | 0.9186 | | | n.s. |  |
| PBS *vs.* aPD-L1 | 0.7637 | | | n.s. | aPD-L1 *vs.* DSCO | 0.2452 | | | n.s. |  |
| PBS *vs.* DBC | 0.0155 | | | * | aPD-L1 *vs.* DBC | 0.1165 | | | n.s. |  |
| PBS *vs.* DBC+aPD-L1 | 0.0005 | | | *** | aPD-L1 *vs.* DBC+aPD-L1 | 0.0033 | | | ** |  |
| **Fig. 4l CD11b+CD11c+DC in spleen** | | | | | | | | | | |
| PBS *vs.* F_Apt_ | 0.9753 | | | n.s. | PBS *vs.* aPD-L1 | | 0.9392 | | n.s. | |
| PBS *vs.* DSC | 0.9072 | | | n.s. | PBS *vs.* DBC | | 0.0053 | | ** | |
| PBS *vs.* DSCO | 0.2958 | | | n.s. | PBS *vs.* DBC+aPD-L1 | | 0.0554 | | n.s. | |
| **Fig. 4m CD11b+Gr1+MDSC in spleen** | | | | | **Fig. 4m CD11b+Gr1+MDSC in spleen** | | | | | |
| PBS *vs.* F_Apt_ | | 0.4374 | | n.s. | F_Apt_ *vs.* PBS | | 0.4374 | | n.s. | |
| PBS *vs.* DSC | | 0.3600 | | n.s. | F_Apt_ *vs.* DSC | | 0.9998 | | n.s. | |
| PBS *vs.* DSCO | | 0.9941 | | n.s. | F_Apt_ *vs.* DSCO | | 0.7246 | | n.s. | |
| PBS *vs.* aPD-L1 | | 0.4171 | | n.s. | F_Apt_ *vs.* aPD-L1 | | 0.0274 | | * | |
| PBS *vs.* DBC | | 0.2579 | | n.s. | F_Apt_ *vs.* DBC | | 0.0147 | | * | |
| PBS *vs.* DBC+aPD-L1 | | 0.1567 | | n.s. | F_Apt_ *vs.* DBC+aPD-L1 | | 0.0082 | | ** | |
| **Fig. 4n CD4+CD25+FOXP3+T cell in spleen** | | | | | | | | | | |
| PBS *vs.* F_Apt_ | | 0.9084 | | n.s. | PBS *vs.* aPD-L1 | | 0.7265 | | n.s. | |
| PBS *vs.* DSC | | 0.5444 | | n.s. | PBS *vs.* DBC | | 0.3828 | | n.s. | |
| PBS *vs.* DSCO | | 0.3548 | | n.s. | PBS *vs.* DBC+aPD-L1 | | 0.1300 | | n.s. | |
| ***p*-value in supplementary Fig. S13, 16** | | | | | | | | | |  |
| **Fig. S13a CD4+ T cell in tumor** | | | | | **Fig. S13b CD11b+CD11c+ DC in tumor** | | | | |  |
| PBS *vs.* F_Apt_ | | | 0.1845 | n.s. | PBS *vs.* F_Apt_ | | | 0.9473 | n.s. |  |
| PBS *vs.* DSC | | | 0.0073 | ** | PBS *vs.* DSC | | | 0.2598 | n.s. |  |
| PBS *vs.* DSCO | | | 0.0228 | * | PBS *vs.* DSCO | | | 0.4165 | n.s. |  |
| PBS *vs.* aPD-L1 | | | 0.0003 | *** | PBS *vs.* aPD-L1 | | | 0.5698 | n.s. |  |
| PBS *vs.* DBC | | | 0.0018 | ** | PBS *vs.* DBC | | | 0.4082 | n.s. |  |
| PBS *vs.* DBC+aPD-L1 | | | 0.0005 | *** | PBS *vs.* DBC+aPD-L1 | | | 0.0377 | * |  |
| **Fig. S16a CD3+T cell in spleen** | | | | | **Fig. S16b CD3+CD4+T cell in spleen** | | | | |  |
| F_Apt_ *vs.* PBS | | | 0.2906 | n.s. | PBS *vs.* F_Apt_ | | | 0.6499 | n.s. |  |
| F_Apt_ *vs.* DSC | | | 0.7482 | n.s. | PBS *vs.* DSC | | | 0.9946 | n.s. |  |
| F_Apt_ *vs.* DSCO | | | 0.2747 | n.s. | PBS *vs.* DSCO | | | 0.9222 | n.s. |  |
| F_Apt_ *vs.* aPD-L1 | | | 0.0618 | n.s. | PBS *vs.* aPD-L1 | | | 0.9861 | n.s. |  |
| F_Apt_ *vs.* DBC | | | 0.0403 | * | PBS *vs.* DBC | | | 0.9966 | n.s. |  |
| F_Apt_ *vs.* DBC+aPD-L1 | | | 0.2906 | n.s. | PBS *vs.* DBC+aPD-L1 | | | 0.8990 | n.s. |  |
| **Fig. S16c CD3+CD8+T cell in spleen** | | | | | | | | | |  |
| PBS *vs.* F_Apt_ | | | 0.4985 | n.s. | PBS *vs.* aPD-L1 | | | 0.4649 | n.s. |  |
| PBS *vs.* DSC | | | 0.7696 | n.s. | PBS *vs.* DBC | | | 0.4834 | n.s. |  |
| PBS *vs.* DSCO | | | 0.4290 | n.s. | PBS *vs.* DBC+aPD-L1 | | | 0.3057 | n.s. |  |
| ***p-*values in Fig.5** | | | | | | | | | |  |
| **Fig. 5c** **Tumor IHC score** | | | | | **Fig. 5d Kidney IHC score** | | | | |  |
| PBS *vs.* aPD-L1 | | | 0.4275 | n.s. | PBS *vs.* aPD-L1 | | | 0.0218 | * |  |
| PBS *vs.* DBC | | | 0.0135 | * | PBS *vs.* DBC | | | 0.1999 | n.s. |  |
| PBS *vs.* DBC+aPD-L1 | | | 0.0184 | * | PBS *vs.* DBC+aPD-L1 | | | 0.0303 | * |  |
| **Fig. 5e Liver IHC score** | | | | | **Fig. 5f Spleen IHC score** | | | | |  |
| PBS *vs.* aPD-L1 | | | 0.8312 | n.s. | PBS *vs.* aPD-L1 | | | 0.4701 | n.s. |  |
| PBS *vs.* DBC | | | 0.7440 | n.s. | PBS *vs.* DBC | | | 0.1044 | n.s. |  |
| PBS *vs.* DBC+aPD-L1 | | | 0.0003 | *** | PBS *vs.* DBC+aPD-L1 | | | 0.4221 | n.s. |  |

***** Statistical significance was analyzed by one-way ANOVA with Tukey’s multiple comparisons test in GraphPad Prism 8.2.1. The level of statistical significance is indicated by asterisks (**p*< 0.05; ***p*< 0.01; ****p* < 0.001; *****p* < 0.0001; n.s.: non-significant).

**Table S6. Summary of the primary cytokines’ concentrations involved in cytokine storms in blood for classic LPS induced mouse model.**

| **Primary cytokines** | **Concentration** |
| --- | --- |
| ^[1-3]^IL6 | 1000~2000pg/ml  (50~100pM) |
| ^[1,2]^IL1β | 800~1000pg/ml  (40~50pM) |
| ^[1,2]^TNFα | 600pg/ml~1000pg/ml  (35~59 pM) |

1. Li, Y. et al. Suppression of Sepsis Cytokine Storm by Escherichia Coli Cell Wall-Derived Carbon Dots. Adv Mater, e2414237, doi:10.1002/adma.202414237 (2025).
2. Meng, Q.-F. et al. Inhalation delivery of dexamethasone with iSEND nanoparticles attenuates the COVID-19 cytokine storm in mice and nonhuman primates. Sci Adv 9, eadg3277, doi:10.1126/sciadv.adg3277 (2023).
3. Sang, D. et al. Prolonged sleep deprivation induces a cytokine-storm-like syndrome in mammals. Cell 186, doi:10.1016/j.cell.2023.10.025 (2023).

**Table S7**. **Flow cytometry antibody panels for animal experiments.**

| Laser | 488nm | | | | 638nm | |
| --- | --- | --- | --- | --- | --- | --- |
| Dyes | AF488 | PE | Percp-5.5 | PE-Cy7 | APC | APC-Cy7 |
| Channels | 525/40BP | 585/42BP | 690/50BP | 780/60BP | 660/20BP | 780/60BP |
| *Treg* panel | CD4 | FOXP3 | CD3 | CD45 | CD25 | Viability stain |
| CD8 panel | PD1 | TIM3 | CD3 | CD45 | CD8 | Viability stain |
| Macrophage/ DC | F4/80 | CD11c | Gr1 | CD45 | CD11b | Viability stain |

**Table S8**. **Serum biochemical analysis.**

|  |  | **Healthy mice** | | | | | **MC38 tumor bearing mice** | | | | |
| --- | --- | --- | --- | --- | --- | --- | --- | --- | --- | --- | --- |
|  | **Normal ranges^[1-2]^** | **NTC** | **PBS** | **aPDL1** | **DBC** | **DBC+**  **aPDL1** | **NTC** | **PBS** | **aPDL1** | **DBC** | **DBC+**  **aPDL1** |
| **Serum**  **ammonia** | **50~150**  **μmol/L** | 105.3  ± 17.2 | 95.7  ± 8.1 | 88.9  ± 5.2 | 103.6  ± 5.3 | 135.8  ± 18.1 | 92.0  ± 7.1 | 112.0  ± 7.3 | 103.1  ± 11.6 | 108.5  ± 17.1 | 174.4  ± 6.1 |
| **CREA** | **10~29**  **μmol/L** | 12.8  ± 0.5 | 12.1  ± 0.5 | 11.8  ± 1.6 | 11.7  ± 0.9 | 13.0  ± 1.4 | 9.9  ± 0.9 | 11.9  ± 3.2 | 11.3  ± 1.5 | 12.5  ± 0.4 | 12.5  ± 0.5 |
| **ALT** | **28~129**  **U/L** | 89.1  ± 5.4 | 71.5  ± 32.3 | 116.4  ± 56.8 | 58.6  ± 24.3 | 71.0  ± 26.1 | 99.6  ± 18.3 | 100.0  ± 67.9 | 123.1  ± 10.5 | 80.2  ± 30.1 | 104.1  ± 16.7 |
| **AST** | **46~392**  **U/L** | 318.7  ± 29.5 | 241.9  ± 58.6 | 379.0  ±158.5 | 226.2  ± 50.7 | 247.9  ± 47.8 | 350.9  ± 94.9 | 280.0  ± 96.3 | 354.3  ± 219.3 | 203.7  ± 46.0 | 254.3  ± 44.6 |
| **UA** | **36~119**  **μmol/L** | 89.9  ± 2.3 | 93.8  ± 5.0 | 75.4  ± 9.5 | 72.3  ± 5.2 | 87.6  ± 10.7 | 87.9  ± 11.5 | 77.8  ± 16.6 | 79.7  ± 8.0 | 90.9  ± 10.9 | 93.1  ± 9.5 |
| **ALP** | **50~500**  **U/L** | 219.6  ± 39.1 | 209.1  ± 19.0 | 189.0  ± 14.4 | 192.6  ± 18.9 | 195.1  ± 12.6 | 202.0  ± 22.0 | 145.1  ± 57.7 | 162.4  ± 4.4 | 180.1  ± 30.5 | 171.9  ± 9.6 |
| **ALB** | **21~45**  **g/L** | 28.4  ± 1.0 | 25.9  ± 1.6 | 23.9  ± 2.0 | 25.6  ± 1.2 | 26.7  ± 0.8 | 24.3  ± 2.6 | 24.6  ± 2.9 | 26.1  ± 2.0 | 27.1  ± 2.3 | 26.5  ± 1.7 |
| **D-Bil** | **0~1.5**  **μmol/L** | 0.5  ± 0.1 | 0.7  ± 0.5 | 0.4  ± 0.3 | 0.5  ± 0.2 | 0.6  ± 0.2 | 0.7  ± 0.1 | 0.6  ± 0.2 | 0.6  ± 0.3 | 0.3  ± 0.2 | 0.5  ± 0.1 |
| **T-Bil** | **0~5**  **μmol/L** | 1.1  ± 0.3 | 1.5  ± 0.8 | 0.9  ± 0.6 | 1.1  ± 0.5 | 1.5  ± 0.1 | 1.0  ± 0.3 | 1.3  ± 0.2 | 1.2  ± 0.5 | 1.8  ± 1.3 | 1.4  ± 0.5 |
| **TP** | **0~80**  **g/L** | 40.5  ± 3.8 | 42.2  ± 2.1 | 39.3  ± 3.4 | 41.9  ± 2.3 | 43.8  ± 0.9 | 38.7  ± 4.1 | 40.4  ± 4.6 | 42.9  ± 3.6 | 44.2  ± 3.1 | 42.9  ± 3.2 |
| **Urea** | **2.8~13.3**  **mmol/L** | 9.9  ± 0.4 | 12.2  ± 2.0 | 11.9  ± 0.1 | 12.8  ± 0.6 | 10.9  ± 0.3 | 12.0  ± 1.6 | 13.8  ± 3.9 | 12.7  ± 2.1 | 10.7  ± 1.6 | 12.9  ± 0.5 |

1. Boehm, O., Zur, B., Koch, A., Tran, N., Freyenhagen, R., Hartmann, M., & Zacharowski, K. (2007). Clinical chemistry reference database for Wistar rats and C57/BL6 mice. Biological Chemistry, 388(5). doi:10.1515/bc.2007.061
2. Zhou X, Hansson GK. Effect of sex and age on serum biochemical reference ranges in C57BL/6J mice. Comp Med. 2004 Apr;54(2):176-8. PMID: 15134363.
